# Insights into the evolution of the *snail *superfamily from metazoan wide molecular phylogenies and expression data in annelids

**DOI:** 10.1186/1471-2148-9-94

**Published:** 2009-05-09

**Authors:** Pierre Kerner, Johanne Hung, Julien Béhague, Martine Le Gouar, Guillaume Balavoine, Michel Vervoort

**Affiliations:** 1 Programme Development and Neurobiology, Institut Jacques Monod, UMR 7592 CNRS/Université Paris Diderot – Paris 7, 15 rue Hélène Brion, 75205 Paris Cedex 13, France; 2 Evolution et Développement des Métazoaires, Centre de Génétique Moléculaire- FRE 3144 CNRS, 1, av. de la terrasse, 91198 Gif-sur-Yvette, France; 3 UFR des Sciences du Vivant, Université Paris Diderot – Paris 7, 5, rue Marie-Andrée Lagroua Weill-Hallé, 75205 Paris Cedex 13, France

## Abstract

**Background:**

An important issue concerning the evolution of duplicated genes is to understand why paralogous genes are retained in a genome even though the most likely fate for a redundant duplicated gene is nonfunctionalization and thereby its elimination. Here we study a complex superfamily generated by gene duplications, the *snail *related genes that play key roles during animal development. We investigate the evolutionary history of these genes by genomic, phylogenetic, and expression data studies.

**Results:**

We systematically retrieved the full complement of *snail *related genes in several sequenced genomes. Through phylogenetic analysis, we found that the *snail *superfamily is composed of three ancestral families, *snail*, *scratchA *and *scratchB*. Analyses of the organization of the encoded proteins point out specific molecular signatures, indicative of functional specificities for Snail, ScratchA and ScratchB proteins. We also report the presence of two *snail *genes in the annelid *Platynereis dumerilii*, which have distinct expression patterns in the developing mesoderm, nervous system, and foregut. The combined expression of these two genes is identical to that of two independently duplicated *snail *genes in another annelid, *Capitella spI*, but different aspects of the expression patterns are differentially shared among paralogs of *Platynereis *and *Capitella*.

**Conclusion:**

Our study indicates that the *snail *and *scratchB *families have expanded through multiple independent gene duplications in the different bilaterian lineages, and highlights potential functional diversifications of Snail and ScratchB proteins following duplications, as, in several instances, paralogous proteins in a given species show different domain organizations. Comparisons of the expression pattern domains of the two *Platynereis *and *Capitella snail *paralogs provide evidence for independent subfunctionalization events which have occurred in these two species. We propose that the *snail *related genes may be especially prone to subfunctionalization, and this would explain why the *snail *superfamily underwent so many independent duplications leading to maintenance of functional paralogs.

## Background

When dealing with the evolution of large gene families, an ideal framework is to use resolved and comprehensive phylogenies both of the species concerned and of the different genes involved. This is even more necessary when one wants to assess the ancestral function of the first representatives of a gene family. Indeed, involvement of some gene families in conserved developmental processes can highlight the evolutionary history of particular structures, but incautious established phylogenies can lead to dubious conclusions especially when paralogous relationships between gene representatives in one species are overlooked. Besides, careful phylogenies of gene families with multiple representatives in many species can yield interesting results concerning the molecular evolution of genes, in particular with regard to gene duplication events.

An interesting example of a large and complex gene family is represented by the *snail *genes encoding C2H2 zinc fingers transcription factors [1,2]. Since the cloning in *Drosophila melanogaster *of the *snail *gene [3] – the founding member of the family – numerous *snail *related genes were isolated in many metazoan species belonging to Arthropods (e.g. [4-7]), Nematodes [4], Vertebrates (e.g. [1,8-15]), non-vertebrate Deuterostomes (e.g. [16-19]), Cnidarians (e.g. [20-22]), and Lophotrochozoans [23-25]. In many species, more than one *snail *related gene was found, indicating the occurrence of gene duplication events. In *Drosophila *for example, in addition to *snail*, five paralogs were found, two with important sequence similarity to *snail*, *escargot *and *worniu *[26,27], and three more, *scratch *[28], *scratch-like 1 *and *scratch-like 2 *[1] that are more distantly related to *snail *paralogs than to each other. Similarly, in vertebrate species, multiple *snail*-like and *scratch*-like genes were identified, e.g. the three mouse *snail*-like and two *scratch*-like genes (reviewed in [1,2]). Based on molecular phylogenetic analyses as well as careful examination of the exon/intron organization of the genes and domain organization of the proteins, it has been suggested that *snail *related genes form a superfamily consisting of two independent families, *snail *(*stricto sensu*) and *scratch*, established early during bilaterian evolution and accompanied by increased complexity through duplication events in several bilaterian lineages [1,2].

The complexity of the *snail *superfamily is exemplified when assessing the different functions of these genes. *snail *genes seem to play numerous and seemingly unrelated roles during development. In *Drosophila*, for example, *snail *is expressed from the early syncitial blastoderm to late stages of development, and is involved in the formation of numerous structures and tissues, such as the invaginating mesoderm[3,29], the anterior and posterior midgut [29,30], the wing, haltere and genital imaginal discs [31], as well as the Central and Peripheral Nervous System (CNS and PNS) in which *snail *acts in both neural precursors, such as Neuroblasts (NBs) and Ganglion Mother Cells (GMCs), and postmitotic neurons [26,32,33]. In several of these tissues, *snail *has redundant roles with its paralogs, for example with *escargot *in the wing and haltere discs [31] and with both *escargot *and *worniu *in the CNS and PNS [26,32,33]. In agreement with their involvement in the formation of very diverse structures, *Drosophila snail/escargot/worniu *genes function in several cellular processes, such as the control of cell shape changes, cell movements, asymmetric cell divisions, cell fate specification and cell differentiation (e.g. [29,31,32,34-36]). *scratch *is mainly expressed in the developing nervous system formation and has been shown to promote neuronal development in the CNS [28].

*snail *genes (but not *scratch *genes) have been studied in a few other protostome species and their expression patterns, while showing some similarities, are not easily comparable with those of *Drosophila snail *genes. In the short germ-band insect *Tribolium castaneum*, a *snail *ortholog expression has been shown in the early invaginating mesoderm, like in *Drosophila*, but no other expression sites have been reported [5]. In the spiders *Cupiennius salei *and *Achaearanea tepidariorum*, a single *snail *gene has been isolated and its expression and function seem to be restricted to the formation of the nervous system [6,7]. In the mollusk *Patella vulgata*, the two isolated *snail *genes seem to be only expressed in unknown ectodermal derivatives [25], while in the annelids *Helobdella robusta *and *Capitella sp.I, snail *genes are expressed in the developing nervous system, parts of the gut, and the differentiating mesoderm [23,24]. Much more is known about vertebrate *snail *genes that have been shown to have multiple roles during development [1,2,37]. This includes control of neural crest specification and delamination, mesoderm specification, left-right asymmetry, the triggering of Epithelial to Mesenchymal Transition (EMT), and the development of limbs, lens and some mesodermal derivatives (e.g. [1,2,9,11,37,38]). In most of these processes, *snail *genes are involved in the control of cell movements and behaviors which has been proposed as the unifying theme of *snail *genes functions, not only in vertebrates, but also more generally in all bilaterians, as in many instances *snail *genes are expressed in migrating, invaginating or delaminating cells and are responsible for these specific cell behaviors (reviewed in [2,37]). Strikingly, expressions consistent with this type of functions have been described in cnidarians where *snail *genes are expressed in the invaginating endoderm of a sea anemone [20,21] and in internalized mesenchymal-like cells of a jellyfish [22], suggesting a possible ancestral role of *snail *genes in the regulation of cell motility among metazoans.

Several previously published studies addressed the evolution of the *snail *superfamily in metazoans [1,2,12,19,37]. In this article, we significantly extend these analyses by systematically retrieving the full complement of *snail *related genes in several newly-sequenced genomes as well as many previously cloned genes from this superfamily. We also cloned two *snail *related genes from the polychaete annelid *Platynereis dumerilii *(*Pdu-snail1 *and *Pdu-snail2*). We conducted multiple phylogenetic analyses on this large dataset and thoroughly analyzed the protein domain organization of the Snail related proteins. Our study allowed us to conclude that (i) the *snail *superfamily can be subdivided into three distinct families, *snail*, *scratchA *and *scratchB*, the latter two forming the larger *scratch *family; (ii) many independent duplications occurred throughout the evolutionary history of the *snail *superfamily and changes in the domain organization of the proteins are associated with some duplications. We also studied the expression patterns of *Pdu-snail1 *and *Pdu-snail2 *and found that these genes are expressed in distinct patterns in the developing mesoderm and nervous system, suggesting bilaterian-wide conservation of *snail *functions in these tissues. In addition, the comparison of *Platynereis snail *genes expression patterns with those of their orthologs in another annelid,*Capitella sp*.*I*, highlights a striking example of expression patterns swapping among paralogs, suggestive of the occurrence of subfunctionalization events.

## Results and discussion

### The snail superfamily in metazoans

As the starting point of this study, we cloned two putative *snail *genes in the annelid *Platynereis dumerilii *by PCR using degenerated primers and RACE protocols (see Methods). We then used these sequences, as well as known *snail *and *scratch *genes from arthropods and vertebrates, as seeds in systematic BLAST searches to retrieve *snail *superfamily representatives in several metazoan species, in particular species for which fully-sequenced genomes are available. This led to the identification of 89 *snail *related sequences (*snail *and *scratch *families altogether) from various species covering the main animal lineages, 10 ecdysozoan species (1 nematode and 9 arthropods – 7 insects, 1 crustacean and 1 chelicerate); 11 deuterostomes (5 vertebrates, 2 urochordates, 2 echinoderms, 1 cephalochordate and 1 hemichordate); 4 lophotrochozoans (2 mollusks and 2 annelids); 2 cnidarians; and 1 placozoan. A summary of the studied species with the number of identified *snail *related genes is shown in Table 1. A list of all the identified sequences can be found in Additional file 1.

**Table 1:** Number of *snail*, *scratchA*, and *scratchB *genes found in the different studied species

*Species*	*Snail*	*ScratchA*	*ScratchB*	Total
*Acropora millepora*	1	-	-	1

*Aedes aegypti*	1	-	-	1

*Anolis carolinensis*	2	-	-	2

***Anopheles gambiae***	**1**	**1**	**2**	**4**

***Apis mellifera***	**1**	**1**	**2**	**4**

***Branchiostoma floridae***	**1**	**1**	**1**	**3**

***Caenorhabditis elegans***	**1**	**-**	**1**	**2**

***Capitella spI***	**2**	**1**	**3**	**6**

***Ciona intestinalis***	**1**	**-**	**-**	**1**

*Cupiennius salei*	1	-	-	1

***Danio rerio***	**4**	**-**	**3**	**7**

***Daphnia pulex***	**1**	**1**	**2**	**4**

***Drosophila melanogaster***	**3**	**1**	**2**	**6**

***Drosophila pseudoobscura***	**3**	**1**	**2**	**6**

*Halocynthia roretzi*	1	-	-	1

***Homo sapiens***	**4**	**-**	**2**	**6**

***Lottia gigantea***	**2**	**1**	**1**	**4**

*Lytechinus variegatus*	1	-	-	1

***Mus musculus***	**3**	**-**	**2**	**5**

***Nasonia vitripennis***	**1**	**1**	**1**	**3**

***Nematostella vectensis***	**2**	**-**	**1**	**3**

*Patella vulgata*	2	-	-	2

*Platynereis dumerilii*	2	-	-	2

*Saccoglossus kowalevskii*	1	-	1	2

***Strongylocentrotus purpuratus***	**1**	**1**	**1**	**3**

***Tribolium castaneum***	**1**	**1**	**2**	**4**

***Trichoplax adhaerens***	**1**	**-**	**1**	**2**

*Xenopus laevis*	3	-	-	3

In bold are indicated species for which whole genome sequences have been used.

Strikingly, in every animal species (except one – *Ciona intestinalis*) whose genome is completely sequenced, we found at least one *snail*-like and one *scratch*-like gene, confirming the ancestry of these two gene families and their strong conservation during animal evolution. An interesting exception is the sponge *Amphimedon queenslandica *in whose genome we were unable to find any gene with significant sequence similarity to *snail *and/or *scratch *genes. Extensive blast searches against several other publicly available databases (such as EST databases) also failed to identify *snail *related genes from sponges (not shown). Sponges are widely considered as the sister group of all other animals (which constitute the so-called eumetazoans) [39] and therefore the absence of *snail *related genes in *Amphimedon *may suggest that the *snail *superfamily evolved after the divergence between sponges and eumetazoans and may therefore constitute a molecular synapomorphy of the latter. Alternatively, *snail *related genes may have been secondarily lost in the *Amphimedon *lineage. This second alternative should be considered as the most plausible if we take in consideration some recently obtained metazoan phylogenies that suggest that sponges may not be the most basal animals [40,41]. We also failed to detect any *snail *related gene in the fully sequenced genome of species outside metazoans, including the choanoflagellate *Monosiga brevicollis*, a close relative of animals (not shown).

Therefore, we conclude that the *snail *superfamily originated early in the metazoan lineage and has been strongly conserved during metazoan evolution. The presence of several *snail *related genes in most species prompted us to further study the evolution of the family by phylogenetic and protein domain organization analyses.

### The snail superfamily consists of 3 evolutionary conserved families (snail, scratchA and scratchB) and its evolution has been shaped by numerous gene duplication events

**Figure 1:**
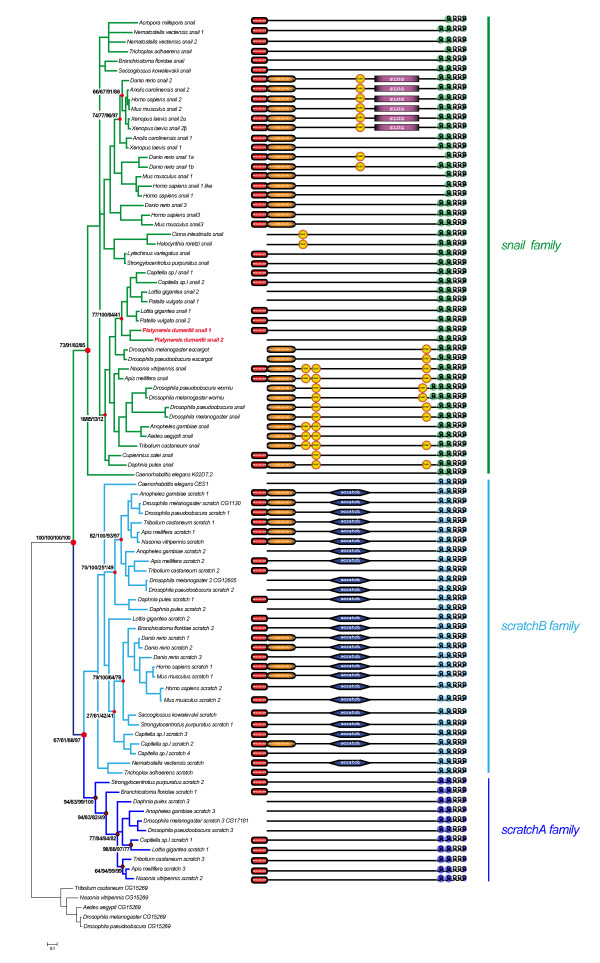
**Phylogenetic analysis and domain organization of Snail related proteins**. The represented tree was constructed by Bayesian inference (BI) and was rooted using the insect CG15269 proteins as outgroup. This tree is based on a multiple alignment that includes the sequence of all zinc fingers, as well as of SNAG, SCRATCH and SLUG domains when present. Red dots highlight important nodes and their associated numbers represent their statistical support values obtained with different methods of phylogenetic reconstruction: first number = bootstrap support in maximum-likelihood (ML) analysis (150 bootstrap replicates); second number = posterior probabilities in BI analysis; third number = bootstrap support in neighbour-joining (NJ) analysis (1000 bootstrap replicates); fourth number = bootstrap support in maximum-parsimony (MP) analysis (200 bootstrap replicates). The asterisk associated with the support in the NJ analysis indicates that in the NJ tree, this node includes the scratch sequence *Caenorhabditis *CES-1. The three monophyletic groups, *snail*, *scratchA *and *scratchB *are highlighted using a color code also used in Figures 2B and 3: green, *snail *genes family; dark blue: *scratchA *genes family; and light blue, *scratchB *genes family. Domains of the different proteins are schematically represented on the right of their respective names.

We constructed a multiple alignment of conserved domains from 89 identified Snail related proteins (Additional file 2) and used this alignment to construct phylogenetic trees using different phylogenetic methods (see Methods). The trees obtained with the different methods were broadly congruent and a representative tree is shown in Figure 1. A similar tree topology was obtained using an alignment including whole sequences (not shown). In order to test the monophyly of the *snail *superfamily and to root our phylogenetic trees, we used as outgroup a family of uncharacterized Zinc finger proteins which are only present in the genomes of insects (known as CG15269 in *Drosophila*) and which show sequence similarity to the Snail related proteins. As expected, we found that all the Snail related proteins form a strongly supported monophyletic group which is separated into two well supported families, *snail *and *scratch *(Figure 1). Interestingly, the *scratch *family is separated further into two subgroups that we named *scratchA *and *scratchB*. Both subfamilies regroup *scratch *representatives belonging to the three main bilaterian lineages (deuterostomes, lophotrochozoans and ecdysozoans), suggesting an early duplication of a single *scratch *gene before the divergence of these three lineages. The presence of a single *scratch *gene in the non bilaterian species *Nematostella vectensis *and *Trichoplax adhaerens *(Table 1) suggests that this duplication event occurred during early bilaterian evolution, after the divergence with cnidarians and placozoans. However, as the single *Nematostella *and *Trichoplax *Scratch sequences have a weak tendency to group with the bilaterian ScratchB proteins (Figure 1), we cannot rule out the possibility that the *Nematostella *and *Trichoplax *genes are *bona fide scratchB *genes and therefore that the *scratchA *representatives were lost in these two species.

We also defined the genomic position of the *snail *and *scratch *genes of every species whose genome is completely sequenced (Additional file 3). While in most species *snail *and *scratch *genes are dispersed to different chromosomes, genomic scaffolds, or contigs, we found a few cases of genomic linkages (colored gene names in Additional file 3). Most of these associations concern either two or more *snail *genes (in *Capitella*, *Drosophila*, and *Lottia*), or two or more *scratchB *genes (in *Anopheles*, *Apis*, *Daphnia*, *Drosophila*, and *Tribolium*), and likely correspond to relatively recent tandem duplications that occurred in some ancestors of these species (see the phylogenetic analysis reported in the two next paragraphs of this section). We also found that the single *scratchA *and *scratchB *genes of *Lottia *are on a same genomic scaffold, but are not adjacent on this scaffold. As such an association is not found in any other species, its meaning remains elusive. Finally, we found that a *snail *and a *scratch *gene are adjacent on a same scaffold in the placozoan *Trichoplax*. A recent study indicated that *Trichoplax *has a particularly low rate of local rearrangement in its genome, as compared to other animals such as arthropods, and suggested that the *Trichoplax *genome may thus have retained some ancestral features in its organization [42]. The linkage of *snail *and *scratch *in *Trichoplax *may therefore correspond to the ancestral situation and point out that the *snail *and *scratch *genes have been produced by a tandem duplication during the early evolution of animals. A consequence of the low rate of local rearrangement in *Trichoplax *is that syntenic regions are observed relative to chordates [42]. Interestingly, we found that, while not adjacent, a *snail *and a *scratch *gene are found on the same scaffold in the cephalochordate *Branchiostoma*. Furthermore, in both mouse and human, one of the *snail *and one of the *scratch *genes are located on the same chromosome (but on different arms, in human, 20q13.1 and 20p12.3-13, respectively). We tried to define whether *snail *and *scratch *may be included in syntenic regions in *Trichoplax*, *Nematostella*, and *Branchiostoma*, but we failed to detect conserved genes close to *snail *and *scratch *in these species (Additional file 4).

**Figure 2:**
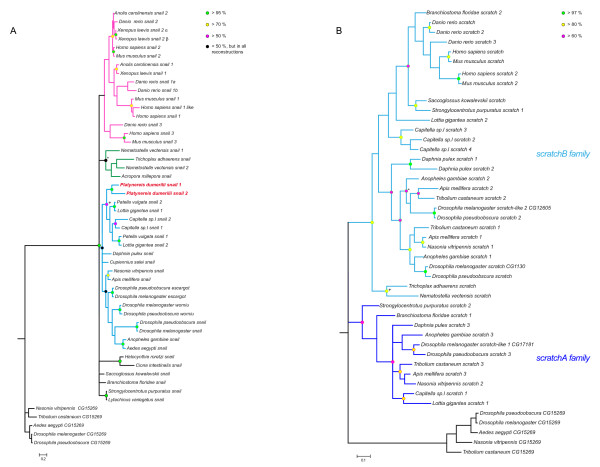
**Phylogenetic analysis of *snail *and *scratch *families**. Trees were taken from BI analyses and rooted using CG15269 proteins as outgroup. (A) Green dots are placed on nodes supported by more than 95% in all different methods of phylogenetic reconstruction, yellow dots highlight support over 70%, purple dots over 50% and black dots below 50% but found in all reconstructions. (+) indicates that this group is not supported in the MP tree. (*) indicates that *Trichoplax adhaerens *Snail was not associated to the cnidarians Snail proteins in the NJ tree. Three poorly supported groups were colored: in purple a group that includes all the vertebrate sequences, in blue all the protostome proteins, and in green the non-bilaterian sequences. (B) Green dots highlight nodes with over 97% statistical support, yellow over 80%, and purple over 60%. (+) indicates a node where the NJ support value was 54%, (*) points to a node where the MP statistical support was 45%.

We then analyzed in more detail the evolution of the *snail *and *scratch *families. For this purpose, we constructed separate phylogenetic trees for these two families (Figure 2). In the case of the *snail *family, the phylogenetic tree is not well resolved as we found only a few statistically well supported monophyletic groups (Figure 2A). The tree topology was similar to that obtained using the whole dataset of Snail related proteins (Figure 1). Resolution was not increased by analyzing whole sequences or just the Zn-fingers (not shown). Strikingly, almost all statistically supported groups in Figure 2A include sequences only from closely-related species (such as mouse and human or the two gastropods *Patella *and *Lottia*) or sequences from the same species (for example in the case of *Platynereis *and *Capitella*). Three groups reflecting deep kinships were nevertheless observed and comprise all vertebrates (pink branches in Figure 2A), all non-bilaterians (green branches), or all protostomes (blue branches) sequences, respectively. The existence of these separated groups, while barely statistically supported, suggest that the last common ancestors of eumetazoans and bilaterians possessed a single *snail *gene and that increased complexity in the family occurred by independent duplication events in the different eumetazoan lineages. From our phylogenetic analyses and applying the parsimony principle, we deduced that at least 10 independent duplications occurred in the *snail *family. Some of these duplications much probably correspond to whole genome duplications (WGD), two rounds of WGD during early vertebrate evolution explain the origin of the three paralogs found in the different vertebrate groups, one additional round of WGD in teleost fishes produced the *snail1a*/*snail1b *pair in *Danio rerio*, and still one more WGD in *Xenopus laevis *led to the presence of the *snail*2α and *snail2*β paralogs in this species [43,44]. Other duplications, in *Nematostella*, Drosophilids, *Platynereis*, *Capitella*, and Gastropods, likely correspond to single-gene (small-scale) duplications. Finally, *Homo sapiens SNAIL1-like *is an intron-less copy of *SNAIL1 *and therefore probably derives from a retrotransposition event.

The two *scratch *families show contrasting evolutions (Figure 2B). In the *scratch*A family, no further duplication event was detected as there is at most one such gene in the different studied species (Figure 2B; Table 1). Strikingly, while a *scratchA *gene is found in both protostomes (ecdysozoans and lophotrochozoans) and deuterostomes (in an echinoderm and a cephalochordate) and therefore is ancestral to bilaterians, this gene is absent in vertebrates and urochordates, indicating its loss after the divergence between the urochordate/vertebrate and cephalochordate lineages. As discussed previously, the absence of *scratch*A sequences in the non bilaterians *Nematostella *and *Trichoplax *could be due to the loss of this gene in these species or indicate that the duplication that gave rise to the two *scratch *families occurred only in bilaterians after their divergence with cnidarians and placozoans. The *scratch*B family is more complex: genes of this family are found in all bilaterian lineages and paralogs are observed in most species (Figure 2B; Table 1). As in the case of the *snail *family, our phylogenetic trees suggest occurrence of several (at least 7) independent duplications in the *scratchB *family: in insects (two genes were already present in the last common ancestor of dipterans, coleopterans and hymenopterans), *Daphnia*, *Capitella *(2 duplications), *Danio rerio *(2 duplications, one of which probably corresponding to the teleost-specific WGD), and Mammals.

We conclude that the *snail *superfamily is composed of three families which are ancestral at least to bilaterians and maybe to eumetazoans. Two of these families (*snail *and *scratchB*) have been broadly conserved in bilaterians and expanded through multiple independent gene duplications in the different bilaterian lineages. The third family (*scratchA*), in contrast, did not undergo gene duplication events and has been lost in the urochordate/vertebrate lineage. We next studied the evolution of the domain organization of the Snail and Scratch proteins.

### Evolution of domain organization of Snail related proteins

**Figure 3:**
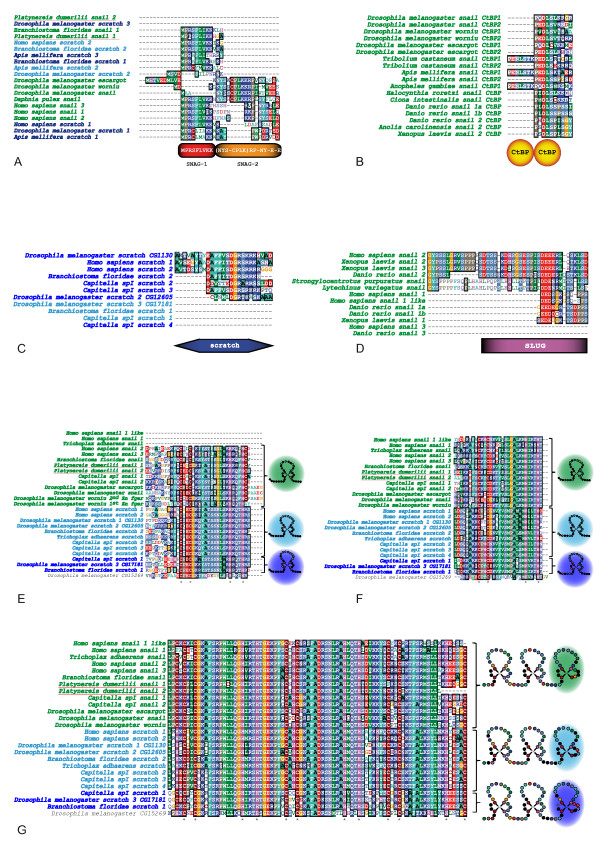
**Conserved domains of Snail and Scratch proteins**. Alignment of relevant sequences illustrating all conserved domains found in *snail *superfamily proteins. Species and gene names are colored using the color code used in Figure 1. (A): Alignment of N-terminal SNAG domains. This 21-amino acids-long domain can be separated in 2 sub-domains (SNAG-1 and SNAG-2) that are rarely simultaneously present in *snail *proteins. (B): Alignment of CtBP domains. These domains are only present in Snail proteins and can be found in tandem or in singleton. (C): Alignment of SCRATCH domains only found in ScratchB proteins. (D): Alignment of SLUG and similar domains found in some deuterostome Snail proteins. (E, F, G): Alignments of the 5 zinc fingers DNA binding domains characteristic of the *snail *superfamily. The first, second and last zinc fingers are specific of each subgroup whereas the third and fourth zinc fingers present an overall conservation throughout *snail *superfamily. Number of zinc fingers can vary by gain (Worniu protein) or loss (*Homo sapiens SNAIL1*) of the first zinc finger. Below alignments, asterisks indicate localization of Cysteines and Histidines forming characteristic C2-H2 motifs.

Snail related proteins bear, in addition to C2H2 Zn fingers, more or less conserved domains found in similar positions inside the protein [1,2,13,37]. From amino to carboxyl extremity, one can find (i) a Snail/Gfi1 repressor domain (*SNAG *domain) supposedly represented in all Snail related proteins (Figure 3A); (ii) binding domains for the Carboxy-terminal Binding Protein (CtBP) co-repressor (*CtBP *domain, present only in some Snail sequences; Figure 3B); (iii) two highly conserved amino-acid stretches considered specific to Scratch and to vertebrate Snail3 (formerly known as Slug) proteins which are the so-called *SCRATCH *(Figure 3C) and *SLUG *(Figure 3D) domains, respectively; and (iv) C2H2 Zn fingers (Figure 3E–G). We used our extensive dataset of Snail related proteins to further study the organization and presence/absence of these domains in the different Snail related proteins. We then examined the evolution of the different domains by representing their presence/absence on the phylogenetic tree of the *snail *superfamily (Figure 1).

The SNAG domain, though initially described as the 20 first amino acids at the N-terminal end of the Snail protein [45], were often restricted in subsequent studies to only the first 8 or 9 residues due to poor conservation of the other residues. Thanks to our large dataset covering many species, we found that the SNAG domain is in fact subdivided into two domains (referred to here as SNAG-1 and SNAG-2; Figure 3A). These two domains are rarely simultaneously present in Snail and Scratch proteins (such occurrence can be observed with *Daphnia pulex *Snail), and more often, only one of the domains is present (for instance SNAG-1 in *Homo sapiens SCRATCH2 *or SNAG-2 in *Drosophila melanogaster Snail*), or the two domains are absent (for example in *Platynereis dumerilii Snail2 *and *Drosophila melanogaster Scratch3*). As the SNAG domain is found in proteins belonging to the *snail*, *scratchA *and *scratchB *families (Figure 1), this amino-acid stretch is most likely to have been present in the protein encoded by the unique ancestor gene that gave rise to the whole superfamily. This suggests that many convergent secondary losses occurred, either of the entire SNAG domain or of the SNAG-1 or SNAG-2 domains. In our most parsimonious scenario, SNAG-1 loss occurred 11 times to explain its actual repartition in our dataset (this domain is absent in 24 sequences). SNAG-2 seems to be even more labile than SNAG-1 and appears to have been lost 18 times independently. Interestingly, variations in the presence of SNAG-1 or SNAG-2 seem to be linked to duplication events, as in several instances SNAG domains are lost in some but not all of the paralogs (Figure 1; for example, *snail *paralogs in Gastropods and *Platynereis*, and *scratch *paralogs in insects, *Danio rerio*, Mammals and *Capitella*). The absence or presence of full or partial SNAG domains is likely to have consequences on the activity of the proteins. Indeed, the SNAG domains which are not only present in Snail proteins but also in other Zn-finger transcription factors, may be involved in the transcriptional activity of the protein, as this domain has been shown, in some conditions, to interact with Ajuba LIM Domain Protein to elicit transcriptional repression [46,47].

CtBP domains contain the PXD/LSX motif required for the recruitment of the co-repressor CtBP by Snail proteins, an important event for the function of these proteins as transcriptional repressors (Figure 3B) [48,49]. We found this domain to be present only in Snail proteins, but neither in ScratchA nor ScratchB proteins (Figure 1). In both vertebrates and arthropods, we often found a CtBP domain in the N-terminal part of the protein close to the SNAG domain and we suggest that this may represent the ancestral situation for bilaterian Snail proteins. In arthropods, duplications of the motif have occurred, as we often observed two or three CtBP domains, one of which can be found in the C-terminal part of the proteins (Figure 1). As this domain is encoded by a single exon and found in many different proteins, exon shuffling may be responsible for these duplications. Finally, the CtBP domain is absent in all Snail proteins from non bilaterians and lophotrochozoans as well as those from some deuterostomes and insects. This suggests that losses of this domain have occurred and that, contrary to what has been proposed [50], the recruitment of CtBP may not be a conserved modality for the function of Snail proteins in animals. However, the CtBP domain is small and may have some plasticity: we found that in two vertebrates Snail proteins (*Anolis *and *Xenopus *Snail1) that lack *bona fide *CtBP domains, a partially similar motif (PXDLTX) can be found in a similar context (same part of the proteins and conserved amino-acid stretches surrounding the motif) than the CtBP domain in other proteins. It is therefore conceivable that these (and maybe other) Snail proteins lacking *bona fide *CtBP domains may nevertheless interact with CtBP through altered motifs – functional characterizations of some of these proteins will be required to explore this possibility.

The SCRATCH domain has been described as a conserved amino-acid stretch (of unknown function) found in Scratch but not Snail proteins and therefore as being of diagnostic relevance for the Scratch proteins (Figure 3C) [1]. Despite extensive efforts, we did not find this SCRATCH domain in all Scratch sequences. Strikingly, while this domain is found in most (but not all) ScratchB proteins, we never found it in ScratchA sequences (Figure 1). The SCRATCH domain should thus be considered as a diagnostic domain of *scratch*B family members (though absent from *Daphnia scratch *2, *Tribolium scratch *2, *Capitella scratch *4 and *Trichoplax scratch *sequences – Figure 2), but not of the entire *scratch *family.

A diagnostic stretch of 29 amino-acids was characterized in proteins formerly known as Slug (Snail 2) and was therefore named SLUG domain (Figure 3D) [13]. While the full domain is only found in vertebrate Snail2 proteins (Figure 1), amino acid stretches similar to part of this domain are found in the single echinoderm Snail protein, as well as in some vertebrate Snail1 and Snail3 proteins (Figure 3D). This suggests that the SLUG domain may have been present in the ancestral deuterostome Snail protein and only well conserved in Snail2 paralogs in vertebrates, as well as to a lesser extent in the echinoderm Snail protein.

Finally, we analyzed the C2H2 Zn fingers sequences (Figure 3E–G). C2H2 Zn fingers bear a conserved #-X-C-X(1-5)-C-X3-#-X5-#-X2-H-X(3-6)-[H/C] residues pattern where X represent any amino acid, and numbers in brackets indicate the number of residues. Positions marked # are those that are important for stable folding of the zinc finger [51]. Alignment of the Zn-fingers allowed efficient sorting of *snail, scratchA*, and *scratchB *family members, with the first and last two Zn-fingers being the most informative (Figure 3E–G). All Scratch and most Snail proteins contain 5 Zn-fingers (Figure 1), suggesting that this organization represents the ancestral situation for the *snail *superfamily. In the *snail *family, the first Zn-finger has been lost in a few cases (in vertebrates and non bilaterians) and duplicated in *Drosophila worniu *genes (Figure 1 and Figure 3E), suggesting some plasticity for the presence of this first Zn-finger.

Our careful analysis of the organization of the Snail related proteins allows the identification of diagnostic amino acid stretches, such as the sequence of the first and fifth Zn-fingers and the presence/absence of some domains (for example the SCRATCH domain), specific of either of the three families and which suggest that Snail, ScratchA and ScratchB proteins have different functional specificities. Our analysis also points to potential functional diversifications of the Snail related proteins following gene duplications, as in several instances, paralogs in a given species show different domain organizations (Figure 1).

### Expression patterns of Pdu-sna1 & Pdu-sna2 during Platynereis development

It is widely believed that paralogs may avoid non-functionalization (and therefore be maintained as active genes) over long evolutionary times only if they evolve at least partially different functions (e.g. [52-54]). This could be achieved for example by evolving different or complementary expression patterns. Our phylogenetic analysis of the *snail *family indicates the occurrence of two independent gene duplications in two polychaete annelids, *Platynereis dumerilii *and *Capitella spI *(Figure 1 and Figure 2). We thought that this may represent a good model to study how genes may evolve, at the expression level, following gene duplication. As the expression during development of the two *Capitella snail *genes have been thoroughly described [23], we studied the developmental expression of the two *Platynereis *genes (that we named *Pdu-sna1 *and *Pdu-sna2*) using whole-mount *in situ *hybridization (WMISH) with RNA antisense probes. *Platynereis *displays an indirect development life cycle with a short embryonic development which gives rise to a ciliated trochophore larva that subsequently metamorphoses into a juvenile worm [55]. Throughout the rest of its life, the worm adds segments sequentially from a sub-terminal posterior growth zone. Due to technical difficulty of assessing gene expression in early developmental stages, we focused our studies on *Pdu-sna1 *and *Pdu-sna2 *expression from trochophore (24 hours post fertilization, hpf) to adult stage.

**Figure 4:**
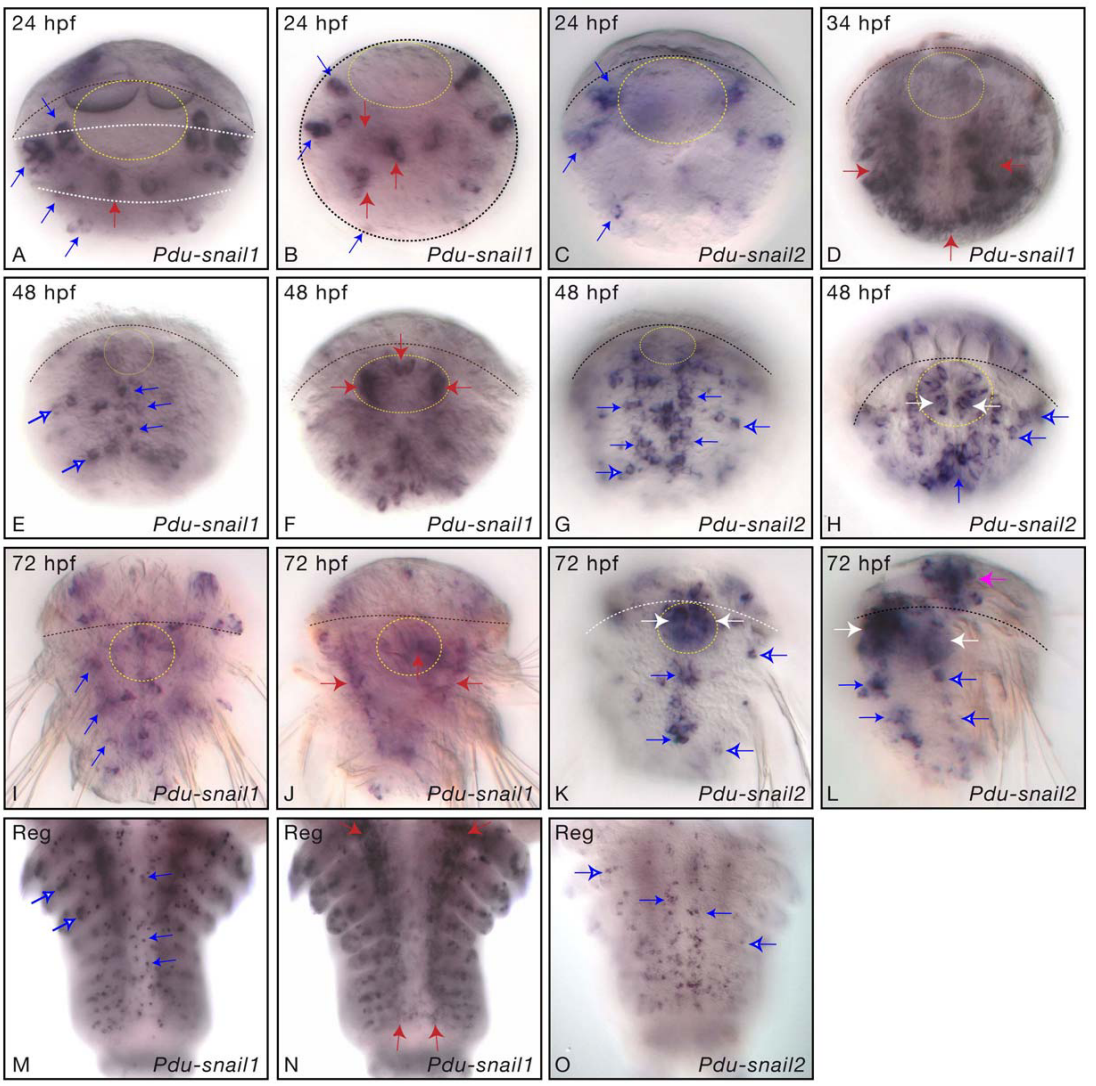
**Developmental expression of *Pdu-sna1 *and *Pdu-sna2***. All pictures are ImageJ projections from WMISH for *Pdu-sna1 *or *Pdu-sna2 *on a selection of larval stages (A-L) and posterior growth (M-O). Posterior growth has been investigated in regenerating posterior part of adult worms (indicated as 'reg'). (A, C-K, M-O) are ventral views with anterior up, (B) is an optical section of the larva (ventral is up) shown in (A) at the level of the trunk (position is indicated by the white dashed lines on picture A), and (L) is a lateral view with ventral side on the left. Black dashed lines delimit the prototroch and the yellow dashed circles surround the stomodeum (closing blastopore in 24 hpf larvae and developing foregut in the next stages). Blue arrows point to neural cells expressing *Pdu-sna1 *or *Pdu-sna2*, filled blue arrows point to cells belonging to the ventral nerve cord and open blue arrows to cells of the peripheral nervous system. Red arrows point to *Pdu-sna1*-expressing mesodermal cells. White arrows point to *Pdu-sna2*-expressing cells belonging to the foregut and the pink arrow to an expression in the developing brain and/or head sense organs.

In 24 hpf larvae, *Pdu-sna1 *is expressed in several bilaterally-organized ectodermal cells (Figure 4A, B; blue arrows) whose distribution is similar to that of the cells expressing neural markers, such as *Pdu-elav *and *Pdu-neurogenin *[56,57], suggesting that *Pdu-sna1 *is expressed in cells of the larval nervous system. In addition, the gene is expressed in internal cells (Figure 4A, B; red arrows) whose organization and position suggest that they belong to the so-called mesodermal bands that will form all the trunk somatic mesoderm derivatives. In 34 hpf larvae, the expression of *Pdu-sna1 *in the ectoderm is similar to the previous stage (not shown), but its mesodermal expression has dramatically expanded, as the gene is now expressed in a large number of trunk mesodermal cells (Figure 4D; red arrows). This mesodermal expression is still found in 48 hpf larvae (not shown) in which three additional expression sites can be observed: *Pdu-sna1 *is expressed in cells that belong to the developing ventral nerve cord (VNC; Figure 4E; filled blue arrows), i.e. the CNS of the worm, in lateral ectodermal cells that probably belong to the developing PNS (Figure 4E; open blue arrows), and in a sheath of cells surrounding the invaginating foregut, i.e. most likely visceral mesodermal cells (Figure 4F; red arrows). All these expression patterns are maintained in subsequent stages, as shown in 72 hpf juvenile worm (Figure 4I, J). At this stage, it is clear that *Pdu-sna1 *is expressed in the differentiating lateral striated muscles (Figure 4J; red arrows). During adult posterior growth, *Pdu-sna1 *displays a salt and pepper expression pattern in the growing VNC, as well as in putative PNS cells in more lateral positions (Figure 4M; blue arrows), patterns that are reminiscent to those observed during larval stages. As in 72 hpf larvae, strong expression is also detected in the differentiated lateral striated muscles (Figure 4N; red arrows).

**Figure 5:**
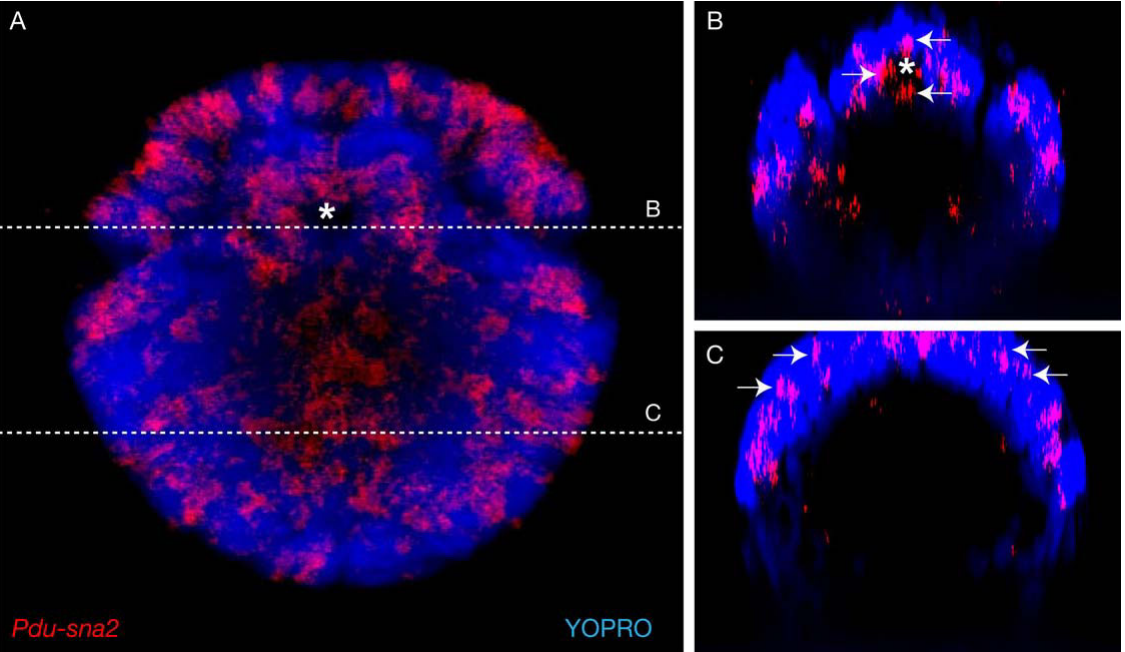
**Expression of *Pdu-sna2 *in neural precursors and foregut cells**. (A) is a confocal picture of a WMISH showing expression of *Pdu-sna2 *in the developing VNC and the foregut. The asterisk indicates the position of the foregut. (B) and (C) are virtual cross-sections which have been made at the levels indicated by the dashed lines in (A). Apical is up, basal is down. (B) *Pdu-sna2 *is expressed in cells belonging to the developing foregut (arrows). (C) Layers of the VNC region have been previously defined [54]. *Pdu-sna2 *is mainly expressed in the intermediate layer (post-mitotic undifferentiated neural precursors; a few of them are indicated by arrows).

In 24 hpf larvae, *Pdu-sna2 *is expressed in a pattern very similar to that of *Pdu-sna1*, suggesting co-expression of the two paralogs in putative larval neural cells (Figure 4C; blue arrows). *Pdu-sna2 *expression is also found in some ectodermal cells in the episphere of the larva, the future head region (not shown) – no such expression was found for *Pdu-sna1*. No mesodermal expression is observed for *Pdu-sna2 *in 24 hpf larvae or subsequent developmental stages. In 48 hpf larvae, *Pdu-sna2 *is expressed in numerous cells belonging to the prospective VNC, as well as in lateral putative PNS cells (Figure 4G; filled and open blue arrows, respectively). While these expression patterns are reminiscent to those of *Pdu-sna1 *at the same stage, *Pdu-sna2 *is clearly expressed in many more cells, in particular in the VNC. In addition, while *Pdu-sna1 *is mainly expressed in the ventralmost cells of the VNC, *Pdu-sna2 *is mainly expressed in more lateral VNC cells (compare Figure 4E and 4G), indicating that the two genes have distinct expression patterns in the VNC with little or no overlap. The 48 hpf larval developing VNC region is complex and composed of at least three layers of cells formed by the superficial proliferating neuroectodermal cells, the slightly more internal undifferentiated neural precursors, and the internal differentiated neural cells [56,57]. Using confocal scanning laser microscopy, we determined that *Pdu-sna2 *is mainly expressed in the intermediate layer and in a few superficial cells (Figure 5A, C), indicating an expression in undifferentiated neural precursors. At 48 hpf, an expression of *Pdu-sna2 *is also observed in cells of the invaginating foregut (Figure 4H and 5B; white arrows) – once more this expression is clearly distinct from the expression of *Pdu-sna1 *in the mesodermal sheath of the foregut (compare Figure 4F and 4H). *Pdu-sna2 *is also expressed in many cells in the head region, probably corresponding to brain and sense organ cells (not shown). Expressions of *Pdu-sna2*, similar to those of the previous stages, are observed in 72 hpf juvenile worms (Figure 4K, L) and during adult posterior growth (Figure 4O). In a 72 hpf, a very large expression is found in the foregut, suggesting that most of its cells express *Pdu-sna2 *(Figure 4K, L; white arrows).

In summary, we have found that both *Pdu-sna1 *and *Pdu-sna2 *are expressed in the developing nervous system, but in at least partially distinct patterns. In addition, *Pdu-sna1 *(but not *Pdu-sna2*) is expressed in parts of the somatic and visceral mesoderm, and *Pdu-sna2 *(but not *Pdu-sna1*) in the developing foregut. The expression patterns can be interpreted along two lines. First, if we compare these expressions with those of *snail *genes in distantly-related species, such as arthropods and chordates, we can highlight putative evolutionary conserved expressions of the genes of this family. A prominent expression in the developing nervous system appears to be such a conserved feature, as it is found in ecdysozoans (for example in insects and spiders; see introduction), in lophotrochozoans (at least in annelids), and in deuterostomes. Indeed, while *snail *neural functions in vertebrates have been mostly described in the neural crest, a tissue considered to be vertebrate-specific, it has been shown that *snail *genes are also broadly expressed in the developing neural tube, in a cephalochordate, amphioxus [18], and in a sea lamprey [12]. These data are therefore consistent with an ancestral role of *snail *family in the formation of the nervous system, both in the PNS and CNS, the expression in the latter having been lost for some vertebrates (gnathostomes). Similarly, expression of *snail *genes in developing mesoderm and mesodermal derivatives is a recurrent theme found in many species belonging to both protostomes (insects) and deuterostomes (vertebrates), suggesting that it may represent a conserved ancestral feature of this gene family.

The expression patterns of *Platynereis snail *genes elicit another interesting line of interpretations when these patterns are compared with those of another annelid, *Capitella spI *[23]. In both annelid species, the two paralogs (which have been produced by independent duplications, see above) are expressed in largely distinct patterns. Taken together, the combined expressions of the two *snail *genes for both species are almost identical, as the genes are broadly expressed in the CNS and the PNS, in the differentiating mesoderm as well as in the foregut, indicating well conserved expressions and probably functions in annelids. Interestingly, these expressions are differently shared out between paralogs in *Platynereis *and *Capitella*. Indeed, *CapI-sna1 *is expressed in the trunk mesoderm, in many cells of the foregut, and in the CNS (including a strong expression in the brain and in the ventral part of the VNC) whereas *CapI-sna2 *is mainly expressed in the CNS (weakly in the brain and in rather lateral cells of the VNC), as well as transiently in a few cells associated with the foregut [23]. In *Platynereis*, *Pdu-sna1 *is expressed in the mesoderm, but not in the foregut cells and only in a limited set of cells of the nervous system whereas *Pdu-sna2 *is expressed in many neural cells (including in the brain), in many foregut cells, but not in the mesoderm. We suggest that the different expressions of the paralogs in both *Platynereis *and *Capitella *are due to subfunctionalization, a process by which ancestral genetic functions (and often expressions) are shared out between paralogs, following gene duplication, and which is believed to be important for the maintenance of paralogs over long evolutionary times (e.g. [52,53]). The subfunctionalization events occurred independently in the two annelid species, as the duplications are themselves independent, leading to different subdivisions of the ancestral expression (nervous system plus mesoderm plus foregut) in *Platynereis *and *Capitella *and therefore different combinations of expressions for the two paralogs in these species. Interestingly, evidence for subfunctionalization has also been reported for vertebrate *snail *genes [12]. We propose that the repeated occurrence of subfunctionalization events may explain why the *snail *family has undergone so many independent duplications which lead to the maintenance of functional paralogs. More precisely, the *snail *genes may be especially prone to subfunctionalization, probably because the ancestral *snail *genes had complex expression patterns and therefore complex regulatory regions, a feature suitable for subfunctionalization [52], and this would increase the retention of paralogs over long evolutionary times.

## Conclusion

In this article, we present a large scale phylogenomic study of the *snail *superfamily in metazoans. Thanks to the use of an extended and comprehensive sequence dataset and several phylogenetic methods, we show a new topology for the *snail *superfamily, with three main families, *snail*, *scratchA *and *scratchB*. Our phylogenetic analyses indicate that these three families are ancestral at least to bilaterians and maybe to eumetazoans. These families have been well conserved in bilaterians as members of these families are found in the main bilaterian branches. Two of these families (*snail *and *scratchB*) underwent multiple gene duplications while the third one (*scratchA*) did not and has been lost in the urochordate/vertebrate lineage. A careful analysis of the organization of the Snail and Scratch proteins encoded by the genome of diverse metazoan species indicates that these proteins, while well conserved overall, show specific molecular signatures, such as particular sequence of the Zn-fingers and the presence/absence of some domains, indicative of functional specificities for Snail, ScratchA and ScratchB proteins. In addition, analysis of the domains of these proteins highlights potential functional diversification of the Snail and Scratch proteins following gene duplications, as in several instances paralogs in a given species show different domain organizations. We also present the expression patterns of two *snail *genes in the annelid *Platynereis dumerilii*, which suggest ancestral functions for bilaterian *snail *genes in nervous system and mesoderm formation. Comparisons of the expression domains of these two *Platynereis snail *paralogs with the expression domains of the two independently duplicated *snail *paralogs from another annelid, *Capitella spI*, provide evidence for independent subfunctionalization events which have occurred in these two species. Subfunctionalization events may have been more generally crucial for the evolution of the *snail *superfamily and may explain the retention of active paralogs in many instances of independent gene duplications.

## Methods

### Cloning and sequencing of Platynereis dumerilii snail sequences

A small fragment corresponding to a sequence conserved among protostome and deuterostome *snail *genes was isolated using degenerate primers on 24 hpf and 48 hpf cDNA libraries and the complete coding sequences of the two *Platynereis snail *genes were amplified using SMART™ RACE cDNA amplification procedures with gene-specific primers. PCR products were TA cloned into the PCR2.1 vector (Invitrogen), sequenced on an ABI automated sequencer, and used as template to produce labelled antisense RNA probes for whole mount in situ hybridizations (WMISH). Primer sequences and detailed PCR conditions are available upon request. Accession numbers of *Pdu-snail1 *and *Pdu-snail2 *are EMBL:FN185991 and EMBL:FN185992, respectively.

### Retrieval of snail and scratch sequences

*snail *and *scratch *genes were retrieved using TBLASTN and BLASTP algorithms [58] on the current assembly and the predicted proteins (if available) of the genomes of the species indicated in Table 1, using the BLAST servers dedicated to these species (Doe Joint Genome Institute, Baylor College of Medicine, Flybase, Genome Sequencing Center, and Ensembl) or the National Center for Biotechnology Information (NCBI) BLAST server (Genomic BLAST databases) [59-64]. Additional BLAST searches were also performed against the NCBI protein TRACE and EST databases in order to identify *snail and scratch genes *in additional species whose genome is not completely sequenced. Amino acid sequences were subsequently predicted using Geneid, Genscan, and TBLASTN against the NCBI nr protein database [58,65,66] or by manual alignment. All the sequences we have identified are available upon request.

### Phylogenetic analyses

Multiple alignments were performed with Muscle 3.6 software [67] and were subsequently manually improved. Handling of the multiple alignments was done using BioEdit sequence alignment editor [68]. Unweighted maximum-parsimony (MP) and neighbour-joining (NJ) reconstructions were performed with the PAUP 4.0 program [69]. NJ analyses were done using the BioNJ algorithm [70] and 1000 bootstrap replicates. MP analyses were performed with the following settings: heuristic search of over 200 bootstrap replicates; MAXTREES set at 3000, and other parameters set at default values. Maximum likelihood (ML) analyses were performed with PHYML [71]. PHYML analyses were performed using the WAG amino-acid substitution model [72], the frequencies of amino acids being estimated from the data set, and rate heterogeneity across sites being modelled by two rate categories (one constant and eight γ-rates). Statistical support for the different internal branches was assessed by bootstrap resampling (150 bootstrap replicates), as implemented in PHYML [71]. Bayesian inference was performed using the Markov chain Monte Carlo method as implemented in the MRBAYES (version 3) package [73,74]. We used the WAG substitution frequency matrix [66] with among-sites rate variation modelled by means of a discrete distribution with four equally probable categories. Two independent Markov chains were run, each containing from 1,500,000 to 3,000,000 Monte Carlo steps (depending on the number of steps required to get chain convergence). One out of every 250 trees was saved. The trees obtained in the two runs were meshed and the first 25% of the trees were discarded as 'burnin'. Marginal probabilities at each internal branch were taken as a measure of statistical support. All the alignments and the trees are available upon request.

### Breeding culture, embryo collection, whole mount in situ hybridization (WMISH), microscopy, and image processing

Animals were obtained from a breeding culture established in Gif-sur-Yvette according to the protocol of Fisher and Dorresteijn [55]. Larvae and regenerated posterior parts collection and fixation, as well as WMISH, were done as previously described [75-77]. In some cases, the NBT/BCIP staining was visualized by reflection confocal laser scanning microscopy [75]. Labeled embryos picture Z-stacks were manually taken on a Leica bright-field microscope and Z-projection images were made using ImageJ 1.36b. Confocal pictures were taken on a Leica Sp2 confocal microscope and images were 3D reconstructed with Metamorph.

## Authors' contributions

PK and JH retrieved the sequences, made the sequence alignments, cloned the *Platynereis snail *genes, and performed most of the *in situ *hybridizations. PK and MV carried out the phylogenetic analyses. JB and MLG performed some of the *in situ *hybridizations. MV and GB participated in the design and coordination of the study. PK and MV drafted the manuscript and all the authors participated in editing of the manuscript. All the authors read and approved the final manuscript.

## Supplementary Material

Additional file 1 **List of all the sequences used in our study in fasta format**. The sequence of the proteins are given. Nucleotide sequences are available on request. Click here for file

Additional file 2 **Multiple alignment of the conserved domains of the Snail related proteins**. This alignments only show the conserved domains of the Snail related proteins and has been used to construct the phylogenetic tree shown in Figure 1. Click here for file

Additional file 3 **Genomic localization of the *snail *and *scratch *genes in species whose genome is completely sequenced** Click here for file

Additional file 4 **Maps of the scaffolds that contain the *snail *and *scratch *genes in *Trichoplax*, *Nematostella*, and *Branchiostoma***. The portion of the genome that includes the *snail *and *scratch *genes is schematically depicted. The name of the genes that flank the *snail *and *scratch *genes are those indicated in the genome browsers of the different species. Click here for file
