# Supplementary material for: Insights into the evolution of the snail superfamily from metazoan wide molecular phylogenies and expression data in annelids
Source: BMC Evol Biol. 2009 May 9;9:94. doi: 10.1186/1471-2148-9-94 (PMC2688512; doi:10.1186/1471-2148-9-94)
Supplement: Additional file 1 — List of all the sequences used in our study in fasta format. The sequence of the proteins are given. Nucleotide sequences are available on request. [file 1471-2148-9-94-S1.pdf]

```
>Acropora millepora snail
MPRSFLVKKKPEKWTKHTLDERKHKGRQNDLQGETLPDSAVIEKCFQQTPVDRPWEGFPS
KCGYPAEGTTSNFGGHVQSVWKTASILCDDATSSNNADTPGNLVEEEDDAMENKISRNDV
DSPGNLNRAKKPQKRERNASTSDVSQRKNKRTRKEFACKHCDKNYLSLGALKMHIRTHTL
PCKCTICGKAFSRPWLLQGHIRTHTGEKPYQCPKCQRAFADRSNLRAHLQTHSSVKKYSC
SQCSRSFSRMSLLVKHQYSCGNEV
>Aedes aegypti CG15269
MSDLNFLPKKHTNFSIENILLRNNATATSKTLELEQKVDLTVKTCIKYERDDLKNATVVL
SPSPASSSSSSQSSNQRPLNRVLANPWCSRGPLMFDPKIIAGAKPSAATAVTAASTVASI
APSSTSPLIPLIKTEVERKVMLSTYANSSVIERNRLSINYPYPVGFFNAYAAAASMATHH
HHNQRQHLPQPSVAVPTVLSSLYQTEKSDENQNSIFSATVNSSGGNLCASGAHHNSDMHS
FFGIDNADMIGYGLVSRQTSPVGTFPIGFPEFSFQCQICDKIFGSEDTLMTHEKTHKSPR
FECEECGKGFSQLRNYKYHISVHRGTKEFAAKCPECGKVFNDKGYLSSHLKIHRNKKEYA
CPHCPKSFNQRVAFNMHVRIHTGVKPHKCNECGKRFSRKMLLKQHLRTHSGEKPYQCSVC
GKSFADRSNMTLHHRLHSGIKPFACPICPKAFTKKHHLKTHLNYHTGYKPYKCPHPNCGQ
TFTQSSNMRTHAKKCQFKPPDQAL
>Aedes aegypti snail
TMPTSIMQKNYSHCPLKKRPVFIREEEDKNETNNLSTKPEDLSMKKKKARSESPVPVVIK
AEDTLPTPPPSSSPDPTEIKSPISVPTPIYGSHPSIYYPPSRSPSSPAEPLHNFYKSAPN
VYPGYPHFPYPMPYPSDLYNLAYHHHISPPRSEPLSPYGQRESSVSPPHPSLYTRQESIS
PPTMNNNIIKSESFARQHRYMPYSLSHHHQLMMPVEHPTLSPTSSHTSFNSYLSSNRSLS
PARSSPSTVSEENNNNTSSPSILTEKSTSNSNIQKVKSEKSSEKSSGSSSGAPRYQCPDC
GKSYSTYSGLSKHQQFHCPAAEGNQAQKIFVCKECDKPYKTLGALKMHIRTHTLPCKCNL
CDKAFSRPWLLQGHIRTHTGEKPFICKLCSRAFADRSNLRAHQQTHEDVKRFKCPSCTKS
FSRLPLLTKHTESGCPGIQLGSQVVPGSPSSHTHQDDKYIPTVLPHSNIACY
>Anolis carolinensis  snail 2
MPRSFLVKKHFNASKKPNYSELDTHTVIISPYLYESYPMPIIPQPEILSSVAYNPITMWT
TTGLLPSPLPSDLSPLSGYPSSLGRVSPPPASDTSSKDHSGSESPISDEEERIQSKLSDA
HAIEAEKFQCSLCNKTYSTFSGLAKHKQLHCDAQSRKSFSCKYCDKEYVSLGALKMHIRT
HTLPCVCKICGKAFSRPWLLQGHIRTHTGEKPFSCPHCNRAFADRSNLRAHLQTHSDVKK
YQCKNCSKTFSRMSLLHKHEESGCCVAH
>Anolis carolinensis  snail 1
MPRSFLVKKHFSASKKPNYSELESQTVIVSPFFYEKYPLSVLPQPDLLTNSPHYPSLVWD
TGLLSNFFTSEPEYQKSAGSPPSPDSKPLDLTSLSSEEEDGKTTSDPPSPASSSATEAEK
FHCGQCSKSYSTFAGLSKHRQLHCDSQARKSFSCKYCEKEYVSLGALKMHIRSHTLPCVC
KICGKAFSRPWLLQGHIRTHTGEKPFSCTHCNRAFADRSNLRAHLQTHSDVKKYQCKTCS
RTFSRMSLLHKHEETGCTGTR
>Anopheles gambiae scratch
MPRCLMAKKWKAYPWPDRAEEQPTGEQEQLDTVPTAGSSHTTTASSPATVDRSSSSSSSS
SGSGAGSSSSSNSSSDASSTSSSPSTMAPECGQPPAASEIVEEDEEIDVVGDSPAAATAT
AASSKSTGSTVATCWGPSSPTAGATAPSPPPHSPEAATRESTILYNGYGQDITHHFTAYL
PKLDPESSSYQQQQQQHQQQQQSSSQQQFVALAPVQHCAIPATASSASPPAAPSAATDSP
QRSPAQKKSLAMCFTSTGTALSLPPKKKDIYRPYSLDDRPPRPSYELRIPAEEDLHAAHA
ILDLSASTAFLPPPAPHQLLAQQQPPSPSVQQSTPAPVVLVVPPKPAIAEPDRRPLQAHP
QQQPPQQQQQLHQTASPPPLPLQSPPATTVPHALGSPEQNENRNSTNTLPGYAAHDATSM
DSSSDELGQSAESSDSEGRNGSKTVAYTYEAFFVSDGRSKKKMADPKTALENKAKYTCTE
CGKQYATSSNLSRHKQTHRSLDSQSAKKCHTCGKAYVSMPALAMHVLTHKLSHSCGVCGK
LFSRPWLLQGHLRSHTGEKPYGCAHCGKAFADRSNLRAHMQTHSIDKNYQCGRCHKTFAL
KSYLNKHLESACFKEDGAPQSPLMSPMSPGSLDGSSLDDLRSTPLSERRQPVALKRERNV
DIEGDESDDAMACPSPLSPVPHHLTISPGAPNGTGSIFVMKNLQSAGRLAGSASPGSGTA
AAAAAAAAAAAAAVLGPGAILTTTTAHKPHHHSPHQQHHPHLQLHQHQHPAAAAAAAAAA
AAAAAAAAVQINVNFAG
```

>Anopheles gambiae scratch 2
MPTGTDYYNKGKSTVLSISIAYGFTIPLSDFRTFKCGPNNSNSNTNLINVKTNSSRHLSV
SASPSQPQPDTGATAPGPIVTIRAAASNGGQQQPPVLLASIGAKSGRNNPNYGIGSSNAN
GPNSITPSPLPATTTATLIYAETTPVAPIVAAAAAAQVSDKRQDKSSLVSSYHKSVTGRQ
QPATLLVQTAATQHAQQQHRTEQQSSAAAMYQPHSQTPATVASSTTTAPACYDTTAAIFR
DRSIEETEAAHDLLSLSQSLPPLTAPCVVTILHQGGTGASSEPLPLQPNAPCQGGIISIV
EAATAANYHATTIKTISPSTAGPLTPPTSEHSSDTECSASSVSPGTSCSRRPHQQPSASS
SVTSVIVNGSALAAGTAPVTRKHKSSTPSTSGGTAAKVPAVSASAKVVAPAPGQKLSGSK
TAELYTYDDLISSDGRSKNRKKASKETAPVQAVAASHSSKAGPPASAASKADGTDQSTAG
DCDSSGESATASNGTSNGKGKYKCPECGKQYATSSNLSRHKQTHRSLDSQSAKKCVTCGK
AYVSMPALAMHLLTHKLSHSCGVCGKLFSRPWLLQGHLRSHTGEKPYGCGHCGKAFADRS
NLRAHMQTHSTDKNFECGRCHKTFALKSYLNKHLESACYKDDDPNGPGPGEGDNSPVSVI
GGRKIYHSSQDIDRDEYAMRMASGRQNYHNDDSCSTTTIDVVTADPGDEDDDEEDIDIIT
T
>Anopheles gambiae scratch 3
MVQEQRPNNVPEELYNLTQLADVSLAAGKLNTANIPLGTVDESQDAVQGYSHKVFDRKKS
RKIRTVSCRSSSGSSEGGTHSESTSQDAVLMHHSGADDQAMKSGEETTTSRPEDVHVCPE
CNKRYSTSSNLARHRQTHRSLEDQKARRCPYCSKVYVSMPAYSMHVRTHDQGSKCPTCDK
RFSRPWLLQGHIRTHTGEKPFKCSVCSKAFADKSNLRAHVQTHSNTKPYQCGRCGKSFAL
KSYLCKHEDSSCLKNDKPVKVRKPTTGSGRSSVRRAKPTRSSERSAPAGSEERSNGVAES
SGTNGLQCGQQASDNRYNYPANVPFKDVLRAKIREVVEDNCKRTARMLAAANGTTPLNGS
VSREPTPQSNRISVIRIAGSPGGYALDGSRPTSSNSSSSSSNSTEYHAESYAVIA
>Anopheles gambiae snail
TMPTSIMQKNYSHCPLKKRPVFMSKDDAMKNDSDGEMEPENLSTKPQDLSMKSMKKKARS
ESPEELVVIKAEDTLPTPPPSSSPCPSDTKSPLSIPTPTYGSLSSIYFPGSRSPAASFPA
VYPGFPYAMPYSSEFYSAYQTQSPHHPAAPLPLHPHHPHHLHPHHSQISPPRSEPLSPYS
AGQHDTSGSPSASPTHLFRPDRHSLSPPPSMVVSAYPTELRLNQNNNIIKSESAFARAQH
RFMPHPHHPAHHHPLMAMSSVAEHPSLSPASSHTSFNSYLSSRGRSLSPISSAANGNSTS
LSSSSGCSSILTEKSTSSANILQKKDGGGEKGASPAGGNGAPRYQCPDCGKSYSTYSGLS
KHQQFHCPAAEGNQAQKTFVCKECDKPYKTLGALKMHIRTHTLPCKCTHCDKAFSRPWLL
QGHIRTHTGEKPFVCKLCSRAFADRSNLRAHQQTHEDVKRFKCPTCTKSFSRLPLLTKHA
ESGCPGGSGPHSQSGGSSAACNISEDSFMPTVLPHGGNIAVY
>Apis mellifera scratch 1
MPRCLMAKKWKAYPWPDRVDDPQQEEENDPSSVLQEGHGGVQQTAASLEKRQHRHEPVED
EEIDVVGDTDTRQVDHQQTCWGPHSPTAGATAPSPPPLNASGALYYHGYTHESWINHEEP
PKYATLRSAAELARPVVPSPPPPAAPQELAAVVTGSSLHQPHPAPTTTPSSATSLSLPPR
KSLSMCFTSTGTALSLPPKKKDIYRPYSLQPTSEIRTSAEEDLSAAQAILDLSASPAAPT
HSVFIHTLSPPPPPPPPPPPPPQQTAPGVAANGQLQPAQLQPPPPPPTAQPIPVSVLVPV
PSSQTNQLRQQEQTPQPQSPATAETNATNCTGGRDGSGSSGSKTVAYTYEAFFVSDGRSK
RRSANTNGAAVPDKEAAQPDRPKFTCTECGKQYATSSNLSRHKQTHRSLDSQSAKKCIHC
GKAYVSMPALAMHVLTHKLAHSCGVCGKMFSRPWLLQGHLRSHTGEKPYGCAHCGKAFAD
RSNLRAHMQTHSADKNYECSRCHKTFALKSYLNKHLESACLRDDEIPQQQQQPHGKDIE
AEKN
>Apis mellifera scratch 2
MPRCYMVKKALCNKYISNVARGFESWGRGRSTPSPTTMQIPVSPIEGSVAPPVAQGTVFV
VPSNYASYNEYLSTQSQDTTTGTKMLEQDVSKGKADEPSNVDMTIATSNTGAPEPPITGR
TEATSPTVITCATTGSSSTIAASQTESIALGVENTTIATTTLPFTAPRSPSRTAHTNYQQ
HTNMPHHSPPGEPSPPSSTATPYHTVDETTDHSASESRIGSNNGNVITNYVSSMFKDRSA
AETEAAHDLLELSRSLPPLPPPSVAIGPQSVIESPATDIQEMTVYQPDQPIYQVNTIDLA
NSTVYTHHHHQQQQTPTASIIYEPSATIVQQTGSVFIPLSPVQEILLTYSTPSIPCAPIV
ATQQPPQQLAQQSHQTIEAAPPLTPPTSECSSDIENNNPNSQPSQKDKEVQTVTEQTEVK
PASYTYDTLLVADGRSKNKKIVPAQKTQETEPVETPETSKIGRYVCCECGKQYATSSNLS

RHKQTHRSIDSQSAKKCIHCGKAYVSMPALAMHVLTHKLTHSCGVCGKMFSRPWLLQGHL
RSHTGEKPYGCAHCGKAFADRSNLRAHMQTHSADKNYECHKCHKSFALKSYLNKHLESAC
QRENDEPNNDLDAPQ
>Apis mellifera scratch3
MPRAFLITHRRYNGVEEEFDGSGRDFSPERGVRLADYSGNHPTSDGASECGSETSSECPE
ELYNLTKLAEVSLAAAAGTLIHPSNVIYQQSASPRCAQFSDKCGRMITPRTKQQEAQFQE
HPRIMEDEQSLGERTRLFFERIECEREILQNRSEKNTVLGIHVPSRTHQEEHRSIEVSEN
SLDKIELESNQSSSSTIFQNRRTTSTSTETSKSEDNEDHECPDCGKKYSTSSNLARHRQT
HRSLGDKKARRCPHCDKVYVSMPAFSMHVRTHNQGCKCNYCGKCFSRPWLLQGHIRTHTG
EKPFKCTICNKAFADKSNLRAHIQTHSNTKPHVCSRCGKAFALKSYLYKHEESSCMRAHH
RSSTDKIDGNDQKIASSSPKSCAPLPSSLSRLQTTSCVTASPTSVIVPRLGLNRDQRNSS
SAFSAIIRHTRVPDASTSPSPSKRSYREKTPSEERERKISDNVTKNPECVSRMVIRTSVI
SPNPERLSRFNNDSQTSSNNYDFSDPTRSSAFSQPTTMTLNLAIA
>Apis mellifera snail
MLVEEVPRSFVKKNNNYSHCPLKKRPVQMLDEEVTEVKEEIEIDVVMDDIPEPENLSTKP
EDLSKTAERNQQQEQEATEQQRPSNGSPSPIPKVSSSPPLTTRPTSPNAALHGHIHPVHH
IHHHHHYPTKVITPPVGIAPIHPVAKKARVEVIQHDNSSSTGAVTATPAPLHFMASKAPL
EPLNLNTPVESLPHYAAPAWARTAPLYPPHYLPYPAAYHRYHPAAADLYPSYPMPAYPHS
SPEHHQAVSPPPHSALTCPTIQRPIARSYAHWTSPDHCGLSPTSSLGSGSLRSPPPVTPE
DLSSPGSDSGRSSAGSTSAGSAIVHPKIEKSGVSSSSTASMSSNSSSSSSSSSSTSPRYQ
CPDCGKSYSTYSGLSKHQQFHCAAAEGQAKKSFSCKYCEKVYVSLGALKMHIRTHTLPCK
CHLCGKAFSRPWLLQGHIRTHTGEKPFSCQHCNRAFADRSNLRAHLQTHSDVKKYSCTSC
SKTFSRMSLLTKHQEGGCPGITVPMGYAS
>Branchiostoma floridae scratch 1
MPRAFLVKRFKHIWESGEAKESEQGEMGEAPEPVTLVPVDWSKGYHPAPFFNPLGLPLVA
PHPDTSPSRRSPVESTALPPSPPPDLHADDLKIKVNDEDILKATTTPNNKHKQTKTKRER
SDSESSVSPRGGETPTGPYHCPECGKRYSTSSNLARHRQTHRDQQDKKARKCPHCDKVYV
SMPALSMHIRTHKQGCKCPYCGKCFSRPWLLQGHIRTHTGERPFVCQICEKAFADKSNLR
AHTQTHSDAKPYVCGRCGQAFALKSYLYKHEESACLRSSGKVTKAATTTRQAQRQGAVDR
RDPQAPI
>Branchiostoma floridae scratch 2
MPRSFLVKKVKAEECSYPAAALQPGPGMAQTCMPRPPQNGQSLTVRLSNGYIHDYIPPVY
PQDAVDCQQSVTATGTGTMVYDIRATANDPPPPLPSHPHAPAQEYTVGYIQCDAFYITDG
RSRRKNGESPARAQRYTCNECGKQYATSSNLSRHKQTHRPLDSKLAKTCPTCGKVYVSMP
ALSMHVLTHQLSHKCDICNKAFSRPWLLQGHMRSHTGEKPFGCAHCGKAFADRSNLRAHM
QTHSAFKQYKCKRCNKSFALKSYLNKHYESACFKQQ
>Branchiostoma floridae snail
MPRSFLIKKKLHHAAKKPRFDDSVIKPFPELATIEPGLVVPPPAVWPVPLCYPPDAVMEL
GGLPPLPLSPEPPARASPPLPMEAELPGGETHHDKPMKKRTPSGGSGDQSRYQCPQCAKS
YSTYSGLTKHKQFHCVTQSKKAFNCKYCDKVYVSLGALKMHIRTHTLPCKCKICGKAFSR
PWLLQGHVRTHTGEKPFACPHCSRAFADRSNLRAHLQTHSDIKKYSCKNCSKTFSRMSLL
TKHEEAGCCLPNH
>Caenorhabditis elegans CES1
MITTPNISLTPTVAPPVDFPIIGNCDFQAQLWHLMSLHLQMQKSSAASTSSSSSSSTSSE
NLKKSPSSVQNTSVFSIDNILNSQKVPKLELENDEDVSSSPSPTCSTTGYTLDSLQNLDR
RSLNKKGPSSHNRCVCDKCGKSYATTSNLSRHKQTHRALDSPHAKQCPHCDRVYVSMPAL
SMHILTHNASHECNVCGKRFSRLWLLQGHLRSHTGLRPFSCAHCGKSFADRSNLRAHMLT
HTGDKRFECDKCGRRFALRAYLNRHLETCK
>Caenorhabditis elegans K02D7.2
MSINTKRKSIFETIEDLISPDPAPSSSSSSASCSSLDNQKLVCQFCKKTYLTYFGLRRHL
QFHKEGKLQQSCPHCKKVYRSPGALKMHLKTHSLPCVCNDCGKSFSRPWLLKGHLRTHTG
EKPFGCEFCGRCFADRSNLRAHLQTHSGEKKHRCSRCGQSFARVQVRQRHEQCCRGGGSG

GEAEKEDVEVDGD
>Capitella spI scratch 1
MPRAFLITNKRYRPGARLPAGDKELKESISISVGYKQRKRPKTARFVFYGDWTQRVITPS
SLWLLNATKTERRASGKQTGSLFYAGHLYKYEASIWEEFVRGDIDLDAKNDLPPLPPASP
LVTPTTMTSPSPVTSPSPVTSLSPVTSPSPITSPSPLTSPSAIPGNAPFNPPFLLPPGGL
DCTLSPSKASIPSLSPCKPDSPSHSSRVLYTPPNLNPLLFSPLKREPPPRIPFPLPKWPT
AFNPLFGLTDLSDLHQTTLIPSSNVSPQYMESRMNNFVWGSSPDITVPGQIEEHHPCPDC
GKAYSTSSNLARHRQTHRSISDKKARKCPHCDKVYVSMPAYSMHVRTHSQGCQCPYCGKR
FSRPWLLQGHIRTHTGEKPFSCQQCGKAFADKSNLRAHVQTHSTEKPYSCGRCGKAFALK
SYLYKHEESSCMRGQRLHGRTPAFR
>Capitella spI scratch 2
MPRSFLVKKRPRWSLSDNPTWPEQQPCYQPYFVPQPAREQPATTGLTVLKPLSNWNCLNT
PIARTVKRKAFDEDSGIESATESGPESPALPKPEKDDVTTEDGRARRSSLDDVKAKYTCT
ECGKQYATSSNLSRHKQTHRSLDSQQAKKCPHCNKVYVSMPALSMHILTHNLKHECPVCN
KTFSRPWLLQGHMRSHTGEKPYGCAHCGKAFADRSNLRAHMQTHSTFKHYECTRCSKTFA
LKSYLNKHCESACPKIDFEDTENH
>Capitella spI scratch 3
MPRSFLVKKTERKQSHNVFRVRLIIILNSGFFARIIIFSFVILFVNKLIPSHPYSPLQFRP
PRPCLFALFCDGYPAASFGILHAKVKKKEEEEPKTTGGKFVCNECGKQYATSSNLSRHKQ
THRSLDSQQAKKCPHCNKVYVSMPALSMHILTHNLKHECPVCNKTFSRPWLLQGHMRSHT
GEKPYGCAHCGKAFADRSNLRAHMQTHSTFKHYECTRCSKTFALKSYLNKHCESACSKDD
T
>Capitella spI scratch 4
MPRSFLIKKHADKQKKKEPTQAADAGRSLALPVSPTPGLEAFLPPAYLPGLRHPASLHPG
EPLLPQVSPFTPLIQPLALRLCTGYFHDFLPHHAARIPFYDNPFHPLPPLRPPPPLDLKL
SPPPPTKPVATVSPKKPETDDEKAKVKEEEDDENEEKQKGKGHGFQDGVTVGYTYDAFFV
SDGRSRKRKPTQDVKPKYTCTECGKQYATSSNLSRHKQTHRSLDSQQAKKCPHCNKVYVS
MPALSMHILTHNLKHECPVCNKTFSRPWLLQGHMRSHTGEKPYGCAHCGKAFADRSNLRA
HMQTHSTFKHYECARCSKTFALKSYLNKHHESSCLKDEGMLTSPPRWKSVEAARG
>Capitella spI snail 1
MPRAFLIKKTERSESDGAITRITSSDKTSPSAEFRAMESISTDDEHAATTEEEIDVVTED
ADEPKSTGHDAPSPVCRSPPPDTGGHDSRTRRSAFGIYRFLSHMKPPSEHQVHDGKEPWL
PGVPLPDKFHPSPDALHLRPPAVPSAMDSPPAGSTPLNLGAFRLSPAGVNPGYLLGFPGG
SPGATPLHPQSPLPPNTFLPFPPHTFLSSYGHPLTGPPSLRLAPFPFQYGLRHDVTRQLH
SFHGHHVKSESSKRASPVNWLLDSSPTKLGKRKEDGGGEEEEEEDCRLHVSATDKRNGPP
RYQCDACKKSYATFSGLSKHKQFHCATHIKKEFSCKYCDKTYTSLGALKMHIRTHTLPCK
CKLCGKAFSRPWLLQGHIRTHTGEKPFQCAHCGRAFADRSNLRAHLQTHSEVKKYRCKTC
AKTFSRMSLLIKHQESNCPGLRPPSVATN
>Capitella spI snail 2
MPRAFLIKKSTAEAKLKILPDQPHSIDQLIGRRDKLLENMDSFARRELTSHDEEQDYVDI
EADDEEECSSKISDNTDRTSPFPSDDGRPGSAGSSRNSQFTPSPRPVPVRPWELGHPTPV
HPTAMTLTSPVCHMEKHHPAEAADAKLPHLQSPHLRYAGFPLPYPFPYDMPDMWNNNMKS
VHGLVNYFDPAYLRMLYRDHQQPHAPTPLLPFPSLRPDKTNERMTWATSSSSERSSPVGG
GVSPPVLEEELMKKEKKRDSKAGHFQCDTCKKSYSTINGLTKHKQFHCEDMMKKEFSCKY
CDKTYTSLGALKMHIRTHTLPCKCKLCGKAFSRPWLLQGHIRTHTGEKPFQCAHCGRAFA
DRSNLRAHLQTHSEVKKYRCGRCSKTFARMSLLVKHEGRECLSSI
>Ciona intestinalis snail
MTSVEPMLYQGKHAVQKNEGESTRPCTLSGDDSFYCSGESTNSTSSPTSSITSSRSCGPD
SDEGFPRQDDLDLRDKASKNLQRSVELFCPIKSKSVDHDNDLMPMDLSCKKRTSPKQNCK
TASMPKTSPTIKSEPIDDYPASLTRNAPPSSMPSVSPPSSNITREFPPSMFPSWPYFSTP
ITSSVGGFPSFPSSYIAGKYLHPALFLPPPATSCQTVPTNSPLGLSVGNSMLPGLHQLAA
SHFQPSMIKPVAQPQGVPQEQNSPNHDDQKFAQGSPQPRFSPTNLVQDPSLLAEFARVFS

RQVEQFRPKPSFEENNMKNQNSERRRKNAKPLKISADVSPPHPQLNDMRSISFKDLPTMV
SQTHDHTAFYGAQKNRQELKRKSSSEDNSESPTGKKVCLDSKTTWRQIDAPTFQISDAIE
EQNKAPQPVSFKPCRIPCTECGRTYATIGALAKHAKTHEDPESGSKFNCKICKKECSSLG
ALRMHIRTHTLPCECHICGKAFSRTWLLQGHIRTHTGEKPYQCTVCSRAFADRSNLRAHM
QTHETVKRYSCVTCEKTFSRISLLKRHQVHCETASQVAQRKTAS
>Cupiennius salei snail
MPRAFLIKKKQQCAKNGQSLARTNWLEDSDNMDSSRDNPQFTPLTIVAPDTKGPYDLSMK
PKNFDESSNDSEQRLVISPRPTINISSHPQGTLIAPKPIKPTPKTPDEIAASRSHWQKQM
MSPYLPFNYPVYAYPGRPSEIYPFGNNYMSSQNSMVPPPLVPLSSASSNVDRYSPTRDRY
EVPPRRAVSPVVAMSGSPSPPAVFPGWYSDGQDSGLASSPSPSSEDGEAAASKPNPTRYQ
CPDCNKSYSTYSGLSKHRLMHCATQAKKSFGCKYCDKVYVSLGALKMHIRTHTLPCKCKL
CGKAFSRPWLLQGHIRTHTGEKPFSCPHCSRAFADRSNLRAHLQTHSEVKKYRCKTCSKT
FSRMSLLLKHEDGGCAGAAASQQQPPVNTANYA
>Danio rerio scratch 1
MPRSFLVKKVKLDDFSSSDLESSYGRSRADISLRFHEKAYISDYMTPAPYDGESDSGIKV
PSPGPIYDSIHSDYGAPDSDQPDSPQSEISSGYINGDTAVSEGYTVDAFFITDGRSRRKA
ISSPRTLQRHTCNECGKTYATSSNLSRHKQTHRSLDSKMAKKCPTCGKVYVSMPAMAMHL
LTHDLKHKCDICGKAFSRPWLLQGHMRSHTGEKPFGCAHCGKAFADRSNLRAHMQTHSAF
KHYKCKRCNKTFALKSYLNKHYESACFKGAFAPLSPMEV
>Danio rerio scratch 2
MPRSFLVKKVKLDDFSSSELESAYGRSRTDLSFRIHDKGYISDYITPAIYDGESDGGTKV
PSPGPIYDSNHSDYGAPDSDQPDSPQSEITSGYINGDNAVSEGYTVDAFFITDGRSRRKV
ISGSRTLQRHTCNECGKTYATSSNLSRHKQTHRSLDSKMAKKCPTCGKVYVSMPAMAMHL
LTHDLKHKCDICGKAFSRPWLLQGHMRSHTGEKPFGCAHCGKAFADRSNLRAHMQTHSAF
KHFKCKRCNKTFVLKSYLNKHYESACFKGAFSPHSSIEA
>Danio rerio scratch 3
MPRSFLVKKIKLDDFSSSPVSNHHHHHHNDRHMDDSFSRSRSSLGVRLCENGYIKDYISS
SEYTEEKQADMKLNSELLYSPVSSGGGEYCQPDLEHPDSPQSGLTARGYFSSESESLSEG
YTMDAFFISDGRSRRKGEVSEAAKADEVEKEVVGVNNGGARHTCNECGKTYATSSNLSRH
KQTHRSLDSKMARKCPTCDKVYVSMPALAMHILTHDLKHKCHVCSKAFSRPWLLQGHMRS
HTGEKPFACAHCGKAFADRSNLRAHMQTHSAFKHYSCKRCNKTFALKSYLNKHYESACFR
GSGDEDESGSEN
>Danio rerio snail 1a
MPRSFLVKKYFTSKRPNYSELECQNDTSPDRYPLAELPAVSNDFPVTCLTTGLVWDVSLL
PSLHNSPSPSTLSTNQGPLDLSSPSSISCSSSGEEDEGRTSDPPSPPSPDSSDTYHPQQT
SRPRRSNKSRAGQREDKSEAAVTAASRPAFFCKHCPKEYNSLGALKMHIRSHTLPCVCPT
CGKAFSRPWLLRGHIRTHTGERPFSCPHCNRAFADRSNLRAHLQTHADVKKYQCSTCSRT
FSRMSLLQKHSAAGCCPSTANVQ
>Danio rerio snail 1b
MPRSFLVKKYFTNKKPNYSELESQTDRRYAVVPQCFPLDDPLVPKYPSMLVWSSSALPFP
GVSSSVSCPPAPLDLSSPSSSSSSGEEDDCRTSDPPSPDPSDRFQCAHCGKSCSSPAALS
RHQLAHCSPQDGISGATSSLTSSRAAFHCKHCPKEYNSLGALKMHIRSHTLPCVCSTCGK
AFSRPWLLRGHIRTHTGERPFSCPHCNRAFADRSNLRAHLQTHSEVKKYQCGSCSRTFSR
MSLLHKHTLSGCCPAL
>Danio rerio snail 2
MPRSFLVKKHFNAAKKPNYSELESPTVFISPYVLKALPVPVIPQPEVLSPVAYNPITVWT
TSNLPLSPLPHDLSPISGYPSSLSDTSSNKDHSGSESPRSDEDERIQSTKLSDAEKFQCG
LCNKSYSTYSGLMKHKQLHCDAQSRKSFSCKYCEKEYVSLGALKMHIRTHTLPCVCKMCG
KAFSRPWLLQGHIRTHTGEKPFSCPHCSRAFADRSNLRAHLQTHSDVKKYQCKNCSKTFS
RMSLLHKHEESGCCIAH
>Danio rerio snail 3
MPRSFLVKKHLTNKKPDYGVLDSKKHEMIHSESSNSKLKTLHPHQNMFPVPCYGNSAGWM

NPISSDIYMPPQHPSLPIDDPILGLTYPLPAPSSPLRDMRPALSMLEHTDPSSLQLSHRD
LHEKVPVSPLGLTTTGNSQEHSDECFDCQKAYLSFSNLANQRQVHCQWPCHKYFTCKYCE
KEYVSLGALKMHIRTHTLPCVCKLCGKAFSRPWLLQGHIRTHTGEKPFTCPHCSRAFADR
SNLRAHLQTHSEIKKYQCRNCFKTFSRISLLTKHEEAGCCPMS
>Daphnia pulex snail
MPRSFLVKKNQNYSHCPLKKRPLSYFLQESLEAESVAATEASLVTSAMSEEPENLSLKPE
DRYAAAERERQNQKRLRTLLEASAAVGSSMTPAALSPPPAHQPLKSWTPPLGHHHHHHY
APEHSVSHIICQASSSPSLAAKLSTPLEPLHLNNQINVAVPYHHHHHSHHSTQHQYAPYL
PFNFPAYRSFSTAAISPLYPAVAASNSSPHRPSSEQLSPFAPSESASQQQSSPLRVQWPE
SVAISSAVALKLSESTSDIEDLSSPASGSSGSSGCEESGTESLLHHHHHHPHHGHHHSPA
AAGLVRSNKKSGSSASSTSSASSASSASSTSAGTTTTARYQCPDCHKSYSTYCGLTKHQE
LHCAAQQAANKKSFSCKHCEKVYVSLGALKMHIRTHTLPCKCLLCGKAFSRPWLLQGHIR
THTGEKPFSCPHCSRSFADRSNLRAHLQTHSDVKRYSCKSCGKTFSRMSLLSKHEDGGCG
CGGASSHASSSGSSSASSPSPNGAQQQQLLAVAKREVA
>Daphnia pulex scratch 1
MPRCFMPKKHLKNRPGMTERSNGGRRSPSPCVDLPPPPPPPPLLSPPPPPPASHETNGDD
ADPETNASNAVSAKSGSNVYRPPTPRRSPASGPAVHRRIVQLSPPAPPSPASPPTVAAAA
GEWCDDGPGAAGLEHGPRHPRLKHGSHPLQPAATAPPPSPPPRRGRVRESHGNHARQFQD
AASVAGQFGPGRSQSNTSGPGKRLDFVVVPLQQQQQQFEQLYRHKTVAYTYEAFFVSDGR
SKRRNNPSSQQQPSSASSSPSPNPSSRSGRYVCGECGKAYATSSNLSRHKQTHRSLESGC
ARTCPTCGKAYVSMPALSMHLLTHALSHVCPVCSKAFSRPWLLQGHMRSHTGEKPYGCAH
CGKAFADRSNLRAHMQTHSATKSHHCGKCHKAFALKSYLNKHLESSCFRDSPTPSHSESS
CDSMGAESHLLPVDAKVSKPVPVT
>Daphnia pulex scratch 2
MRHVDNGDGHHRRYVCGECGKAYATSSNLSRHKQTHRNLESGGSKPCPTCGKTYVSMPAL
SMHLLTHALTHVCPVCSKAFSRPWLLQGHMRSHTGEKPYECHLCYKAFADRSNLRAHMQT
HSSVKSFRCGQCHKTFALKSYLHKHQETSANCCVKT
>Daphnia pulex scratch 3
NNAAAIPEEMYNLFQLAEVSLAASGGSEFRSVLERWRHQQQGLVMARALKSPVVKILDMS
AAAAAANCAGHSPIPVLDKPLDLSRDPATITTATATATEVRILTPSPSPTPSETHLTINL
GLRKKLRPQTTSSSSGHRGSSEADDDDEDDDEEEDDEEDDELDATDSCWSPDDVASCKSV
SVVVKGPSISTGSNSGDSHSGPDGHECPDCGKRYSTSSNLARHRQTHRSPADQKARRCPH
CDKVYVSVPAFSMHVRTHSQGCKCPYCGKSFSRPWLLQGHIRTHTGEKPFTCQICEKAFA
DKSNLRAHIQTHSNLKPFTCQRCGKAFALKSYLYKHEESSCMKLVTKITADADPKDDSIM
RERVKERHQIIEYDQP
>Drosophila melanogaster CG15269
MLQRSYDLSPSPARTSPTNGVQNLGAGAQLPPAQVTDFSISKILGKERKSAQDTSTNILD
LSKSSSNSIRSPQALGGLAIPSTPYASGAYSMLPPDLVAMANATKFYAQFFPHLLPAYAA
AAAAAGLSTPPTSPYQTHQHQQHLPNQQKRFFAPYVINGSVPPPPPLQQQQQPLIRTKST
ACTRPDCPECLDYYQRLQTGGYKQTAPLISPAASSVSSSTGSIRPVRDLIMSATGGAAST
NISLTTVTNLSLNSVQATAVGMMPKMTGGLITTAVGSNSGAIGGIGGYNATSAEEISYKC
RICEKVFGCSETLQAHEKTHKSPRYECADCGKGFSQLRNYKYHLSVHRGTKEFAAECPEC
GKTFNDKGYLSSHLKIHRNRKEYECPYCPKSFNQRVAFNMHVRIHTGVKPHKCNECGKRF
SRKMLLKQHMRTHSGEKPYQCSVCGKSFADRSNMTLHHRLHSGIKPFSCPLCPKAFTKKH
HLKTHLNYHTGCKPYVCPHPNCNQAFTQSSNMRTHAKKCQYRPLDGLTVTSSALPVPGKQ
QTGPPPTLAMAMAQTFQMPPPPGVLTPGSGPSQPPSQQTLLSNLRTF
>Drosophila melanogaster escargot
MHTVEDMLVEKNYSKCPLKKRPVNYQFEAPQNHSNTPNEPQDLCVKKMEILEENPSEELI
NVSDCCEDEGVDVDHTDDEHIEEEDEDVDVDVDSDPNQTQAAALAAAAAVAAAAAAASVVV
PTPTYPKYPWNNFHMSPYTAEFYRTINQQGHQILPLRGDLIAPSSPSDSLGSLSPPPHHY
LHGRASSVSPPMRSEIIHRPIGVRQHRFLPYPQMPGYPSLGGYTHTHHHHAPISPAYSEN
SYYSMRSMTPESSCSSSLPEDLSLKHKNLNLNLNTSQPGEQAAAKTGDMSPETMPNASAK

KDKNQPPRYQCPDCQKSYSTFSGLTKHQQFHCPAAEGNQVKKSFSCKDCDKTYVSLGALK
MHIRTHTLPCKCNLCGKAFSRPWLLQGHIRTHTGEKPFSCQHCHRAFADRSNLRAHLQTH
SDIKKYSCTSCSKTFSRMSLLTKHSEGGCPGGSAGSSSGSELNYAGYAEP
>Drosophila melanogaster scratch CG1130
MPRCLIAKKWKAYPWLDRTEDTSNQQQQQEQSAPNSPRELEELHLKSRRSTLDDDEEIDV
VGDKFLIKLEKQRTTADAAAAAATSSEAATSHSSNSSNMEASATTTTSKCWGPSSPTAGT
TAPSPPPHSPEAATRVAGNVYNGYTRELSPLHYTAYLPRMESEITVIRAAATALVAARTS
GNGSGDQHLAAYQTPPSSTTSSPSCSPSGAGDRYSPLSSGQTSSERKCFSSTGATLSLPP
KKKDIYRPYSLDDKPAHGYRRRVPAEEDLHAAHAILDLSASTAFHPPTQPHQLQQQQQQQ
QQQHQHHHSQQQHLAPQQHHYLPLQQQQQQQAHHTHLPTLEAHAHLRSTSSIAELAAAAS
VVNEQRPASNASSASSNHMPSSPSSNSSSSSSQVQNENSNTTNTNPDGDGCLQDGEHSGA
SGASAKTVAYTYEAFFVSDGRSKRKHVADPAAAASGVPTPDQQKTKYTCSECGKQYATSS
NLSRHKQTHRSLDSQSAKKCHTCGKAYVSMPALAMHLLTHKLSHSCGVCGKLFSRPWLLQ
GHLRSHTGEKPYGCAHCGKAFADRSNLRAHMQTHSVDKNFECKRCHKTFALKSYLNKHLE
SACLKDEEELMMSMSLSMHDSNSESGASMASSPPHEFLERVKLEGSGGATYAM
>Drosophila melanogaster scratch CG12605
MSVDFILPNRNKIQTIAYASASKKYVPGGSGSTLSSGSVSASGADLQSWRRPKPRTSGKV
KFDDKSPLTASSVLKNANGNGLGTGGKSPLKSLAKRNSRSILKREIIDLDDEDGEEQEDQ
LCEHLEWSAAQSLVQMNSSKQEKQRRAGTTGSPATVAAPVSASVSLRRPVGRAPAEDGKV
IFYAPPASASGSPRQPEPVALKRERERDKERERERKERERERDRMREQQLVAATAAASIYRGR
SVEETEAAHDLLSLSQSLPPLIPPCVVTIMKQEQEQLRSPEIQEISNSASSRSPQSTIRF
IGSSSYDLMGGSSEGANNCSPLTPPNSDHSSDVDIDMSSSSESGLQQWGKPNPQQNQQRA
SALKMCLNMLDGRTKASKAAAVTKQDRQEPMQPRPKSAQSNASSGGGPPSEPPENPAASL
GHSSSGNGENYAKRKRGCYKCCECGKQYATSSNLSRHKQTHRSLDSQSAKKCNTCGKAYV
SMPALAMHLLTHKLSHSCDICGKLFSRPWLLQGHLRSHTGEKPYACVHCGKAFADRSNLR
AHMQTHSGDKNFKCHRCNKTFALKSYLNKHLESACLRDAGAIDQGKGLEEEDDDCSKQDE
LEMGDELESDENSQDIVVA
>Drosophila melanogaster scratch (CG17181)
MTKDQQATPIPEELYNLTQLAEVTLSVGPLVTDEVKPLPLYASSDDDSNYYSHKVFDRRK
LRRCTISDSNSCASSSSSSTSSRQSSEDHLGLQGHSSVHHHHGEQGEILNSTSLLEDEHI
CPECGKKYSTSSNLARHRQTHRSIMDKKARHCPYCEKVYVSMPAYSMHVRTHNQGCECQF
CGKRFSRPWLLQGHIRTHTGEKPFKCGVCEKAFADKSNLRAHIQTHSNTKPHTCARCGKA
FALKSYLYKHEESSCMKNRGGVPGSGAASGNRPPSSPKRQQAEVTSGTISALAPGSPAAA
VCAASDSAKSTLANKLLQKEKDRRQAAMAFQGFPAGPEVTAYSHATSAQEEYEKFKRINV
IQPKVMPHRVPSLYQDLLPNRHVPLALPLAMPYHFQGQATSTGQSDPTSVQEQPVDFSPK
NNFTHSAKTSPFELTGNYAMVA
>Drosophila melanogaster snail
MAANYKSCPLKKRPIVFVEERLPQTEALALTKDSQFAQDQPQDLSLKRGRDEETQDYQQP
EPKRDYVLNLSKTPERNSSSSSNSCLLSPPVEAQDYLPTEIHMRGLTAGTTGYTTATPTT
INPFQSAFVMAAGCNPISALWSSYQPHLAAFPSPASSMASPQSVYSYQQMTPPSSPGSDL
ETGSEPEDLSVRNDIPLPALFHLFDEAKSSSSGASVSSSSGYSYTPAMSASSASVAANHA
KNYRFKCDECQKMYSTSMGLSKHRQFHCPAAECNQEKKTHSCEECGKLYTTIGALKMHIR
THTLPCKCPICGKAFSRPWLLQGHIRTHTGEKPFQCPDCPRSFADRSNLRAHQQTHVDVK
KYACQVCHKSFSRMSLLNKHSSSNCTITIA
>Drosophila melanogaster worniu
MDKLKYSRCPLKKRPIMVEESSPEDHLSHDEGPVDLSVASAAVPMEPHWMAKSEPEPQPV
PTELRRRFDAAMNQTKEQLARRIWEETREIARAFPDVFTREEIAKSLARLGYGEFELPPE
EEVMEPEPEPEPEQHLPLRYTRDASPTIIKAEPSDEEQFPLRNYNNNLLKSIAEYEDCMKMQ
NIKEEIPPIPSPQLFYPPPTPLAEPEDLSVTQRRVLSENMNLQNVARALLSMQHMAPQHA
PPPIDMEEDQENQDINQLKIKSSNDLYYQCQQCNKCYATYAGLVKHQQTHAYESTEYKII
RSQPGGSGAIVDQTEFCTDQASALIQAANVASAQSMQKPVGVPRYHCQDCGKSYSTYSGL
SKHQQFHCPSAEGNQVKKVFSCKNCDKTYVSLGALKMHIRTHTLPCKCPICGKAFSRPWL

LQGHIRTHTGEKPFSCQHCNRAFADRSNLRAHMQTHSDVKKYSCPTCTKSFSRMSLLAKH
LQSGCQTEQSGGPSGSGGGFDQQQLQQHLQVYEEGHNPHQLYYAGSVGSSNGEEEEGGEY
QMQPPAIY
>Drosophila pseudoobscura CG15269
MGFKHAMLQRSYEMSPSPARTSPTNANSTTSSSTNSTTNECPPAVAQAPAQVTDFSISRI
LGKERRSSPTDATANILDLSKSGSISTSHSSPPQGAPQVPPTIAPSPPYMGYSMLPPDLV
AMANATKFYAQFFPHLLPAYAAAAAAAGLSTPPTSPYQQQQQLQQHHHQQQKRYFAPYVI
NGQAPQQQQHPNLIRPKATATACTRPDCLECLEYYQKLQAGYKSTPLISPAASSVSSSSS
SAMRPVRDLVNSTNISLTTVTNFSMNTAQTAAGLMPKMDGLLPNAVGMGSNISSSRSSSI
NPINPYNPTAAEEISYKCRICEKVFGCSETLQAHEKTHKSPRYECSDCGKGFSQLRNYKY
HLSVHRGTKEFAAECPECGKTFNDKGYLSSHMKIHRNRKEYECPYCPKSFNQRVAFNMHV
RIHTGVKPHKCSECGKRFSRKMLLKQHMRTHSGEKPYQCSVCGKSFADRSNMTLHHRLHS
GIKPFSCPLCPKAFTKKHHLKTHLNYHTGCKPYVCPHPNCNQAFTQSSNMRTHAKKCQYR
PLDSTPSSYPVPGKAQPQGQMQMQVPTSLALAMASAPTFAMPPPPPPAQQSLLSNLRTY
>Drosophila pseudoobscura escargot
MHTVEDMLVEKNYSKCPLKKRPVNYQFEASAQTPVNEPQDLCLKKTESEELEVEADPATV
SASEEVINVSDCCDDEGVDVDHTDDEHMDLEEEDDVTVDCVDVDTDPNQTQVVVPTPTYP
KYGLQPWNFHMSPYTAEFYRTINQPHLAAVAASAAQQQISPLRGDLLAPSSPSDSLGSLS
PPPHHYLHGRASSVSPPMRSEIIHRPIGVRQQHRFLPYPPLPAAAYHQQTPYPALGYHHH
APISPAYSESSYYSMRSMTPESSCSSVPEDLSLKHKPIGQEAASSIKAGAGPSTSGSASS
SASPSPAKKDKSPPRYQCPDCQKSYSTFSGLTKHQQFHCPAAEGNQVKKSFSCKDCDKTY
VSLGALKMHIRTHTLPCKCNMCGKAFSRPWLLQGHIRTHTGEKPFSCQHCHRAFADRSNL
RAHLQTHSDIKKYSCSSCSKTFSRMSLLTKHSEGGCPGGSSPSSNAGSTSELSYAGYAEP

>Drosophila pseudoobscura scratch 1
MPRCLIAKKWKAYPWLDRSEDNTQQPQQQQQQQHQQQQNATNTSTTSRDEDLSHLKSRR
STLDDDEEIDVVGDKFLTKLEKQRTTIASVAATAAGATAAEATNNSNSSNSSNSNNSNES
TTATVTATTTTSAKCWGPSSPTAGTTAPSPPPHSPEAATRVASNVYNGYTRELSPLHYTA
YLPRMESEITVIRAAATALVAAASGNGSSPAGDQHLASYQTPPSSTTSSPSCSPSGAGDR
MVDRYSPHIKMETGQTSSERKCFSSTGATLSLPPKKKDIYRPYSLDDKPTHGYRKRPAEE
DLHAAHAILDLSASTAFHPPTQPHQLQQQQQQQQQQHQQHQQHHQQHSHHLPLQQQQQQ
QHALVHNHSHSASFEAHAHLRSTSSIAELAAAASAVNEQRPSSNASSASSQHAMPSSPAS
SSHGSILSCSGSGSGSSVQNENSNTTNTNQDCDGGSVDGEAGGTQAAKTVAYTYEAFFVS
DGRSKRKHVADPAAVVVQDQQKTKYTCSECGKQYATSSNLSRHKQTHRSLDSQSGKKCHT
CGKAYVSMPAPAMHVLIHKLSHSCGVCGKLFSRPWLLQGHLRSHTGEKPYGCAHCGKAFA
DRSNLRAHMQTHSVDKNFECKRCHKTFALKSYLNKHLESACLKDEEELMMAMSLHGHGGD
SNSESGASMASSPPHEFLERVKVECPNGGAGSGLGGNTYAM
>Drosophila pseudoobscura scratch 2
MSVDFILPNRNKIQTIAYASASRKYAAAASPASTGSGSSSSELQSWRRPKPRNGSKLRLE
DRSSSSVLRNANGNVHASGNGNGNGSKSSLKSLTKRSSRSLLKREVIELDDEADREEGPA
GEEQLCEHLEWSAAQSLVQMTSSKQEKQRRLGGGSLGGASGTTGTAAAATVSASGMCATV
DHFSFSDGKVIFYAPPASACGAARQPEPMSVKRQREQQVAANASAAASATSIYRGRSAEE
TEAAHDLLSLSQSLPPLIPPCVVTIMKQEQEHLKSSAEHLPIMQEISNASNKSQSNIRFI
GSSSYDMMNGTPEASNNCSPLTPPNSDHSSDVDIDMSSSSESGQMHWGKQPHQPPQQQRA
STLKMCLNMMDGRTKASKAAAGKDREQLHQRPKSSSSNASGGAGSGVADGRAAAVAGAAA
AAGAGPSGNGENYAKRKRGCYKCSECGKQYATSSNLSRHKQTHRSLDSQSAKKCNTCGKA
YVSMPALAMHLLTHKLSHSCDICGKLFSRPWLLQGHLRSHTGEKPYACVHCGKAFADRSN
LRAHMQTHSGDKNFKCHRCNKTFALKSYLNKHLESACLRDAGAIGQGKGDDYDCDLDDDD
EMMGKLEHDQDEFDGDEMDSDETSQDIEVA
>Drosophila pseudoobscura scratch 3
MTKDQQATPIPEELYNLTQLAEVTLAAGPLITDGEVKPHFYACSSDDDSNYYSHKVFDRR
KLRRCTISDSNSCHQSGEPQEQERHDHQEQATEDEHICPECGKKYSTSSNLARHRQTHRS

IMDKKARHCPFCEKVYVSMPAYSMHVRTHSQGCECHHCGKCFSRPWLLQGHIRTHTGEKP
FKCSVCSKAFADKSNLRAHIQTHSNTKPHSCSRCGKAFALKSYLYKHEESSCMKNRSSGG
GSGSGTGRPPASPKRQQEGGSSCGSSSGSISGSPSTVTTTAPPDSAKSTLANKLLQKEKE
RRQAALAYQSSSSSYPLADNEHFKRTNVIQPTALAHPSVLYQTMHPLHYHYHVPNPPPPT
EGGAAGTGAQEQPVDFSPKNNFTHSAKTSPFELTGNYAMVA
>Drosophila pseudoobscura snail
MAANYKSCPLKKRPFVFVEEHPQTEALALTKNSSFAALPAGEDQPQDLSLKRKASREQDF
EDYELPAKREYVLNLSKTPETPRSASPLCSALLSPIAEHSDYQPESESQAQCQPIDIHMR
GLTAATAGYTTNPYQSAFVMAAGCNPISALWSSYQPHIASHLSAFPSPASSMASSMASPH
SVYSYQQMTPPSSPGSEASSEPEDLSVRNDIPLPALFHLFDEARSSSASSSSGSVGSYAY
LAASSAPNASGAVGSASSAAKNYRFKCDQCQKMYSTSIGLSKHRQFHCPAAECNQEKKTH
SCEECGKLYTTIGALKMHIRTHTLPCKCPICGKAFSRPWLLQGHIRTHTGEKPFQCPDCP
RSFADRSNLRAHQQTHVDVKKYACQVCHKSFSRMSLLNKHSASNCTITIA
>Drosophila pseudoobscura worniu
MDKLKYSKCPLKKRPIPIEDAFPVDQDQDQLSHDEGPVDLSVASAAVPLEPAVPTELRRR
FDAAMTQTKEQLARRIWEETREIARAFPDVFTREEIARSLARLGYGDFELPPEEEVMSPE
PEPEPQPGLPVSYAGVSPPPTAIPQTIIKVEQPEEDFPLRNYNNNLLKSIADYEDCMKMP
QPQMQPHPLSYYQPPPTPGLPEDLSQRRVLSENMNLHNVARALLSMQHMPPQQPRYEPPH
QKAAKQELPPLSGAEDTENGGENGENQLNQLKIKSSNDLYYQCQQCNKCYATYAGLVKHQ
QSHAYESTEYKIIRSNPSGGPIVDQTEFCTDQASALIQAANAASAQSMQKPVGVPRYHCQ
DCGKSYSTYSGLSKHQQFHCPSAEGNQVKKVFSCKNCDKTYVSLGALKMHIRTHTLPCKC
PICGKAFSRPWLLQGHIRTHTGEKPFSCQHCNRAFADRSNLRAHMQTHSDVKKYSCPTCT
KSFSRMSLLAKHLQGGCHSDAVGGATSGGPGFNQEQLQQHLHGYEEGHQVYYAGSMGSSG
AEEEEAYALAPQA
>Halocynthia roretzi snail
MDMCFISYEEKKKDIIDTNDSSTLYDDSCSSSNDECSAPTSVTSPSSCSAGSSKYTDNNN
RTSDSSLTTSHDFGTSDDIADDLQPIDLSLKKQNDRIRFCNDNRTMQLPQRTNENYNDFL
MQKMDSFPMLNFPIPSSMRGYSPWHHYMCASPLSFIPPSSLPNWNPVSSIGEMIMPMNPL
LYGLPMGEVSSASVAKPSYYETSQHLWKSLATAGRADASLFRKFVATHEDAYAPKKGRAA
PMFPNQELYQVWNQHLKRTDDRKSRTDLEKELVDDQVIPQKISARVKKERDINVHQYFKN
NQECTSAGRTEEQSRSTDQQRYVIDREEGLIKTKDKNLLENKSPNKRRNEDPSTSDEHYG
TSAKKIKMQASEDTNKELVHASASDNTDDSNQTNGTSKNIGGNLQMTPKPINFKPCRFHC
SECGRTYATVGGLMKHSKYHHDPENAMTFKCKTCEKEYTSLGALKMHIRTHTLPCKCHIC
GKAFSRTWLLQGHIRTHTGEKPYQCSVCLRAFADRSNLRAHMQTHQNVKRYACTGCEKTF
SRTSLLNRHRASGCVP
>Homo sapiens snail 1like
MPRSFLVRKLPDPDRKPNYSKLPDSNPEFTFQQQPYHQAHLNSTALLPTLIWDAFLALQA
QPAARASLQLRLPQESFKAAELTSLSDEDSGKGSQSPSPRSQPSSSFSSTSASSLEAEAD
TTFPGLGQVCKQLAQVSEAKDSQFQKAFNCQYCNKECLSLGALKKHIRSHTLPFACETCG
KAFSRPWLLRGHVRTHTGEKPFSCTHCSRAFADRSNLRAHLQTHLDVKKYQCQACARTFS
RMALLHKHQESGCSGGPR
>Homo sapiens scratch 1
MPRSFLVKKVKLDAFSSADLESAYGRARSDLGAPLHDKGYLSDYVGPSSVYDGDAEAALL
KGPSPEPMYAAAVRGELGPAAAGSAPPPTPRPELATAAGGYINGDAAVSEGYAADAFFIT
DGRSRRKASNAGSAAAPSTASAAAPDGDAGGGGGAGGRSLGSGPGGRGGTRAGAGTEARA
GPGAAGAGGRHACGECGKTYATSSNLSRHKQTHRSLDSQLARRCPTCGKVYVSMPAMAMH
LLTHDLRHKCGVCGKAFSRPWLLQGHMRSHTGEKPFGCAHCGKAFADRSNLRAHMQTHSA
FKHFQCKRCKKSFALKSYLNKHYESACFKGGAGGPAAPAPPQLSPVQA
>Homo sapiens scratch 2
MPRSFLVKKIKGDGFQCSGVPAPTYHPLETAYVLPGARGPPGDNGYAPHRLPPSSYDADQ
KPGLELAPAEPAYPPAAPEEYSDPESPQSSLSARYFRGEAAVTDSYSMDAFFISDGRSRR
RRGGGGGDAGGSGDAGGAGGRAGRAGAQAGGGHRHACAECGKTYATSSNLSRHKQTHRSL

DSQLARKCPTCGKAYVSMPALAMHLLTHNLRHKCGVCGKAFSRPWLLQGHMRSHTGEKPF
GCAHCGKAFADRSNLRAHMQTHSAFKHYRCRQCDKSFALKSYLHKHCEAACAKAAEPPPP
TPAGPAS
>Homo sapiens snail 1
MPRSFLVRKPSDPNRKPNYSELQDSNPEFTFQQPYDQAHLLAAIPPPEILNPTASLPMLI
WDSVLAPQAQPIAWASLRLQESPRVAELTSLSDEDSGKGSQPPSPPSPAPSSFSSTSVSS
LEAEAYAAFPGLGQVPKQLAQLSEAKDLQARKAFNCKYCNKEYLSLGALKMHIRSHTLPC
VCGTCGKAFSRPWLLQGHVRTHTGEKPFSCPHCSRAFADRSNLRAHLQTHSDVKKYQCQA
CARTFSRMSLLHKHQESGCSGCPR
>Homo sapiens snail 2
MPRSFLVKKHFNASKKPNYSELDTHTVIISPYLYESYSMPVIPQPEILSSGAYSPITVWT
TAAPFHAQLPNGLSPLSGYSSSLGRVSPPPPSDTSSKDHSGSESPISDEEERLQSKLSDP
HAIEAEKFQCNLCNKTYSTFSGLAKHKQLHCDAQSRKSFSCKYCDKEYVSLGALKMHIRT
HTLPCVCKICGKAFSRPWLLQGHIRTHTGEKPFSCPHCNRAFADRSNLRAHLQTHSDVKK
YQCKNCSKTFSRMSLLHKHEESGCCVAH
>Homo sapiens snail 3
MPRSFLVKTHSSHRVPNYRRLETQREINGACSACGGLVVPLLPRDKEAPSVPGDLPQPWD
RSSAVACISLPLLPRIEEALGASGLDALEVSEVDPRASRAAIVPLKDSLNHLNLPPLLVL
PTRWSPTLGPDRHGAPEKLLGAERMPRAPGGFECFHCHKPYHTLAGLARHRQLHCHLQVG
RVFTCKYCDKEYTSLGALKMHIRTHTLPCTCKICGKAFSRPWLLQGHVRTHTGEKPYACS
HCSRAFADRSNLRAHLQTHSDAKKYRCRRCTKTFSRMSLLARHEESGCCPGP
>Lottia gigantea scratch 1
MPRIFMITNRRYFSNIVEEKEDRFAPFQGKIHCVSSQLLNAVTVNSNTEACRKEVKFMTI
GCFQTHELTMFIVISEKAEKKIVSDDGDSDSGCSTQTETRTDLSPEIEYGWGCHSNLTLL
YDLMIQSPRKAVCQDVPEQAIDYSMSGQNSPNPPSPPSPSTASPQYLARRSLNELLLHSP
TQPHDHQDDDPRDCLDCVKYSSRSSPSHHRPISRTANDKKARKCPHCDKVYVSMPAYSMH
VRTHNQGCECVYCGKKFSRPWLLQGHIRTHTGEKPFSCPKCGKAFADKSNLRAHIQTHST
EKPYICGRCGKAFALKSYLYKHEESSCMRGQRFRH
>Lottia gigantea scratch 2
MPKSFLVKKSKYGTDENKNGKSFRNNLEDNRSDISIQAAYPVNVDSEAIFIQLNNGEYCV
KYHLFTQHLTSFIISADRFCNIFSITACLSLFYFLLATVKSIIVYCSFFYKVPKNNTYSP
FSGYMQSVPAAFEHSQKEDLIGEGESMTSPESSKTAEYSCYPSPVLLESNSQYQPNHYGG
FIVVDGRTKHRQNGSPRQRFRCDECGKDYATSSNLSRHKQTHRSLDSQLAKKCPHCEKVY
VSMPALSMHILTHSQRHICNICGKTFSRPWLLRGHIRSHSGQKPFGCSHCGKAFADRSNL
RAHMNTHSAFKQFQCQYCKKTFALKSYLNKHHESMCLK
>Lottia gigantea snail 1
MPRAFLIKKRDRGIMEGTAVTEESVSETNNNTIINVVDVCDDEEETINVTDTDDVIMEQS
ENIYKDMINNQKVIVEEIPRHISPNMIPESHKNTIREPTVIKPTVVKPIAERPQPHHIPV
SVPLPSPPDCHRRLSPHTPTDFDRRTSSLSPVDGPSCSPPEPARFHDPFPWQPALIPNIH
SHPFLPFRFMNGFPSPPLPQPAQDSPTGFRPYSELSAFKGPQLLPPPPPYLPGMRYPMNF
SPPRHDRIIDERRMDFEFGQKLMKSPFDNVLNYPVGAERVLSQLDLPEKKKSKENEPIRY
QCDSCKKSYSTFSGLSKHKQFHCASQIKKEFNCKYCDKTYVSLGALKMHIRTHTLPCKCK
LCGKAFSRPWLLQGHIRTHTGEKPFSCQHCGRAFADRSNLRAHLQTHSDIKKYSCRGCSK
TFSRMSLLLKHEDSCCGPVPH
>Lottia gigantea snail 2
MGVFVVYVLEQNVTSNTNVPDGHPMMSQSDILRHFHQSNDTQSITNNTSHSSIMREQTNK
STGQFLASSLEMSVSERQAVRTDNTEVKSDPKSAVKPIVRPWLIEPSTKKSNQKQNNPIV
SQSQNVPNVVQPTHPVVPLPLSPRYCSDQLTLNQIDAIRLLRDQAVLYSDPKTSYYHDPS
FRNNLNSWFYFLRNGSCEGRVTTDQWPNTAISPSHHSMSINGQSPPRFQCDACQKSYSTF
GGLSKHKQFHCAQQIKKDFRCKYCDKSYSSLGALKMHIRTHTLPCKCKLCGKAFSRPWLL
QGHIRTHTGEKPFSCQHCGRAFADRSNLRAHLQTHAEIKKYGCKNCSKTFSRMSLLVKHG
ESSCMGIVR

```
>Lytechinus variegatus snail
MPRSFLVKKNAKQAASGLKKLQHGIATTAYDPGVVSVASFPEMSVAAPINIPHPVIHKPE
PLQAIPNPSAYWRHHQNVIYSPSSPFEADTFPHIHKGYSPPPPFSQPLHAHLPQPSLHHL
LTPTPIIDDENRMITSLSPNQHRHHPHHLSQHRQQHLQASANIPVTPTGTATAAKPEKEI
KKRRSNKNGEDSTKYHCPDCGKEYSTFGGLSKHRQLHCDAQNKKTFNCKYCDKEYMSLGA
LKMHIRTHTLPCKCKFCGKAFSRPWLLQGHIRTHTGEKPFSCPHCQRAFADRSNLRAHLQ
THSEVKKYSCKSCGKTFSRMSLLNKHEESGCISSGSD
>Mus musculus scratch 1
MPRSFLVKKVKLDTFSSADLDSSYGRARSDLGVRLQDKGYLSDYVGPASVYDGDAEAALL
KGPSPEPMYAAAVRGELGPAASGSAPPPTPRPELATAAGGYINGDAAVSEGYAADAFFIT
DGRSRRKAANANAAAAPSTASVAAPDSDAGGGGGPGTRGSGSGSASRGGTRVGAGTEARA
GSGATGAGGRHACGECGKTYATSSNLSRHKQTHRSLDSQLARRCPTCGKVYVSMPAMAMH
LLTHDLRHKCGVCGKAFSRPWLLQGHMRSHTGEKPFGCAHCGKAFADRSNLRAHMQTHSA
FKHFQCKRCKKSFALKSYLNKHYESACFKGGASGPATPAPPQLSPVQA
>Mus musculus scratch 2
MMKKESRSSSLLDIVNSWDLACVPYKAGSRSAEHSYSDRLCARRRRRRRRRRRARAAPEP
EPRPSPPRRLRGRPPAGSCPVLKPAPTANRAGRPPPPPGLPCPASPPPGCTMPRSFLVKK
IKADGFQCSGVSAPTYHPLETAYVLPGTRGPPGDNGEWALREGSGGSPTWAAAGNRGGDV
RGREHPRDFQRTGRAGLGLGRTKESGRVGSSRSHAPIGTLKVVALEYGWSYVAHCLPPSG
YDGEQKPGLELAPAEPAYPAAASEEYSDPESPQSSLSARYFRGEAAVTDSYSMDAFFISD
GRSRRRRAGAGGDAAGAGDAGGGGGGGGGGERAGRSGATAGGGHRHACAECGKTYATSS
NLSRHKQTHRSLDSQLARKCPTCGKAYVSMPALAMHVLTHNLRHKCGVCGKAFSRPWLLQ
GHMRSHTGEKPFGCAHCGKAFADRSNLRAHMQTHSAFKHYRCRQCDKSFALKSYLHKHCE
AACVKAAEPPPSAGPAS
>Mus musculus snail 1
MPRSFLVRKPSDPRRKPNYSELQDACVEFTFQQPYDQAHLLAAIPPPEVLNPAASLPTLI
WDSLLVPQVRPVAWATLPLRESPKAVELTSLSDEDSGKSSQPPSPPSPAPSSFSSTSASS
LEAEAFIAFPGLGQLPKQLARLSVAKDPQSRKIFNCKYCNKEYLSLGALKMHIRSHTLPC
VCTTCGKAFSRPWLLQGHVRTHTGEKPFSCSHCNRAFADRSNLRAHLQTHSDVKRYQCQA
CARTFSRMSLLHKHQESGCSGGPR
>Mus musculus snail 2
MPRSFLVKKHFNASKKPNYSELDTHTVIISPYLYESYPIPVIPKPEILTSGAYSPITVWT
SSAAPLHSPLPSGLSPLTGYSSSLGRVSPPPSSDTSSKDHSGSESPISDEEERLQPKLSD
PHAIEAEKFQCNLCNKTYSTFSGLAKHKQLHCDAQSRKSFSCKYCDKEYVSLGALKMHIR
THTLPCVCKICGKAFSRPWLLQGHIRTHTGEKPFSCPHCNRAFADRSNLRAHLQTHSDVK
KYQCKNCSKTFSRMSLLHKHEESGCCVAH
>Mus musculus snail 3
MPRSFLVKTHSSHRVPNYGKLETLREANGSCSACKELAGSRHLPDEEAPCNPSDPLQPWD
STSAVACISLPLLPNHRETLGVSGPEPQETSWVGPRAAQAPSVTLKDSFTLPPLLVLPTR
WPPILGPDGALNEHLRAEGTSRVPGSFECIHCHRPYHTLAGLARHQQLHCHLPTGRAFTC
RYCDKEYASLGALKMHIRTHTLPCICKVCGKAFSRPWLLQGHIRTHTGEKPYTCSHCSRA
FADRSNLRAHLQTHVGTKKYRCAVCPKAFSRMSLLARHEEAGCCPGP
>Nasonia vitripennis CG15269
MPVDVTIPSQQADLYRGSVVSPCTASGCCSLSPQPLISKEPALPPAVVHPSGFYQVPNPR
PTSADYYARAPSLPPPPPFPEPPPSLPAVVPPPPRCVPFVPEQKLPVVQSKKPELSLPVS
RLCDSLESKDEARTLPVKPEPRIRPSVLPVLAHQEKSLPHGFALVSDDNNNNSSIGPEQQ
RSRGSSATAQTNEEHKCGQCSKTFVTRASLKVHLRTHSGEKPFRCLDCGKQFSQLRNYKY
HRSVHEGTREFAASCPECGKYFNDRGYLSSHMKIHRNRKEYACAECGKSFNQRVAYNMHV
RIHTGLKPHQCEQCGKAFSRKMLLKQHLRTHSGERPYQCQVCHKAFADRSNMTLHTRLHS
GLKPYQCTLCSKAFTKKHHLKTHLNYHTGTKPYSCPNCSLRFSQSSNMRTHYKKCALNNP
NGVAAAAVAAAAAAAGATDDKQPAKQSQQPAGSAVQPPALTPPNSDQESSNNTVQQPQQQ
QIVK V
```

>Nasonia vitripennis scratch 2
MPRAFLITRRRYNGTGEFEEIGREYSPERGVRLAEYGGGQQPTSDGASECGSESSSECPE
ELYNLTKLAEVSLAAAAGTLVHPATNVYHQRPAPLPASQQRLFFERMEAENQQLHELKSC
RRAGERQRFPAPFNQVRLTVFDVLEHDDTSIEYSRIGSNRQNGVQHMTTMDIVATPAQAQ
NPMLEGQHQNSGQQAQDGAEEHECPDCGKKYSTSSNLARHRQTHRSLGDKKARRCPHCDK
VYVSIPAFSMHVRTHNQGCKCHFCGKCFSRPWLLQGHIRTHTGEKPFKCTICNKAFADKS
NLRAHIQTHSNTKPHVCGRCGKAFALKSYLYKHEESSCMPRLEHNKALCLLALN
>Nasonia vitripennis scratch
MPRCLMAKKWKAYPWPDRAEEQHQMEVEQQPQPQTDDEEIDVVGDVDSGNSSGSEKSEAA
QQTCWGPHSPTAGATAPSPPPLNASGALYYHGYTHEAWINHEEPPKYATLRSAAELARPM
VPSPTPSHHHQHQIHEHQQEPHPESSEHSLPQQQHQGAALPPRKSMAMCFTSTGTALSL
PPKKKDIYRPYSLQPCEIRTSAEEDLSAAQAILDLSASPAPQPQPIPVSVLQQQQQQQQ
QQQQQQPVAVSTSQPEPQTAQPQSPATAEVPGGQQSNKTVAYTYEAFFVSDGRSKRRSSN
AAVPEKEAAGQPDRPKFTCTECGKQYATSSNLSRHKQTHRSLDSQSAKKCIHCGKAYVSM
PALAMHVLTHKLAHSCGVCGKMFSRPWLLQGHLRSHTGEKPYGCAHCGKAFADRSNLRAH
MQTHSADKNYECSRCHKTFALKSYLNKHLESACLRDDEQGSTGAPAVGQPPPHQLPSANN
NRGL
>Nasonia vitripennis snail
MLVDEMPRSFIKKNNSYSHCPLKKRPVNVVDDVSEPENLSTKPEDLSRSAPKRSRSPSPI
AVSSPVHYPVLKRSPSPICASTRSPSPVAQHPIAHIPSYQPQQHHYPTKAMTPPAAIAPV
HPVAKKARVEVLQHDNASASAAIGSPMHFMAASKSPLEPLNLNSPVEPLAHYASPAWARA
AHAAPLYPPHYLPYPAAYRYHHAAAELYPAYPVAAYPHSSPEHQSVSPSPPHAALTCQSI
QRPLARSYASHWSSPDHCGLSPTSSLGSSAMRSPPPVTPEDLSSPGSDSGRSSAGSTSAP
GSMIVKLDKSPGASSSSSSNGSSSPRYQCPDCAKSYSTYSGLSKHQQFHCAAAEGQTKKT
FTCKYCEKVYLTLGALKMHIRTHTLPCKCHLCGKAFSRPWLLQGHIRTHTGEKPFSCQHC
NRAFADRSNLRAHLQTHSDVKKYSCSSCSKTFSRMSLLTKHQEGGCPGGSRGSLNSRERD
SRKLVFEIPD
>Nematostella vectensis scratch
MPKSFLIKKKSKEYNNNTSTAAGTGKSKKIKLDESGKMIFFDYDSSFKHLLYELNPALSC
CLDTTEDNLISHIENPSKLHSNGRISESTSECEDYNEGKSEENGEDGEVDENGNLVASVK
SVCADGRSKGNGGNGGKRSRYVCAECGKSYATSSNLSRHKQTHRSLDGKLAKRCHQCGKA
YVSMPALAMHVLTHKLLHKCEICGKSFSRPWLLQGHKRSHTGERPYSCPECNKAFADRSN
LRAHLQTHNPLKQYKCERCDRTFALKSYLNKHLESACSRDSSEKNEDSP
>Nematostella vectensis snail 1
MPRSFLVKTKTERCSHFDSPIIPSRPDNGCIRDISRVDDVAGNVKDGKQETLQKNNTNRS
NCTAVHAQVIPLGIEETHRTQNTYPVKEDIKSSPTSKTLVACTGVPSHDPSRVVTSEAPA
KLQCPNCMKGFNALATLMRHQYFYCPPTQHKRPFHCKYCEKLYDSLGALKMHIRTHTLPC
KCKICGKAFSRPWLLQGHVRTHTGEKPYKCTQCQRAFADRSNLRAHLQTHSDVKKYSCKQ
CSKSFSRMSLLLKHEGSCSGASSAAMCRQNLC
>Nematostella vectensis snail 2
MPRSFLVKKTCDKKALLLRNGPVMTREEGGKSVESHRKGSSPERVENEDKADKRLQYWNG
LGTSLVYPAEVTSVPGLHNKPTEFCIDVKYERSNTKDDVDMGLSRELEYGTKTASLTAEE
SRPKHQCHQCNKGYSTPLGLAKHQQFHCNTHHKKSFTCKHCDKIYVSLGALKMHIRTHTL
PCKCSICGKAFSRPWLLQGHIRTHTGEKPYQCTNCKRAFADRSNLRAHMQTHAVVKKYSC
SRCKKSFSRMSLLVEHEDSGCPSQG
>Patella vulgata snail 1
MEKNPDPSEKEEVEYRLSSMEYIEIDVTDDTSVFDGHKLMTQSDILRHAHLVDNSPRHTR
FSHVNEVVVERSNMMEDMLKTNKLTDQFVTLSHETPVSEKMNSQCGNTDQKEAPKSAVKP
IVRPWLIEPSVKGRNQTPKTVFTPTTVKARIETKLPQNTTLIPQPVLPYCANPTATQQLD
AIRLLRDRNPMFANPNPYRLFHDSSFRNNLNSWFSWLRNGMSEPQNMNHWNGQKLPDVSE
PVQLNAHSPPRYQCEACQKSYSTFGGLSKHRQFHCSQQVKKEFRCKYCDKSYSSLGALKM
HIRTHTLPCKCKLCGKAFSRPWLLQGHIRTHTGEKPFSCQHCGRAFADRSNLRAHLQTHA

EIKKYGCKSCSKTFSRMSLLLKHGESSCMGMVR
>Patella vulgata snail 2
MPRAFLIKKRDKSESETQIFNPRSDNPPAAADVEEDEETINVTDTDDTPMEVTESNLKSN
EATLSPKVLVEEMPKPVVTPVPRLSPIQQKRDMYLTGRSMEGIIKQTIVKPIPERPMIPV
SLHLPPTPDCHRRLSPHSPADLDRRTGSLSPVDSPSCSPPEPARFHDPFPWQPPLISNLH
SHPFLPFRFFSGFPSPPLPRNLNDSTLGFRPYAEVSSAFKGPQLLPPPTYLPAGMRYPMN
FPQSPNDTRKLFDDRRMDLDFSVKMAKAPFDNVLNYSVVTERPVPELELTEKKKSKENEP
IRYQCDSCKKSYSTFSGLSKHKQFHCASQIKKEFNCKYCDKTYVSLGALKMHIRTHTLPC
KCKLCGKAFSRPWLLQGHIRTHTGEKPFSCQHCGRAFADRSNLRAHLQTHSDVKKYSCRS
CSKTFSRMSLLLKHEDGCCGTVVH
>Platynereis dumerilii snail 1
MPKSFLIKKHGATPVTPCGIKRSDVMTSQEDQTSSRDHMATESVMKPSSQLPPCSPTLPM
SPISRQDEVHETGLPAAHSPGASSPHNAGASPVQQRTPSPFSISAILSSPGSKSPPSRPL
AFVPQPFYPVYAPFFNAADKGMMSPPLPKQQHSPVHHQVHQVHHQVHQVHHQVQSQGHNQ
VQPAHHVAQPVSHSHHQQVPVSPSHHHPVQPVHHQVPVSPSHHHHHHQGQCGSHHHHQVQ
PQCQEAMDYTVTTTPIKKDTPKDFDPAVVKKDTPKDFDPAVVVSEGPSNPAKGPPRFNCE
GCGKTYATFSGLSKHKRFHCVSEIKKEFSCKFCDKTYSSLGALKMHIRTHTLPCKCPLCG
KAFSRPWCLQGHIRTHTGEKPFNCNHCSRAFADRSNLRAHLQTHTDIKKYSFKSCSKTFS
RMSLLIKHEDGGCPGMSS
>Platynereis dumerilii snail 2
IYYEHSFTIIEVIRNFNLPVSGGPPRIPLSPVQEASSPQIIDPRFTSQWTEKNLTAAVDI
LRSVTSSTPRAHHHGNLENRRPKSPRMSPYNVPSKHYFTNITRIASERSHNYVSIIKGQE
VKSSAERSLSAFNEINCEAKRSTVLQPLNVSTNHIASPPQSHKRPEVHVTSQHYTVLTPS
RPSVNHLQELQQLKEMKPVQRSLQMQHSPQWDAENRPAAIFQDGYQDGFQDGRHENVRRD
ISRFKCEDCSKSYATFSGLTKHKQFHCSALFKKEFSCKYCSKTYVSLGALKMHVRTHTLP
CKCPLCGKAFSRPWLLQGHIRTHTGEKPFNCNHCSRAFADRSNLRAHLQTHTDIKKYNCK
NCPKTFSRMSLLLNLKMEDALHIYGIPKKNLIHLPIPKIPKNFLGILFKKKMAGWNFLIH
P
>Saccoglossus kowalevskii scratch
MPRSFLVKITKSEDASSHVESRTQTQPLAMSNRYLNSISVRLNNGYIHDLIPVSDIQNNN
DVSDLSIKGESDDKIEEFKKNNFNGEQTVVAYDALLITDGRSRKGLRAALVGSDEQNRQR
YKCNECGKHYATSSNLSRHKQTHRSLDSKLAKKCPTCDKVYVSMPALAMHVLTHNLKHRC
SICTKSFSRPWLLQGHMRSHTGEKPFGCAHCGKAFADRSNLRAHMQTHSAYKNYECKRCN
KSFALKSYLNKHYESACFKGMQPPLSPSSSTSSLHGESGPFSPL
>Saccoglossus kowalevskii snail
MPRSFLIKKHCNHSHRKQSGILRTVFKAYYDVSPPVVLKPEPVHITPNEYWRWTENLPIT
PVMPQSHHDSYDLKLHDMTPPPLPGTLPVMIQGMTLENHHHHHHFHPSPPPPPPPLSSAS
PTSAAIENLKLGKGKRSSNGEHTRYQCTECSKSYSTFSGLTKHKQFHCVTQAKKSFSCKY
CEKEYLSLGALKMHIRTHTLPCKCKICGKAFSRPWLLQGHIRTHTGENPFACTYCNRAFA
DRSNLRAHLQTHSDVKKYCCKSCSKTFSRMSLLTKHEEAGCCSVTSI
>Strongylocentrotus purpuratus scratch 1
MPRSFLVKKTKFTSCSALCPRELDPYYPASPGSPTLPHHGVSVTINNDYIHDITPSPSSP
PSEPLMITNLLTHHHEEPPSLPSSPEPLTDSKSRDFDVDSGILSVQSKGQLFLSYEAFLI
TDGRSRRRALQNGGSGGIIGPGIDQQHSPLDGSLAAVANRPKYKCNECGKQYATSSNLSR
HKQTHRSLDSHLAKKCEVCNKVYVSMPALAMHVLTHNLKHKCNVCHKSFSRPWLLQGHMR
SHTGEKPFGCAHCGKAFADRSNLRAHMQTHSAYKNYKCKRCDKSFALKSYLNKHYESACF
KLDGEITDDMMTCTPSPMSDAENISVDGNIST
>Strongylocentrotus purpuratus scratch 2
MPRSFLVRRVSRPNKEKAAMWDCFAAETAIQSARQRRGRPRSEVGVEKKKQRAQQVTTLN
IPSTEGLLKTMDSSHSGLFILSTLAEKLDNSDKQYYDQRRALYYPNDVSGLLNQSPIRQA
NMTLNYGSIGGNAQPAEGSVPSRDDRATTPSSPIKATSPSTMMPGHSCPECRKSYSTSSN
LARHRQTHRSVTDQKARKCPHCEKVYVSMPALSMHIRTHKLGCKCHICGKCFSRPWLLQG

HIRTHTGERPFSCPQCGKAFADKSNLRAHVQTHSTIKPYVCQKCNKAFALKSYLYKHVES
ACYRAT
>Strongylocentrotus purpuratus snail
MPRSFLVKKNAKQAASGLKKLQHGIATTAYDAGIMLVASYPEMAVAAPVNIPHPVIHKPE
PLQAIPNPSAYWRHHPNVIYSAATSPFEADTFPHIHKGYSPPPPFSQPLHAHLPQPSLHH
LLTPTPIIDDENRMITSLSSNQHPYHHHHHYHPYSHHRQQHLQASTPAAATPTATAAAKP
EKEIKKRRSNKNGEDSTKYHCPDCGKEYSTFGGLSKHRQLHCDAQNKKTFNCKYCDKEYM
SLGALKMHIRTHTLPCKCKFCGKAFSRPWLLQGHIRTHTGEKPFSCPHCQRAFADRSNLR
AHLQTHSEVKKYSCKSCGKTFSRMSLLNKHEESGCISSSSD
>Tribolium castaneum CG15269
MDFLQDLLLQDMTEPSVLIFPDINSNTMSLILDYIYTGSVIIYSNTINDFLTAANLLKLH
IDVEFLQKANTLREYKLFEKKEEKPANDVFSKRNQRKLPSLVPISSFMPSKKERKLVSCV
IPSPWMPREMPVLSDPRTCLSSTERANVHQIRDRLEESDPNNNNSQQHQKNKTNSILSPS
SNSSSELDLSTSLNKLAFEQKLVPQLNDSLVVNKENDKCVNDSTTAFTCPKKHTNRTLCS
RKEEGTEEKNSCHDNEKQKPFKCEDCKKTFSQLRNYKYHRSVHEGTKEFAAKCPECGKNF
NDRGYLSSHMKIHRDRKEYACPHCPKRFNQRVAYNMHLRIHTGIKPHECPTCGKSFSRKM
LLKQHQRVHTGERPYSCPECGKTFADRSNMSLHARLHTGVKPYSCNLCSKSFTKKHHLKT
HMNFHTGLKPYTCQKCGLAFSQSSNMRTHYKKCVLKSNGGPSSAEADSN
>Tribolium castaneum snail
MLDMPGTIMAKNYSHCPLKKRPVQMLEDIPDEPENLSTKPEDLSRTGRLASVSPSSLSPV
SPNSSVKTSPPPSPPMRSPSPHYYPPKTEILPYQEYAPQTWHRSVAPLYPPVYHPFVDYM
GMSPTYSESSLSPPHHHVQQPLQPLALRPYQLDQASLSPSSWTTSPPPNTTPEDLSEKRR
QGVRHQCPDCGKSYSTFSGLSKHRQFHCAAGEGPKKSFSCKFCEKVYVSLGALKMHIRTH
TLPCKCTICGKAFSRPWLLQGHIRTHTGEKPFSCTYCNRAFADRSNLRAHLQTHSDVKKY
SCPTCSKTFSRMSLLTKHAEGGCSGVHQEKMY
>Tribolium castaneum scratch
MPRCLMAKKWKAYPWPERPEQDDDHQDPEDEEIDVVGDAPACWGPSSPTAGATAPSPPPT
SPNGTTLIYNGYLHEFSPSTYPPTTFIPRNDQPPSGQTQPSYATLRTVVETQDLRPDYST
PTPPRSRKSLAMTFTSTGTALSLPPKKKDIYRPYSLDDKPPPPPPPTIRVPAEEDLHAAH
AILDLSASTTVFLPPPPPPPPPERNDEDNVLHPAPVKSGKTIAYTYEAFFVSDGRSKKRF
TPDETLTAKALVNEKPKYTCSECGKQYATSSNLSRHKQTHRSLDSQSAKKCITCGKAYVS
MPALAMHVLTHKLAHRCSVCGKQFSRPWLLQGHLRSHTGEKPYGCAHCGKAFADRSNLRA
HMQTHSADKNFECPRCHKTFALKSYLNKHLESACLKEEAGPSSCAEEVAREVPVPIRVYA
A
>Tribolium castaneum scratch 2
MPRCYMVKKQSNKYKDCWDGAAVAAAAPDSPTEACVAPPYYTPLTSSTGYSHEFIPNGAV
SSPPPASKTASGDSPIFRTRSAEETEAAHDLLSLSQSLPPLPAPGVVTIHPTVPHEDSPP
SPSPCYYRPLSPEPPLPVTVPCYPPPIVYVVQVPAAPTPPTSECSSDADFSDRTKLRSVQ
AQQDILILPLSPEPDSRLQEPPPPPPQQPAAAEQADKHETVIASSHLNIPDRRKRKNKRT
NKSNKAAKNNNDRDTLDHNVDGGKEAKAPKTKFYKVVEEESDDSADRCPTDSPDSTTTTT
TKLNRYSCGECGRQYATSSNLSRHKQTHRSLDSQSAKKCMTCGKAYVSMPALAMHLLTHK
LAHSCGVCGKQFSRPWLLQGHLRSHTGEKPYGCAHCGKAFADRSNLRAHMQTHSGDKNFS
CPQCHKTFALKSYLNKHQETACLLWDDKTKKQGSDVSTTKTLKMTTTVHAETQTITVD
>Tribolium castaneum scratch 3
MPRAFLITHRRYNLDGVEQKELNSENAVSIADEGDSQPSECPDELYNLTKLAEVAVATGQ
ILEQTRLSSSNDQPVFTYTHKLFDKSSRAPRPHLIKTEEYNRKIVSSSTVNIPHSTTQDG
VLGESNDHECNDCGKRYSTSSNLARHRQTHRSPGDKKARRCPHCDKLYVSMPAFSMHVRT
HNQGCKCQYCGKCFSRPWLLQGHIRTHTGEKPFKCTICNKAFADKSNLRAHIQTHSNTKP
HICGRCGKAFALKSYLYKHEESSCMRINGRHSSREATPDKVASPTPVIVSGPRISDSVIK
SPERPLLYRSTVISPNPERLLCSTIKHPSVILNTSARFGIGQIFQEQPMDFSANRREYGL
AIAV
>Trichoplax adhaerens scratch

```
MPKVFLIKKRKLHYPYCSVRSEKYNHDKHWGTAISTKTGDHFYFYTFTRFLSPESPIILT
FINSILLFPFPLIGSDISLDNCKVDDYGTNEKIKIACANGSKGHIEVRTGFIAKNTNRSV
ANNIDINSRQSLDYNNVVEEKNRGQAFTEKIIKPSSKQYNIESPKKHQTDFTKLSRSLKV
ANGASFPIRECVTADTFQVKNGQCNANSNDDTENNNYSHNNANSSNGKKSRYTCAECGKQ
YATSSNLSRHKQTHRSLDGQLARRCKYCDKAYVSMPALAMHVLTHELAHKCNICGKAFSR
SWLLQGHMRSHTGEKPYACATCNKRFADRSNLRAHMQTHSSVKSFQCKNCGKSFALKSYL
NKHAESGCCKNRGHRKEKGDLHELSNSSE
>Trichoplax adhaerens snail
MPRSFLVKKKANRKSRRISWPSYDSDECKLTVTKSVHALSTATVAVMYLITKFRTFVTNG
LDDEDEGDDNIEENKSMLADRYQSLSVGSRTPTPTSSEEDDNRRKGNNCIITNQYNTASI
PTNITEENISVQKDDTKDMIYHDGKNFVCKHCNKVYASLGALKMHIRTHTLPCKCKICGK
AFSRPWLLQGHIRTHTGEKPFGCEKCNRSFADRSNLRAHMQTHAEIKKYSCYKCRRTFSR
WSSLKKHENSS
>Xenopus laevis  snail 1
MPRSFLVKKHFSASKKPNYSELESQTVYISPFIYDKFPVIPQPEILSTGAYYTPLVWDTG
LLTTFFTSESDYKKSPISPSSSDDSSKPLDLTSFSSEDEGGKTSDPPSPASSATEAEKFQ
CNLCSKSYSTFAGLSKHKQLHCDSQTRKSFSCKYCEKEYVSLGALKMHIRSHTLPCVCKI
CGKAFSRPWLLQGHIRTHTGEKPFSCTHCNRAFADRSNLRAHLQTHSDVKKYQCKSCSRT
FSRMSLLHKHEETGCTVAH
>Xenopus laevis  snail 2a
MPRSFLVKKHFNSAKKPNYGELDNHTVIISPFLYERYPVSVLPQPDIYSSVAYSPITVWT
GLLHPPLPSDLSPLSGYPSSLGRVSPPPQSDTSSKDHSGSESPISDEEERLQTKLSDSHA
IEAEKFQCSLCSKTYSTFSGLAKHKQLHCDAQSRKSFSCKYCEKEYVSLGALKMHIRTHT
LPCVCKICGKAFSRPWLLQGHIRTHTGEKPFSCPHCNRAFADRSNLRAHLQTHSDVKKYQ
CKNCSKTFSRMSLLHKHEESGCCVAH
>Xenopus laevis  snail 2b
MPRSFLVKKHFNTTKKPNYGELDNHTVIISPFLYERYPVSVLPQPDIYSSVAYRPITVWT
GLLHPPLPSDLSPLSGYPSSLGRVSPPPQSDTSSKDLSGSESPISDEEERLHTKLSDSHA
IEAEKFQCSLCSKTYSTFSGLAKHKQLHCDVQSRKSFSCKYCEKEYVSLGALKMHIRTHT
LPCVCKICGKAFSRPWLLQGHIRTHTGEKPFSCPHCNRAFADRSNLRAHLQTHSDVKKYQ
CKNCSKTFSRMSLLHKHEESGCCVAH
```
