# Supplementary material for: Insights into the evolution of the snail superfamily from metazoan wide molecular phylogenies and expression data in annelids
Source: BMC Evol Biol. 2009 May 9;9:94. doi: 10.1186/1471-2148-9-94 (PMC2688512; doi:10.1186/1471-2148-9-94)
Supplement: Additional file 2 — Multiple alignment of the conserved domains of the Snail related proteins. This alignments only show the conserved domains of the Snail related proteins and has been used to construct the phylogenetic tree shown in Figure 1. [file 1471-2148-9-94-S2.pdf]

```
Acropora millepora snail                     ------------MPRSFLVKKKP----------------------------------------------------------VDSPGNLNRAKKPQKRERNASTSDVSQ-RKNKR-------------
Aedes aegypti CG15269                        ---------------------------------------------------------------------------------VHRGTKEFAAKCPECGKVFNDKGYLSSHLKIHR-------------
Aedes aegypti snail                          -TMPTSIMQ------------KNYSHCPLKKRPVFIREEE-----------------------------------------SGSSSGAPRYQCPDCGKSYSTYSGLSKHQQFHCPAAEG--------
Anolis carolinensis snail 1                  ------------MPRSFLVKKHFSAS----KKP-NYSELE-----------------------------------------SSSATEAEKFHCGQCSKSYSTFAGLSKHRQLHCD------------
Anolis carolinensis snail 2                  ------------MPRSFLVKKHFNAS----KKP-NYSELD-----------------------------------------DAHAIEAEKFQCSLCNKTYSTFSGLAKHKQLHCD------------
Anopheles gambiae scratch 1                  ------------MPRCLMAKKWKAY-------PWPDRAEE-------SKTVA-YTYEAFFVSDGRSKKKMAD---------KTALENKAKYTCTECGKQYATSSNLSRHKQTHRS------------
Anopheles gambiae scratch 2                  -----------------------------------------------SKTAELYTYDDLISSDGRSKNRKKAS--------NGTSNGKGKYKCPECGKQYATSSNLSRHKQTHRS------------
Anopheles gambiae scratch 3                  ---------------------------------------------------------------------------------TTTSRPEDVHVCPECNKRYSTSSNLARHRQTHRS------------
Anopheles gambiae snail                      -TMPTSIMQ------------KNYSHCPLKKRPVFMSKDD-----------------------------------------PAGGNGAPRYQCPDCGKSYSTYSGLSKHQQFHCPAAEG--------
Apis mellifera snail                         -------MLVEEVPRSFVKKNNNYSHCPLKKRPVQMLDEE-----------------------------------------SSSSSTSPRYQCPDCGKSYSTYSGLSKHQQFHCAAAE---------
Apis mellifera scratch 1                     ------------MPRCLMAKKWKAY-------PWPDRVDD-------SKTVA-YTYEAFFVSDGRSKRRSANT--------EAAQPDRPKFTCTECGKQYATSSNLSRHKQTHRS------------
Apis mellifera scratch 2                     ------------MPRCYMVKKALC-----------------------VKPAS-YTYDTLLVADGRSKNKKIVP--------TPETSKIGRYVCCECGKQYATSSNLSRHKQTHRS------------
Apis mellifera scratch 3                     ------------MPRAFLITHRRY---------------------------------------------------------TSKSEDNEDHECPDCGKKYSTSSNLARHRQTHRS------------
Branchiostoma floridae snail                 ------------MPRSFLIKKKLH---------------------------------------------------------SGGSGDQSRYQCPQCAKSYSTYSGLTKHKQFHCV------------
Branchiostoma floridae scratch 1             ------------MPRAFLVKRFKH---------------------------------------------------------RGGETPTGPYHCPECGKRYSTSSNLARHRQTHRD------------
Branchiostoma floridae scratch 2             ------------MPRSFLVKKVKA--------------------------------DAFYITDGRSRRKN-----------GESPARAQRYTCNECGKQYATSSNLSRHKQTHRP------------
Caenorhabditis elegans CES1                  ---------------------------------------------------------------------------------KKGPSSHNRCVCDKCGKSYATTSNLSRHKQTHRA------------
Caenorhabditis elegans K02D7.2               ---------------------------------------------------------------------------------SCSSLDNQKLVCQFCKKTYLTYFGLRRHLQFHK-------------
Capitella spI snail 1                        ------------MPRAFLIKKTER---------------------------------------------------------TDKRNGPPRYQCDACKKSYATFSGLSKHKQFHCA------------
Capitella spI snail 2                        ------------MPRAFLIKKST----------------------------------------------------------KKRDSKAGHFQCDTCKKSYSTINGLTKHKQFHCE------------
Capitella spI scratch 1                      ------------MPRAFLITNKRY---------------------------------------------------------TVPGQIEEHHPCPDCGKAYSTSSNLARHRQTHRS------------
Capitella spI scratch 2                      ------------MPRSFLVKKR----------PRWSLSD-----------------DDVTTEDGRARRSSLDD--------SSLDDVKAKYTCTECGKQYATSSNLSRHKQTHRS------------
Capitella spI scratch 3                      ------------MPRSFLVKKTER--------------------------------DAFFVSDGRSRKRKPTQ--------EEPKTTGGKFVCNECGKQYATSSNLSRHKQTHRS------------
Capitella spI scratch 4                      ------------MPRSFLIKK-HA---------------------------------------------------------KPTQDVKPKYTCTECGKQYATSSNLSRHKQTHRS------------
Ciona intestinalis snail                     ---------------------------------------------------------------------------------QPVSFKPCRIPCTECGRTYATIGALAKHAKTHED------------
Cupiennius salei snail                       ------------MPRAFLIKKKQQ---------------------------------------------------------AASKPNPTRYQCPDCNKSYSTYSGLSKHRLMHCA------------
Danio rerio scratch 1                        ------------MPRSFLVKKVK-------LDDFSSSDLE-------AVSEG-YTVDAFFITDGRSRRKAISS--------ISSPRTLQRHTCNECGKTYATSSNLSRHKQTHRS------------
Danio rerio scratch 2                        ------------MPRSFLVKKVK-------LDDFSSSELE-------AVSEG-YTVDAFFITDGRSRRKVISG--------ISGSRTLQRHTCNECGKTYATSSNLSRHKQTHRS------------
Danio rerio scratch 3                        ------------MPRSFLVKKIKL-----------------------SLSEG-YTMDAFFISDGRSRRKGEVS--------VGVNNGGARHTCNECGKTYATSSNLSRHKQTHRS------------
Danio rerio snail 1a                         ------------MPRSFLVKKYFTS-----KRP-NYSELE---------------------------------------------------------------------------------------
Danio rerio snail 1b                         ------------MPRSFLVKKYFTN-----KKP-NYSELE-----------------------------------------PPSPDPSDRFQCAHCGKSCSSPAALSRHQLAHCSPQDGISGATSSL
Danio rerio snail 2                          ------------MPRSFLVKKHFNAA----KKP-NYSELE-----------------------------------------STKLSDAEKFQCGLCNKSYSTYSGLMKHKQLHCD------------
Danio rerio snail 3                          ------------MPRSFLVKKHLTN-----KKP-DYGVLD-----------------------------------------TTGNSQEHSDECFDCQKAYLSFSNLANQRQVHCQ------------
Daphnia pulex snail                          ------------MPRSFLVKKNNYSHCPLKKRPLSYFLQE-----------------------------------------SAGTTTTARYQCPDCHKSYSTYCGLTKHQELHCAAQQ---------
Daphnia pulex scratch 1                      ------------MPRCFMPKKHLK-----------------------HKTVA-YTYEAFFVSDGRSKRRNNPS--------PNPSSRSGRYVCGECGKAYATSSNLSRHKQTHRS------------
Daphnia pulex scratch 2                      ---------------------------------------------------------------------------------DNGDGHHRRYVCGECGKAYATSSNLSRHKQTHRN------------
Daphnia pulex scratch 3                      ---------------------------------------------------------------------------------GDSHSGPDGHECPDCGKRYSTSSNLARHRQTHRS------------
Drosophila melanogaster CG15269              ---------------------------------------------------------------------------------VHRGTKEFAAECPECGKTFNDKGYLSSHLKIHR-------------
Drosophila melanogaster snail                ------------MAA-------NYKSCPLKKRPIVFVEER-----------------------------------------AANHAKNYRFKCDECQKMYSTSMGLSKHRQFHCPAAEC--------
Drosophila melanogaster worniu               -------M---D--------KLKYSRCPLKKRPI-MVEES-----------------------------------------MQKPVGVPRYHCQDCGKSYSTYSGLSKHQQFHCPSAEG--------
Drosophila melanogaster escargot             MHTVED-MLVE----------KNYSKCPLKKRPVNYQFEA-----------------------------------------KKDKNQPPRYQCPDCQKSYSTFSGLTKHQQFHCPAAEG--------
Drosophila melanogaster scratch 1            ------------MPRCLIAKKWKAY-------PWLDRTED-------AKTVA-YTYEAFFVSDGRSKRKHVAD--------PTPDQQKTKYTCSECGKQYATSSNLSRHKQTHRS------------
Drosophila melanogaster scratch 2            -------MSV-D----FILPNRNK---------------------------------CLNMLDGRTKASKAAA--------NYAKRKRGCYKCCECGKQYATSSNLSRHKQTHRS------------
Drosophila melanogaster scratch 3            ---------------------------------------------------------------------------------NSTSLLEDEHICPECGKKYSTSSNLARHRQTHRS------------
Drosophila pseudoobscura CG15269             ---------------------------------------------------------------------------------VHRGTKEFAAECPECGKTFNDKGYLSSHMKIHR-------------
Drosophila pseudoobscura snail               ------------MAA-------NYKSCPLKKRPFVFVEEH-----------------------------------------ASSAAKNYRFKCDQCQKMYSTSIGLSKHRQFHCPAAEC--------
Drosophila pseudoobscura worniu              -------M---D--------KLKYSKCPLKKRPIPI-EDA-----------------------------------------MQKPVGVPRYHCQDCGKSYSTYSGLSKHQQFHCPSAEG--------
Drosophila pseudoobscura escargot            MHTVED-MLVE----------KNYSKCPLKKRPVNYQFEA-----------------------------------------AKKDKSPPRYQCPDCQKSYSTFSGLTKHQQFHCPAAEG--------
Drosophila pseudoobscura scratch 1           ------------MPRCLIAKKWKAY-------PWLDRSED-------AKTVA-YTYEAFFVSDGRSKRKHVAD--------VVQDQQKTKYTCSECGKQYATSSNLSRHKQTHRS------------
Drosophila pseudoobscura scratch 2           -------MSV-D----FILPNRNK---------------------------------CLNMMDGRTKASKAAA--------NYAKRKRGCYKCSECGKQYATSSNLSRHKQTHRS------------
Drosophila pseudoobscura scratch 3           ---------------------------------------------------------------------------------HQEQATEDEHICPECGKKYSTSSNLARHRQTHRS------------
Halocynthia roretzi snail                    ---------------------------------------------------------------------------------KPINFKPCRFHCSECGRTYATVGGLMKHSKYHHD------------
Homo sapiens snail 1                         ------------MPRSFLVRKPSDPN----RKP-NYSELQ---------------------------------------------------------------------------------------
Homo sapiens snail 1-like                    ------------MPRSFLVRKLPDPD----RKP-NYSKLP---------------------------------------------------------------------------------------
Homo sapiens snail 2                         ------------MPRSFLVKKHFNAS----KKP-NYSELD-----------------------------------------DPHAIEAEKFQCNLCNKTYSTFSGLAKHKQLHCD------------
Homo sapiens snail 3                         ------------MPRSFLVKTHSSH-----RVP-NYRRLE-----------------------------------------ERMPRAPGGFECFHCHKPYHTLAGLARHRQLHCH------------
Homo sapiens scratch 1                       ------------MPRSFLVKKVK-------LDAFSSADLE-------AVSEG-YAADAFFITDGRSRRKASNA--------PGAAGAGGRHACGECGKTYATSSNLSRHKQTHRS------------
Homo sapiens scratch 2                       ------------MPRSFLVKKIKG-----------------------AVTDS-YSMDAFFISDGRSRRRRGGG--------GAQAGGGHRHACAECGKTYATSSNLSRHKQTHRS------------
Lottia gigantea snail 1                      ------------MPRAFLIKKR-D---------------------------------------------------------KSKENEPIRYQCDSCKKSYSTFSGLSKHKQFHCA------------
Lottia gigantea snail 2                      ---------------------------------------------------------------------------------SINGQSPPRFQCDACQKSYSTFGGLSKHKQFHCA------------
Lottia gigantea scratch 1                    ------------MPRIFMITNRRY---------------------------------------------------------PHDHQDDDPRDCLDCVK-YSSRSSPSHHRPISRT------------
Lottia gigantea scratch 2                    ------------MPKSFLVKKSKY---------------------------------GFIVVDGRTKHRQNGS--------RQNGSPRQRFRCDECGKDYATSSNLSRHKQTHRS------------
Lytechinus variegatus snail                  ------------MPRSFLVKKNA-------KQAA-SG-LK-----------------------------------------NKNGEDSTKYHCPDCGKEYSTFGGLSKHRQLHCD------------
Mus musculus snail 1                         ------------MPRSFLVRKPSDPR----RKP-NYSELQ---------------------------------------------------------------------------------------
Mus musculus snail 2                         ------------MPRSFLVKKHFNAS----KKP-NYSELD-----------------------------------------DPHAIEAEKFQCNLCNKTYSTFSGLAKHKQLHCD------------
Mus musculus snail 3                         ------------MPRSFLVKTHSSH-----RVP-NYGKLE-----------------------------------------EGTSRVPGSFECIHCHRPYHTLAGLARHQQLHCH------------
Mus musculus scratch 1                       ------------MPRSFLVKKVK-------LDTFSSADLD-------AVSEG-YAADAFFITDGRSRRKAANA--------SGATGAGGRHACGECGKTYATSSNLSRHKQTHRS------------
Mus musculus scratch 2                       ------------MPRSFLVKKIKA-----------------------AVTDS-YSMDAFFISDGRSRRRRAGA--------GATAGGGHRHACAECGKTYATSSNLSRHKQTHRS------------
Nasonia vitripennis CG15269                  ---------------------------------------------------------------------------------VHEGTREFAASCPECGKYFNDRGYLSSHMKIHR-------------
Nasonia vitripennis snail                    -------MLVDEMPRSFIKKNNSYSHCPLKKRPVNV-VDD-----------------------------------------SSNGSSSPRYQCPDCAKSYSTYSGLSKHQQFHCAAAE---------
Nasonia vitripennis scratch 1                ------------MPRCLMAKKWKAY-------PWPDRAEE-------NKTVA-YTYEAFFVSDGRSKRRSSN---------AAGQPDRPKFTCTECGKQYATSSNLSRHKQTHRS------------
Nasonia vitripennis scratch 2                ------------MPRAFLITRRRY---------------------------------------------------------QQAQDGAEEHECPDCGKKYSTSSNLARHRQTHRS------------
Nematostella vectensis snail 1               ------------MPRSFLVKTKT----------------------------------------------------------VVTSEAPAKLQCPNCMKGFNALATLMRHQYFYCPP-----------
Nematostella vectensis snail 2               ------------MPRSFLVKKTCD---------------------------------------------------------LTAEESRPKHQCHQCNKGYSTPLGLAKHQQFHCN------------
Nematostella vectensis scratch               ------------MPKSFLIKKKSK---------------------------------KSVCADGRSKGNGGNG--------GGNGGKRSRYVCAECGKSYATSSNLSRHKQTHRS------------
Patella vulgata snail 1                      ---------------------------------------------------------------------------------QLNAHSPPRYQCEACQKSYSTFGGLSKHRQFHCS------------
Patella vulgata snail 2                      ------------MPRAFLIKKR-D---------------------------------------------------------KSKENEPIRYQCDSCKKSYSTFSGLSKHKQFHCA------------
Platynereis dumerilii snail 1                ------------MPKSFLIKKHGA---------------------------------------------------------SNPAKGPPRFNCEGCGKTYATFSGLSKHKRFHCV------------
Platynereis dumerilii snail 2                ---------------------------------------------------------------------------------ENVRRDISRFKCEDCSKSYATFSGLTKHKQFHCS------------
Saccoglossus kowalevskii snail               ------------MPRSFLIKKHCN---------------------------------------------------------RSSNGEHTRYQCTECSKSYSTFSGLTKHKQFHCV------------
Saccoglossus kowalevskii scratch             ------------MPRSFLVKITKS--------------------------------DALLITDGRSRK-------------GSDEQNRQRYKCNECGKHYATSSNLSRHKQTHRS------------
Strongylocentrotus purpuratus snail          ------------MPRSFLVKKNA-------KQAA-SG-LK-----------------------------------------NKNGEDSTKYHCPDCGKEYSTFGGLSKHRQLHCD------------
Strongylocentrotus purpuratus scratch 1      ------------MPRSFLVKKTKF--------------------------------EAFLITDGRSRRRALQN--------LAAVANRPKYKCNECGKQYATSSNLSRHKQTHRS------------
Strongylocentrotus purpuratus scratch 2      ------------MPRSFLVRRVSR---------------------------------------------------------TSPSTMMPGHSCPECRKSYSTSSNLARHRQTHRS------------
Tribolium castaneum CG15269                  ---------------------------------------------------------------------------------VHEGTKEFAAKCPECGKNFNDRGYLSSHMKIHR-------------
Tribolium castaneum snail                    ----MLDMPGTIMA-------KNYSHCPLKKRPVQMLEDI-----------------------------------------SEKRRQGVRHQCPDCGKSYSTFSGLSKHRQFHCAAGE---------
Tribolium castaneum scratch 1                ------------MPRCLMAKKWKAY-------PWPERPEQ-------GKTIA-YTYEAFFVSDGRSKKRFTP---------KALVNEKPKYTCSECGKQYATSSNLSRHKQTHRS------------
Tribolium castaneum scratch 2                ------------MPRCYMVKKQSN---------------------------------------------------------TTTTTKLNRYSCGECGRQYATSSNLSRHKQTHRS------------
Tribolium castaneum scratch 3                ------------MPRAFLITHRRY---------------------------------------------------------DGVLGESNDHECNDCGKRYSTSSNLARHRQTHRS------------
Trichoplax adhaerens snail                   ------------MPRSFLVKKKA--------------------------------------------------------------------------------------------------------
Trichoplax adhaerens scratch                 ------------MPKVFLIKKRKL---------------------------------------------------------NSSNGKKSRYTCAECGKQYATSSNLSRHKQTHRS------------
Xenopus laevis snail 1                       ------------MPRSFLVKKHFSAS----KKP-NYSELE-----------------------------------------ASSATEAEKFQCNLCSKSYSTFAGLSKHKQLHCD------------
Xenopus laevis snail 2a                      ------------MPRSFLVKKHFNSA----KKP-NYGELD-----------------------------------------DSHAIEAEKFQCSLCSKTYSTFSGLAKHKQLHCD------------
Xenopus laevis snail 2b                      ------------MPRSFLVKKHFNTT----KKP-NYGELD-----------------------------------------DSHAIEAEKFQCSLCSKTYSTFSGLAKHKQLHCD------------
```

```
Acropora millepora snail                --TRKEFACKHCDKNYLSLGALKMHIRTHT--LPCKCTICGKAFSRPWLLQGHIRTHTGEKPYQCPKCQRAFADRSNLRAHLQTHSSVKKYSCSQCSRSFSRMSLLVKHQ----YSC-GNEV-
Aedes aegypti CG15269                   --NKKEYACPHCPKSFNQRVAFNMHVRIHTGVKPHKCNECGKRFSRKMLLKQHLRTHSGEKPYQCSVCGKSFADRSNMTLHHRLHSGIKPFACPICPKAFTKKHHLKTHLNYH-TGY-KPYKC
Aedes aegypti snail                     NQAQKIFVCKECDKPYKTLGALKMHIRTHT--LPCKCNLCDKAFSRPWLLQGHIRTHTGEKPFICKLCSRAFADRSNLRAHQQTHEDVKRFKCPSCTKSFSRLPLLTKHTE---SGC-PGIQL
Anolis carolinensis snail 1             SQARKSFSCKYCEKEYVSLGALKMHIRSHT--LPCVCKICGKAFSRPWLLQGHIRTHTGEKPFSCTHCNRAFADRSNLRAHLQTHSDVKKYQCKTCSRTFSRMSLLHKHEE---TGC-TGTR-
Anolis carolinensis snail 2             AQSRKSFSCKYCDKEYVSLGALKMHIRTHT--LPCVCKICGKAFSRPWLLQGHIRTHTGEKPFSCPHCNRAFADRSNLRAHLQTHSDVKKYQCKNCSKTFSRMSLLHKHEE---SGC-CVAH-
Anopheles gambiae scratch 1             LDSQSAKKCHTCGKAYVSMPALAMHVLTHK--LSHSCGVCGKLFSRPWLLQGHLRSHTGEKPYGCAHCGKAFADRSNLRAHMQTHSIDKNYQCGRCHKTFALKSYLNKHLE---SAC-FKEDG
Anopheles gambiae scratch 2             LDSQSAKKCVTCGKAYVSMPALAMHLLTHK--LSHSCGVCGKLFSRPWLLQGHLRSHTGEKPYGCGHCGKAFADRSNLRAHMQTHSTDKNFECGRCHKTFALKSYLNKHLE---SAC-YKDDD
Anopheles gambiae scratch 3             LEDQKARRCPYCSKVYVSMPAYSMHVRTHD--QGSKCPTCDKRFSRPWLLQGHIRTHTGEKPFKCSVCSKAFADKSNLRAHVQTHSNTKPYQCGRCGKSFALKSYLCKHED---SSC-LKNDK
Anopheles gambiae snail                 NQAQKTFVCKECDKPYKTLGALKMHIRTHT--LPCKCTHCDKAFSRPWLLQGHIRTHTGEKPFVCKLCSRAFADRSNLRAHQQTHEDVKRFKCPTCTKSFSRLPLLTKHAE---SGC-PGGSG
Apis mellifera snail                    GQAKKSFSCKYCEKVYVSLGALKMHIRTHT--LPCKCHLCGKAFSRPWLLQGHIRTHTGEKPFSCQHCNRAFADRSNLRAHLQTHSDVKKYSCTSCSKTFSRMSLLTKHQE---GGC-PGITV
Apis mellifera scratch 1                LDSQSAKKCIHCGKAYVSMPALAMHVLTHK--LAHSCGVCGKMFSRPWLLQGHLRSHTGEKPYGCAHCGKAFADRSNLRAHMQTHSADKNYECSRCHKTFALKSYLNKHLE---SAC-LRDDE
Apis mellifera scratch 2                IDSQSAKKCIHCGKAYVSMPALAMHVLTHK--LTHSCGVCGKMFSRPWLLQGHLRSHTGEKPYGCAHCGKAFADRSNLRAHMQTHSADKNYECHKCHKSFALKSYLNKHLE---SAC-QREND
Apis mellifera scratch 3                LGDKKARRCPHCDKVYVSMPAFSMHVRTHN--QGCKCNYCGKCFSRPWLLQGHIRTHTGEKPFKCTICNKAFADKSNLRAHIQTHSNTKPHVCSRCGKAFALKSYLYKHEE---SSC-MRAHH
Branchiostoma floridae snail            TQSKKAFNCKYCDKVYVSLGALKMHIRTHT--LPCKCKICGKAFSRPWLLQGHVRTHTGEKPFACPHCSRAFADRSNLRAHLQTHSDIKKYSCKNCSKTFSRMSLLTKHEE---AGC-CLPNH
Branchiostoma floridae scratch          QQDKKARKCPHCDKVYVSMPALSMHIRTHK--QGCKCPYCGKCFSRPWLLQGHIRTHTGERPFVCQICEKAFADKSNLRAHTQTHSDAKPYVCGRCGQAFALKSYLYKHEE---SAC-LRSSG
Branchiostoma floridae scratch          LDSKLAKTCPTCGKVYVSMPALSMHVLTHQ--LSHKCDICNKAFSRPWLLQGHMRSHTGEKPFGCAHCGKAFADRSNLRAHMQTHSAFKQYKCKRCNKSFALKSYLNKHYE---SAC-FKQQ-
Caenorhabditis elegans CES1              LDSPHAKQCPHCDRVYVSMPALSMHILTHN--ASHECNVCGKRFSRLWLLQGHLRSHTGLRPFSCAHCGKSFADRSNLRAHMLTHTGDKRFECDKCGRRFALRAYLNRHLE---TCK------
Caenorhabditis elegans K02D7.2          -EGKLQQSCPHCKKVYRSPGALKMHLKTHS--LPCVCNDCGKSFSRPWLLKGHLRTHTGEKPFGCEFCGRCFADRSNLRAHLQTHSGEKKHRCSRCGQSFARVQVRQRHEQ-----C-CRGGG
Capitella spI snail 1                   THIKKEFSCKYCDKTYTSLGALKMHIRTHT--LPCKCKLCGKAFSRPWLLQGHIRTHTGEKPFQCAHCGRAFADRSNLRAHLQTHSEVKKYRCKTCAKTFSRMSLLIKHQE---SNC-PGLRP
Capitella spI snail 2                   DMMKKEFSCKYCDKTYTSLGALKMHIRTHT--LPCKCKLCGKAFSRPWLLQGHIRTHTGEKPFQCAHCGRAFADRSNLRAHLQTHSEVKKYRCGRCSKTFARMSLLVKHEG---REC-LSSI-
Capitella spI scratch 1                 ISDKKARKCPHCDKVYVSMPAYSMHVRTHS--QGCQCPYCGKRFSRPWLLQGHIRTHTGEKPFSCQQCGKAFADKSNLRAHVQTHSTEKPYSCGRCGKAFALKSYLYKHEE---SSC-MRGQR
Capitella spI scratch 2                 LDSQQAKKCPHCNKVYVSMPALSMHILTHN--LKHECPVCNKTFSRPWLLQGHMRSHTGEKPYGCAHCGKAFADRSNLRAHMQTHSTFKHYECTRCSKTFALKSYLNKHCE---SAC-PKIDF
Capitella spI scratch 3                 LDSQQAKKCPHCNKVYVSMPALSMHILTHN--LKHECPVCNKTFSRPWLLQGHMRSHTGEKPYGCAHCGKAFADRSNLRAHMQTHSTFKHYECTRCSKTFALKSYLNKHCE---SAC-SKDDT
Capitella spI scratch 4                 LDSQQAKKCPHCNKVYVSMPALSMHILTHN--LKHECPVCNKTFSRPWLLQGHMRSHTGEKPYGCAHCGKAFADRSNLRAHMQTHSTFKHYECARCSKTFALKSYLNKHHE---SSC-LKDEG
Ciona intestinalis snail                PESGSKFNCKICKKECSSLGALRMHIRTHT--LPCECHICGKAFSRTWLLQGHIRTHTGEKPYQCTVCSRAFADRSNLRAHMQTHETVKRYSCVTCEKTFSRISLLKRHQ----VHCETASQV
Cupiennius salei snail                  TQAKKSFGCKYCDKVYVSLGALKMHIRTHT--LPCKCKLCGKAFSRPWLLQGHIRTHTGEKPFSCPHCSRAFADRSNLRAHLQTHSEVKKYRCKTCSKTFSRMSLLLKHED---GGC-AGAAA
Danio rerio scratch 1                   LDSKMAKKCPTCGKVYVSMPAMAMHLLTHD--LKHKCDICGKAFSRPWLLQGHMRSHTGEKPFGCAHCGKAFADRSNLRAHMQTHSAFKHYKCKRCNKTFALKSYLNKHYE---SAC-FKGAF
Danio rerio scratch 2                   LDSKMAKKCPTCGKVYVSMPAMAMHLLTHD--LKHKCDICGKAFSRPWLLQGHMRSHTGEKPFGCAHCGKAFADRSNLRAHMQTHSAFKHFKCKRCNKTFVLKSYLNKHYE---SAC-FKGAF
Danio rerio scratch 3                   LDSKMARKCPTCDKVYVSMPALAMHILTHD--LKHKCHVCSKAFSRPWLLQGHMRSHTGEKPFACAHCGKAFADRSNLRAHMQTHSAFKHYSCKRCNKTFALKSYLNKHYE---SAC-FRGSG
Danio rerio snail 1a                    AASRPAFFCKHCPKEYNSLGALKMHIRSHT--LPCVCPTCGKAFSRPWLLRGHIRTHTGERPFSCPHCNRAFADRSNLRAHLQTHADVKKYQCSTCSRTFSRMSLLQKHSA---AGC-CPSTA
Danio rerio snail 1b                    TSSRAAFHCKHCPKEYNSLGALKMHIRSHT--LPCVCSTCGKAFSRPWLLRGHIRTHTGERPFSCPHCNRAFADRSNLRAHLQTHSEVKKYQCGSCSRTFSRMSLLHKHTL---SGC-CPAL-
Danio rerio snail 2                     AQSRKSFSCKYCEKEYVSLGALKMHIRTHT--LPCVCKMCGKAFSRPWLLQGHIRTHTGEKPFSCPHCSRAFADRSNLRAHLQTHSDVKKYQCKNCSKTFSRMSLLHKHEE---SGC-CIAH-
Danio rerio snail 3                     WPCHKYFTCKYCEKEYVSLGALKMHIRTHT--LPCVCKLCGKAFSRPWLLQGHIRTHTGEKPFTCPHCSRAFADRSNLRAHLQTHSEIKKYQCRNCFKTFSRISLLTKHEE---AGC-CPMS-
Daphnia pulex snail                     AANKKSFSCKHCEKVYVSLGALKMHIRTHT--LPCKCLLCGKAFSRPWLLQGHIRTHTGEKPFSCPHCSRSFADRSNLRAHLQTHSDVKRYSCKSCGKTFSRMSLLSKHED---GGCGCGGAS
Daphnia pulex scratch 1                 LESGCARTCPTCGKAYVSMPALSMHLLTHA--LSHVCPVCSKAFSRPWLLQGHMRSHTGEKPYGCAHCGKAFADRSNLRAHMQTHSATKSHHCGKCHKAFALKSYLNKHLE---SSC-FRDSP
Daphnia pulex scratch 2                 LESGGSKPCPTCGKTYVSMPALSMHLLTHA--LTHVCPVCSKAFSRPWLLQGHMRSHTGEKPYECHLCYKAFADRSNLRAHMQTHSSVKSFRCGQCHKTFALKSYLHKHQETSANCC-VKT--
Daphnia pulex scratch 3                 PADQKARRCPHCDKVYVSVPAFSMHVRTHS--QGCKCPYCGKSFSRPWLLQGHIRTHTGEKPFTCQICEKAFADKSNLRAHIQTHSNLKPFTCQRCGKAFALKSYLYKHEE---SSC-MKLVT
Drosophila melanogaster CG15269          --NRKEYECPYCPKSFNQRVAFNMHVRIHTGVKPHKCNECGKRFSRKMLLKQHMRTHSGEKPYQCSVCGKSFADRSNMTLHHRLHSGIKPFSCPLCPKAFTKKHHLKTHLNYH-TGC-KPYVC
Drosophila melanogaster snail            NQEKKTHSCEECGKLYTTIGALKMHIRTHT--LPCKCPICGKAFSRPWLLQGHIRTHTGEKPFQCPDCPRSFADRSNLRAHQQTHVDVKKYACQVCHKSFSRMSLLNKHSS---SNC-TITIA
Drosophila melanogaster worniu           NQVKKVFSCKNCDKTYVSLGALKMHIRTHT--LPCKCPICGKAFSRPWLLQGHIRTHTGEKPFSCQHCNRAFADRSNLRAHMQTHSDVKKYSCPTCTKSFSRMSLLAKHLQ---SGC-QTEQS
Drosophila melanogaster escargot         NQVKKSFSCKDCDKTYVSLGALKMHIRTHT--LPCKCNLCGKAFSRPWLLQGHIRTHTGEKPFSCQHCHRAFADRSNLRAHLQTHSDIKKYSCTSCSKTFSRMSLLTKHSE---GGC-PGGSA
Drosophila melanogaster scratch 1        LDSQSAKKCHTCGKAYVSMPALAMHLLTHK--LSHSCGVCGKLFSRPWLLQGHLRSHTGEKPYGCAHCGKAFADRSNLRAHMQTHSVDKNFECKRCHKTFALKSYLNKHLE---SAC-LKDEE
Drosophila melanogaster scratch 2        LDSQSAKKCNTCGKAYVSMPALAMHLLTHK--LSHSCDICGKLFSRPWLLQGHLRSHTGEKPYACVHCGKAFADRSNLRAHMQTHSGDKNFKCHRCNKTFALKSYLNKHLE---SAC-LRDAG
Drosophila melanogaster scratch 3        IMDKKARHCPYCEKVYVSMPAYSMHVRTHN--QGCECQFCGKRFSRPWLLQGHIRTHTGEKPFKCGVCEKAFADKSNLRAHIQTHSNTKPHTCARCGKAFALKSYLYKHEE---SSC-MKNRG
Drosophila pseudoobscura CG15269         --NRKEYECPYCPKSFNQRVAFNMHVRIHTGVKPHKCSECGKRFSRKMLLKQHMRTHSGEKPYQCSVCGKSFADRSNMTLHHRLHSGIKPFSCPLCPKAFTKKHHLKTHLNYH-TGC-KPYVC
Drosophila pseudoobscura snail           NQEKKTHSCEECGKLYTTIGALKMHIRTHT--LPCKCPICGKAFSRPWLLQGHIRTHTGEKPFQCPDCPRSFADRSNLRAHQQTHVDVKKYACQVCHKSFSRMSLLNKHSA---SNC-TITIA
Drosophila pseudoobscura worniu          NQVKKVFSCKNCDKTYVSLGALKMHIRTHT--LPCKCPICGKAFSRPWLLQGHIRTHTGEKPFSCQHCNRAFADRSNLRAHMQTHSDVKKYSCPTCTKSFSRMSLLAKHLQ---GGC-HSDAV
Drosophila pseudoobscura escargot        NQVKKSFSCKDCDKTYVSLGALKMHIRTHT--LPCKCNMCGKAFSRPWLLQGHIRTHTGEKPFSCQHCHRAFADRSNLRAHLQTHSDIKKYSCSSCSKTFSRMSLLTKHSE---GGC-PGGSS
Drosophila pseudoobscura scratch 1       LDSQSGKKCHTCGKAYVSMPAPAMHVLIHK--LSHSCGVCGKLFSRPWLLQGHLRSHTGEKPYGCAHCGKAFADRSNLRAHMQTHSVDKNFECKRCHKTFALKSYLNKHLE---SAC-LKDEE
Drosophila pseudoobscura scratch 2       LDSQSAKKCNTCGKAYVSMPALAMHLLTHK--LSHSCDICGKLFSRPWLLQGHLRSHTGEKPYACVHCGKAFADRSNLRAHMQTHSGDKNFKCHRCNKTFALKSYLNKHLE---SAC-LRDAG
Drosophila pseudoobscura scratch 3       IMDKKARHCPFCEKVYVSMPAYSMHVRTHS--QGCECHHCGKCFSRPWLLQGHIRTHTGEKPFKCSVCSKAFADKSNLRAHIQTHSNTKPHSCSRCGKAFALKSYLYKHEE---SSC-MKNRS
Halocynthia roretzi snail                PENAMTFKCKTCEKEYTSLGALKMHIRTHT--LPCKCHICGKAFSRTWLLQGHIRTHTGEKPYQCSVCLRAFADRSNLRAHMQTHQNVKRYACTGCEKTFSRTSLLNRHRA---SGC-VP---
Homo sapiens snail 1                     LQARKAFNCKYCNKEYLSLGALKMHIRSHT--LPCVCGTCGKAFSRPWLLQGHVRTHTGEKPFSCPHCSRAFADRSNLRAHLQTHSDVKKYQCQACARTFSRMSLLHKHQE---SGC-SGCPR
Homo sapiens snail 1-like                SQFQKAFNCQYCNKECLSLGALKKHIRSHT--LPFACETCGKAFSRPWLLRGHVRTHTGEKPFSCTHCSRAFADRSNLRAHLQTHLDVKKYQCQACARTFSRMALLHKHQE---SGC-SGGPR
Homo sapiens snail 2                     AQSRKSFSCKYCDKEYVSLGALKMHIRTHT--LPCVCKICGKAFSRPWLLQGHIRTHTGEKPFSCPHCNRAFADRSNLRAHLQTHSDVKKYQCKNCSKTFSRMSLLHKHEE---SGC-CVAH-
Homo sapiens snail 3                     LQVGRVFTCKYCDKEYTSLGALKMHIRTHT--LPCTCKICGKAFSRPWLLQGHVRTHTGEKPYACSHCSRAFADRSNLRAHLQTHSDAKKYRCRRCTKTFSRMSLLARHEE---SGC-CPGP-
Homo sapiens scratch 1                   LDSQLARRCPTCGKVYVSMPAMAMHLLTHD--LRHKCGVCGKAFSRPWLLQGHMRSHTGEKPFGCAHCGKAFADRSNLRAHMQTHSAFKHFQCKRCKKSFALKSYLNKHYE---SAC-FKGGA
Homo sapiens scratch 2                   LDSQLARKCPTCGKAYVSMPALAMHLLTHN--LRHKCGVCGKAFSRPWLLQGHMRSHTGEKPFGCAHCGKAFADRSNLRAHMQTHSAFKHYRCRQCDKSFALKSYLHKHCE---AAC-AKAAE
Lottia gigantea snail 1                  SQIKKEFNCKYCDKTYVSLGALKMHIRTHT--LPCKCKLCGKAFSRPWLLQGHIRTHTGEKPFSCQHCGRAFADRSNLRAHLQTHSDIKKYSCRGCSKTFSRMSLLLKHED---SCC-GPVPH
Lottia gigantea snail 2                  QQIKKDFRCKYCDKSYSSLGALKMHIRTHT--LPCKCKLCGKAFSRPWLLQGHIRTHTGEKPFSCQHCGRAFADRSNLRAHLQTHAEIKKYGCKNCSKTFSRMSLLVKHGE---SSC-MGIVR
Lottia gigantea scratch 1                ANDKKARKCPHCDKVYVSMPAYSMHVRTHN--QGCECVYCGKKFSRPWLLQGHIRTHTGEKPFSCPKCGKAFADKSNLRAHIQTHSTEKPYICGRCGKAFALKSYLYKHEE---SSC-MRGQR
Lottia gigantea scratch 2                LDSQLAKKCPHCEKVYVSMPALSMHILTHS--QRHICNICGKTFSRPWLLRGHIRSHSGQKPFGCSHCGKAFADRSNLRAHMNTHSAFKQFQCQYCKKTFALKSYLNKHHE---SMC-LK---
Lytechinus variegatus snail              AQNKKTFNCKYCDKEYMSLGALKMHIRTHT--LPCKCKFCGKAFSRPWLLQGHIRTHTGEKPFSCPHCQRAFADRSNLRAHLQTHSEVKKYSCKSCGKTFSRMSLLNKHEE---SGC-ISSGS
Mus musculus snail 1                     PQSRKIFNCKYCNKEYLSLGALKMHIRSHT--LPCVCTTCGKAFSRPWLLQGHVRTHTGEKPFSCSHCNRAFADRSNLRAHLQTHSDVKRYQCQACARTFSRMSLLHKHQE---SGC-SGGPR
Mus musculus snail 2                     AQSRKSFSCKYCDKEYVSLGALKMHIRTHT--LPCVCKICGKAFSRPWLLQGHIRTHTGEKPFSCPHCNRAFADRSNLRAHLQTHSDVKKYQCKNCSKTFSRMSLLHKHEE---SGC-CVAH-
Mus musculus snail 3                     LPTGRAFTCRYCDKEYASLGALKMHIRTHT--LPCICKVCGKAFSRPWLLQGHIRTHTGEKPYTCSHCSRAFADRSNLRAHLQTHVGTKKYRCAVCPKAFSRMSLLARHEE---AGC-CPGP-
Mus musculus scratch 1                   LDSQLARRCPTCGKVYVSMPAMAMHLLTHD--LRHKCGVCGKAFSRPWLLQGHMRSHTGEKPFGCAHCGKAFADRSNLRAHMQTHSAFKHFQCKRCKKSFALKSYLNKHYE---SAC-FKGGA
Mus musculus scratch 2                   LDSQLARKCPTCGKAYVSMPALAMHVLTHN--LRHKCGVCGKAFSRPWLLQGHMRSHTGEKPFGCAHCGKAFADRSNLRAHMQTHSAFKHYRCRQCDKSFALKSYLHKHCE---AAC-VKAAE
Nasonia vitripennis CG15269              --NRKEYACAECGKSFNQRVAYNMHVRIHTGLKPHQCEQCGKAFSRKMLLKQHLRTHSGERPYQCQVCHKAFADRSNMTLHTRLHSGLKPYQCTLCSKAFTKKHHLKTHLNYH-TGT-KPYSC
Nasonia vitripennis snail                GQTKKTFTCKYCEKVYLTLGALKMHIRTHT--LPCKCHLCGKAFSRPWLLQGHIRTHTGEKPFSCQHCNRAFADRSNLRAHLQTHSDVKKYSCSSCSKTFSRMSLLTKHQE---GGC-PGGSR
Nasonia vitripennis scratch 1            LDSQSAKKCIHCGKAYVSMPALAMHVLTHK--LAHSCGVCGKMFSRPWLLQGHLRSHTGEKPYGCAHCGKAFADRSNLRAHMQTHSADKNYECSRCHKTFALKSYLNKHLE---SAC-LRDDE
Nasonia vitripennis scratch 2            LGDKKARRCPHCDKVYVSIPAFSMHVRTHN--QGCKCHFCGKCFSRPWLLQGHIRTHTGEKPFKCTICNKAFADKSNLRAHIQTHSNTKPHVCGRCGKAFALKSYLYKHEE---SSCMPRLEH
Nematostella vectensis snail 1           TQHKRPFHCKYCEKLYDSLGALKMHIRTHT--LPCKCKICGKAFSRPWLLQGHVRTHTGEKPYKCTQCQRAFADRSNLRAHLQTHSDVKKYSCKQCSKSFSRMSLLLKHE----GSC-SGASS
Nematostella vectensis snail 2           THHKKSFTCKHCDKIYVSLGALKMHIRTHT--LPCKCSICGKAFSRPWLLQGHIRTHTGEKPYQCTNCKRAFADRSNLRAHMQTHAVVKKYSCSRCKKSFSRMSLLVEHED---SGC-PSQG-
Nematostella vectensis scratch           LDGKLAKRCHQCGKAYVSMPALAMHVLTHK--LLHKCEICGKSFSRPWLLQGHKRSHTGERPYSCPECNKAFADRSNLRAHLQTHNPLKQYKCERCDRTFALKSYLNKHLE---SAC-SRDSS
Patella vulgata snail 1                  QQVKKEFRCKYCDKSYSSLGALKMHIRTHT--LPCKCKLCGKAFSRPWLLQGHIRTHTGEKPFSCQHCGRAFADRSNLRAHLQTHAEIKKYGCKSCSKTFSRMSLLLKHGE---SSC-MGMVR
Patella vulgata snail 2                  SQIKKEFNCKYCDKTYVSLGALKMHIRTHT--LPCKCKLCGKAFSRPWLLQGHIRTHTGEKPFSCQHCGRAFADRSNLRAHLQTHSDVKKYSCRSCSKTFSRMSLLLKHED---GCC-GTVVH
Platynereis dumerilii snail 1            SEIKKEFSCKFCDKTYSSLGALKMHIRTHT--LPCKCPLCGKAFSRPWCLQGHIRTHTGEKPFNCNHCSRAFADRSNLRAHLQTHTDIKKYSFKSCSKTFSRMSLLIKHED---GGC-PGMSS
Platynereis dumerilii snail 2            ALFKKEFSCKYCSKTYVSLGALKMHVRTHT--LPCKCPLCGKAFSRPWLLQGHIRTHTGEKPFNCNHCSRAFADRSNLRAHLQTHTDIKKYNCKNCPKTFSRMSLLLNLKMEDALHI-YGIPK
Saccoglossus kowalevskii snail           TQAKKSFSCKYCEKEYLSLGALKMHIRTHT--LPCKCKICGKAFSRPWLLQGHIRTHTGENPFACTYCNRAFADRSNLRAHLQTHSDVKKYCCKSCSKTFSRMSLLTKHEE---AGC-CSVTS
Saccoglossus kowalevskii scratch         LDSKLAKKCPTCDKVYVSMPALAMHVLTHN--LKHRCSICTKSFSRPWLLQGHMRSHTGEKPFGCAHCGKAFADRSNLRAHMQTHSAYKNYECKRCNKSFALKSYLNKHYE---SAC-FKGMQ
Strongylocentrotus purpuratus snail      AQNKKTFNCKYCDKEYMSLGALKMHIRTHT--LPCKCKFCGKAFSRPWLLQGHIRTHTGEKPFSCPHCQRAFADRSNLRAHLQTHSEVKKYSCKSCGKTFSRMSLLNKHEE---SGC-ISSSS
Strongylocentrotus purpuratus scratch 1  LDSHLAKKCEVCNKVYVSMPALAMHVLTHN--LKHKCNVCHKSFSRPWLLQGHMRSHTGEKPFGCAHCGKAFADRSNLRAHMQTHSAYKNYKCKRCDKSFALKSYLNKHYE---SAC-FKLDG
Strongylocentrotus purpuratus scratch 2  VTDQKARKCPHCEKVYVSMPALSMHIRTHK--LGCKCHICGKCFSRPWLLQGHIRTHTGERPFSCPQCGKAFADKSNLRAHVQTHSTIKPYVCQKCNKAFALKSYLYKHVE---SAC-YRAT-
Tribolium castaneum CG15269              --DRKEYACPHCPKRFNQRVAYNMHLRIHTGIKPHECPTCGKSFSRKMLLKQHQRVHTGERPYSCPECGKTFADRSNMSLHARLHTGVKPYSCNLCSKSFTKKHHLKTHMNFH-TGL-KPYTC
Tribolium castaneum snail                -GPKKSFSCKFCEKVYVSLGALKMHIRTHT--LPCKCTICGKAFSRPWLLQGHIRTHTGEKPFSCTYCNRAFADRSNLRAHLQTHSDVKKYSCPTCSKTFSRMSLLTKHAE---GGC-SGVHQ
Tribolium castaneum scratch 1            LDSQSAKKCITCGKAYVSMPALAMHVLTHK--LAHRCSVCGKQFSRPWLLQGHLRSHTGEKPYGCAHCGKAFADRSNLRAHMQTHSADKNFECPRCHKTFALKSYLNKHLE---SAC-LKEEA
Tribolium castaneum scratch 2            LDSQSAKKCMTCGKAYVSMPALAMHLLTHK--LAHSCGVCGKQFSRPWLLQGHLRSHTGEKPYGCAHCGKAFADRSNLRAHMQTHSGDKNFSCPQCHKTFALKSYLNKHQE---TACLLWDDK
Tribolium castaneum scratch 3            PGDKKARRCPHCDKLYVSMPAFSMHVRTHN--QGCKCQYCGKCFSRPWLLQGHIRTHTGEKPFKCTICNKAFADKSNLRAHIQTHSNTKPHICGRCGKAFALKSYLYKHEE---SSC-MRING
Trichoplax adhaerens snail               YHDGKNFVCKHCNKVYASLGALKMHIRTHT--LPCKCKICGKAFSRPWLLQGHIRTHTGEKPFGCEKCNRSFADRSNLRAHMQTHAEIKKYSCYKCRRTFSRWSSLKKHEN---SS-------
Trichoplax adhaerens scratch             LDGQLARRCKYCDKAYVSMPALAMHVLTHE--LAHKCNICGKAFSRSWLLQGHMRSHTGEKPYACATCNKRFADRSNLRAHMQTHSSVKSFQCKNCGKSFALKSYLNKHAE---SGC-CKNRG
Xenopus laevis snail 1                   SQTRKSFSCKYCEKEYVSLGALKMHIRSHT--LPCVCKICGKAFSRPWLLQGHIRTHTGEKPFSCTHCNRAFADRSNLRAHLQTHSDVKKYQCKSCSRTFSRMSLLHKHEE---TGC-TVAH-
Xenopus laevis snail 2a                  AQSRKSFSCKYCEKEYVSLGALKMHIRTHT--LPCVCKICGKAFSRPWLLQGHIRTHTGEKPFSCPHCNRAFADRSNLRAHLQTHSDVKKYQCKNCSKTFSRMSLLHKHEE---SGC-CVAH-
Xenopus laevis snail 2b                  VQSRKSFSCKYCEKEYVSLGALKMHIRTHT--LPCVCKICGKAFSRPWLLQGHIRTHTGEKPFSCPHCNRAFADRSNLRAHLQTHSDVKKYQCKNCSKTFSRMSLLHKHEE---SGC-CVAH-
```
