# Supplementary material for: Insights into the evolution of the snail superfamily from metazoan wide molecular phylogenies and expression data in annelids
Source: BMC Evol Biol. 2009 May 9;9:94. doi: 10.1186/1471-2148-9-94 (PMC2688512; doi:10.1186/1471-2148-9-94)
Supplement: Additional file 3 — Genomic localization of the snail and scratch genes in species whose genome is completely sequenced [file 1471-2148-9-94-S3.pdf]

Species	Gene name	Chromosome or Scaffold	Position	Orientation	Family	Database	Protein size
Anopheles gambiae	*scratch 1*	2L	38,386,587..38,446,604	Reverse	Scratch B	Vectorbase	797
Anopheles gambiae	*scratch 2*	2L	38,539,647..38,543,818	Forward	Scratch B	Vectorbase	721
Anopheles gambiae	*scratch 3*	2L	14,856,841..14,858,937	Reverse	Scratch A	Vectorbase	415
Anopheles gambiae	*snail*	3R	8,432,372..8,430,480	Reverse	Snail	Vectorbase	522
Anopheles gambiae	*CG15269*	3R	47,339,542..47,349,775	Reverse	CG15269	Vectorbase	428
Apis mellifera	*scratch 1*	LG1	13,560,418..13,588,732	Forward	Scratch B	NCBI	544
Apis mellifera	*scratch 2*	LG1	13,522,460..13,526,961	Reverse	Scratch B	NCBI	615
Apis mellifera	*snail*	LG13	5,722,634..5,724,362	Reverse	Snail	NCBI	509
Apis mellifera	*scratch 3*	LG7	9,309,432..9,325,479	Forward	Scratch A	NCBI	525
Apis mellifera	*CG15269*	LG9	6,278,268..6,293,503	Reverse	CG15269	NCBI	924
Branchiostoma floridae	*scratch 1*	Bf_V2_Scaffold_187	6,035,267..6,040,734	Forward	Scratch A	Jgi	307
Branchiostoma floridae	*scratch 2*	Bf_V2_Scaffold_95	414,012..415,783	Forward	Scratch B	Jgi	276
Branchiostoma floridae	*snail*	Bf_V2_Scaffold_187	325,246..327,056	Forward	Snail	Jgi	253
Caenorhabditis elegans	*CES1*	Chr.I	8,633,942..8,636,034	Forward	Scratch B	UCSC	270
Caenorhabditis elegans	*K02D7.2*	Chr.IV	311,555..313,697	Reverse	Snail	UCSC	193
Capitella sp.	*scratch 1*	Scaffold_56	326,545..329,175	Reverse	Scratch A	Jgi	445
Capitella sp.	*scratch 2*	Scaffold_6104	4,149..5,917	Reverse	Scratch B	Jgi	264
Capitella sp.	*scratch 3*	Scaffold_1257	7,097..9520	Forward	Scratch B	Jgi	241
Capitella sp.	*scratch 4*	Scaffold_1231	32,044..34,062	Forward	Scratch B	Jgi	355
Capitella sp.	*snail 1*	Scaffold_64	232,265..234,539	Reverse	Snail	Jgi	449
Capitella sp.	*snail 2*	Scaffold_64	240,723..244,368	Forward	Snail	Jgi	405
Ciona intestinalis	*snail*	Chr.3q	4,122,498..4,125,423	Reverse	Snail	Jgi	584
Danio rerio	*scratch 1*	Not localized (NW_001881293.1)	20,183..22,019	Reverse	Scratch B	NCBI	279
Danio rerio	*scratch 2*	Chr.16	780,807..782,446	Reverse	Scratch B	NCBI	279
Danio rerio	*scratch 3*	Chr.8	20,658,214..20,651,766	Reverse	Scratch B	NCBI	312
Danio rerio	*snail 1a*	Chr.11	1,344,062..1,348,453	Reverse	Snail	NCBI	263
Danio rerio	*snail 1b*	Chr.23	10,996..17,326	Reverse	Snail	NCBI	256
Danio rerio	*snail 2*	Chr.24	107,444..111,242	Reverse	Snail (Slug)	NCBI	257
Danio rerio	*snail 3*	Chr.7	59,292..66,129	Forward	Snail	NCBI	283
Daphnia pulex	*snail*	Scaffold_23	1,247,868..1,249,529	Forward	Snail	Jgi	518
Daphnia pulex	*scratch 1*	Scaffold_110	236,503..239,638	Forward	Scratch B	Jgi	444
Daphnia pulex	*scratch 2*	Scaffold_110	193,850..194,317	Reverse	Scratch B	Jgi	156
Daphnia pulex	*scratch 3*	Scaffold_39	954,167..955,428	Forward	Scratch A	Jgi	376
Drosophila melanogaster	*CG15269*	2L	15,109,585..15,112,914	Forward	CG15269	Flybase	587
Drosophila melanogaster	*escargot*	2L	15,333,864..15,336,150	Forward	Snail	Flybase	470
Drosophila melanogaster	*snail*	2L	15,476,593..15,478,269	Reverse	Snail	Flybase	390
Drosophila melanogaster	*worniu*	2L	15,423,293..15,425,585	Reverse	Snail	Flybase	548
Drosophila melanogaster	*scratch - 3 - (CG17181)*	3L	582,824..589,272	Reverse	Scratch A	Flybase	442
Drosophila melanogaster	*scratch - 1 - CG1130*	3L	3,983,916..3,988,589	Forward	Scratch B	Flybase	653
Drosophila melanogaster	*scratch - 2 - CG12605*	3L	3,956,841..3,964,771	Reverse	Scratch B	Flybase	619
Drosophila pseudoobscura	*CG15269*	4	2,490,981..2,494,045	Reverse	CG15269	Flybase	599
Drosophila pseudoobscura	*snail*	4	2,337,637..2,338,866	Forward	Snail	Flybase	410
Drosophila pseudoobscura	*worniu*	4	2,378,827..2,380,485	Forward	Snail	Flybase	553
Drosophila pseudoobscura	*escargot*	4	2,465,787..2,467,283	Reverse	Snail	Flybase	480

Drosophila pseudoobscura	*scratch - 3 - GA14370*	XR_group3a	784,341..785,650	Forward	Scratch A	Flybase	401
Drosophila pseudoobscura	*scratch - 2 - GA11715*	XR_group8	603,266..607,411	Reverse	Scratch B	Flybase	630
Drosophila pseudoobscura	*scratch - 1 - (CG1130)*	XR_group8	632,466..635,064	Forward	Scratch B	Flybase	701
Homo sapiens	*snail 1*	chromosome 20q13.1	13,652,505..13,657,501	Forward	Snail	NCBI	258
Homo sapiens	*snail 1 like*	chromosome 2q34	36,074,757..36,075,521	Forward	Snail	NCBI	264
Homo sapiens	*snail 2*	chromosome 8q11	1,684,718..1,687,176	Reverse	Snail (Slug)	NCBI	268
Homo sapiens	*snail 3*	chromosome 16	305,473..313,431	Reverse	Snail	NCBI	292
Homo sapiens	*scratch 1*	chromosome 8q24.3	124,259..127,243	Reverse	Scratch B	NCBI	348
Homo sapiens	*scratch 2*	chromosome 20p12.3–13	584,315..596,245	Reverse	Scratch B	NCBI	307
Lottia gigantea	*scratch 1*	Scaffold_56	709,306..716,969	Forward	Scratch A	Jgi	335
Lottia gigantea	*scratch 2*	Scaffold_56	1,248,568..1,250,618	Reverse	Scratch B	Jgi	338
Lottia gigantea	*snail 1*	Scaffold_19	701,193..704,146	Reverse	Snail	Jgi	441
Lottia gigantea	*snail 2*	Scaffold_19	675,111..676,196	Forward	Snail	Jgi	369
Mus musculus	*snail 1*	chromosome 2-97.0	108,405,092..108,408,885	Forward	Snail	NCBI	264
Mus musculus	*snail 2*	chromosome 16-9.4	11,706,067..11,708,389	Forward	Snail (Slug)	NCBI	269
Mus musculus	*snail 3*	chromosome 8	49,812,107..49,817,868	Reverse	Snail	NCBI	287
Mus musculus	*scratch 1*	chromosome 15	37,635,176..37,638,139	Reverse	Scratch B	NCBI	348
Mus musculus	*scratch 2*	chromosome 2	649,588..664,474	Forward	Scratch B	NCBI	497
Nasonia vitripennis	*CG15269*	Un - Scaffold-5	2,222,539..2,225,772	Reverse	CG15269	NCBI	485
Nasonia vitripennis	*scratch 2*	Un - Scaffold-39	480,629..487,182	Reverse	Scratch A	NCBI	354
Nasonia vitripennis	*scratch*	Un - Scaffold-61	778,205..785,302	Reverse	Scratch B	NCBI	484
Nasonia vitripennis	*snail*	Un - Scaffold-110	399,745..401,359	Reverse	Snail	NCBI	490
Nematostella vectensis	*scratch*	Scaffold_89	833,317..834,260	Forward	Scratch B/A	Jgi	289
Nematostella vectensis	*snail 1*	Scaffold_5	2,127,094..2,128,083	Reverse	Snail	Jgi	272
Nematostella vectensis	*snail 2*	Scaffold_32	1,315,979..1,317,038	Forward	Snail	Jgi	265
Strongylocentrotus purpuratus	*scratch 1*	Scaffold_4299	79,296..93,844	Forward	Scratch B	NCBI	332
Strongylocentrotus purpuratus	*scratch 2*	Scaffold_70608	18,351..38,736	Forward	Scratch A	NCBI	306
Strongylocentrotus purpuratus	*snail*	Scaffold_78497	2,289..4,733	Reverse	Snail	NCBI	341
Tribolium castaneum	*CG15269*	LG5	5,195,598..5,208,012	Reverse	CG15269	Flybase	469
Tribolium castaneum	*scratch 3*	LG3	12,134,676..12,143,135	Reverse	Scratch A	Flybase	364
Tribolium castaneum	*scratch 1*	UG8	109,163..118,426	Forward	Scratch B	Flybase	421
Tribolium castaneum	*scratch 2*	UG8	58,033..87,013	Reverse	Scratch B	Flybase	478
Tribolium castaneum	*snail*	LG5	13,492,299..13,493,353	Forward	Snail	Flybase	332
Trichoplax adhaerens	*snail*	Scaffold_1	10,520,414..10,523,467	Forward	Snail	Jgi	251
Trichoplax adhaerens	*scratch*	Scaffold_1	10,529,527..10,530,994	Forward	Scratch B/A	Jgi	389
