# Supplementary figures and images for: Insights into the evolution of the snail superfamily from metazoan wide molecular phylogenies and expression data in annelids
Source: BMC Evol Biol. 2009 May 9;9:94. doi: 10.1186/1471-2148-9-94 (PMC2688512; doi:10.1186/1471-2148-9-94)

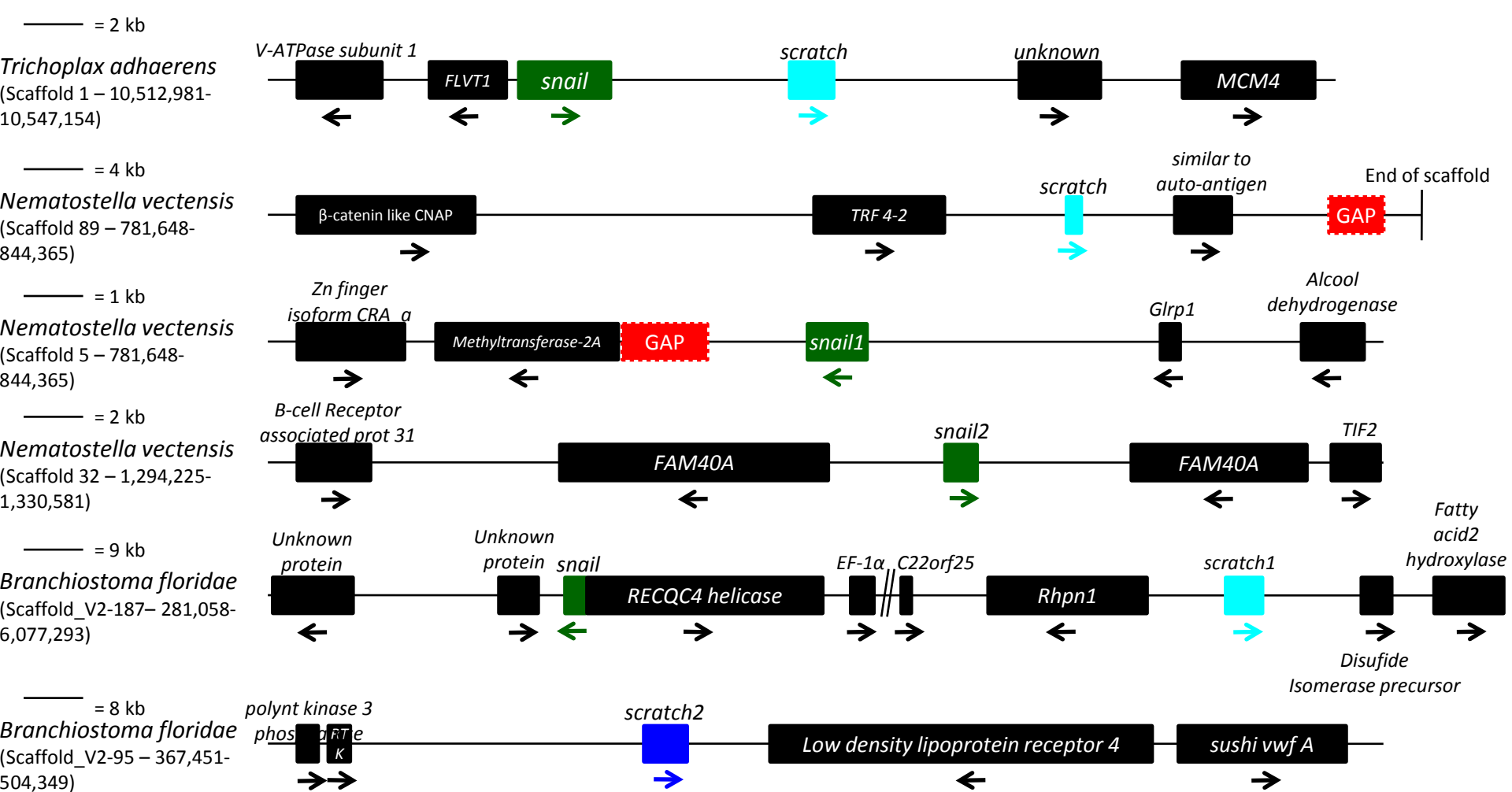

Supplement: Additional file 4 — Maps of the scaffolds that contain the snail and scratch genes in Trichoplax, Nematostella, and Branchiostoma. The portion of the genome that includes the snail and scratch genes is schematically depicted. The name of the genes that flank the snail and scratch genes are those indicated in the genome browsers of the different species. [file 1471-2148-9-94-S4.pdf]
